# Suppressing Stitching Errors in Full‐Sensor‐Plane Color Routers via Optical Structural Similarity

**DOI:** 10.1002/nap2.70220

**Published:** 2026-07-20

**Authors:** Donghyun Kim, Junseo Han, Seunghyun Lee, Min Seok Jang, Haejun Chung

**Affiliations:** ^1^ Department of Electronic Engineering Hanyang University Seoul Republic of Korea; ^2^ Department of Artificial Intelligence Semiconductor Engineering Hanyang University Seoul Republic of Korea; ^3^ School of Electrical Engineering Korea Advanced Institute of Science and Technology Daejeon Republic of Korea; ^4^ Department of Artificial Intelligence Hanyang University Seoul Republic of Korea

**Keywords:** color router, image sensor, inverse design, metasurface, optical structural similarity

## Abstract

Nanophotonic color routers promise higher photon utilization than absorptive color filters for complementary metal‐oxide‐semiconductor (CMOS) image sensors, yet most reported designs remain optimized for normal incidence, with efficiency dropping to about half of its peak by θ≈6°. Here, we present an angle‐specific inverse‐design framework in which the sensor plane is partitioned into local angular zones, and each zone is assigned a dedicated color‐router unit cell. To enable practical assembly of these heterogeneous unit cells, we introduce an optical structural similarity (OSS) constraint that enforces permittivity‐level continuity between neighboring cells during topology optimization without additional full‐wave simulations. In a three‐dimensional freeform library, the optimized unit cells achieve average optical efficiencies of 91.1%, 80.9%, and 83.9% for the red, green, and blue channels, respectively, with optical cross talk below 2.0%. OSS reduces the average stitching error from 24.6% to 5.99%, suggesting that it may provide an effective means of suppressing stitching errors in metasurface designs based on the locally periodic approximation. The framework further extends to a five‐layer architecture with a comparable stitching error of 5.65%, establishing a practical route to oblique‐incidence‐robust, library‐based color routing and, more broadly, to large‐area metasurfaces requiring spatially varying functionality with robust inter‐cell compatibility.

## Graphical abstract

Angle‐specific nanophotonic color routers are assembled from a library of inverse‐designed unit cells matched to local incidence angles. Optical structural similarity (OSS) constrains neighboring permittivity profiles during optimization, suppressing inter‐cell stitching errors without additional full‐wave simulations and enabling oblique‐incidence‐robust color routing for CMOS image sensors.

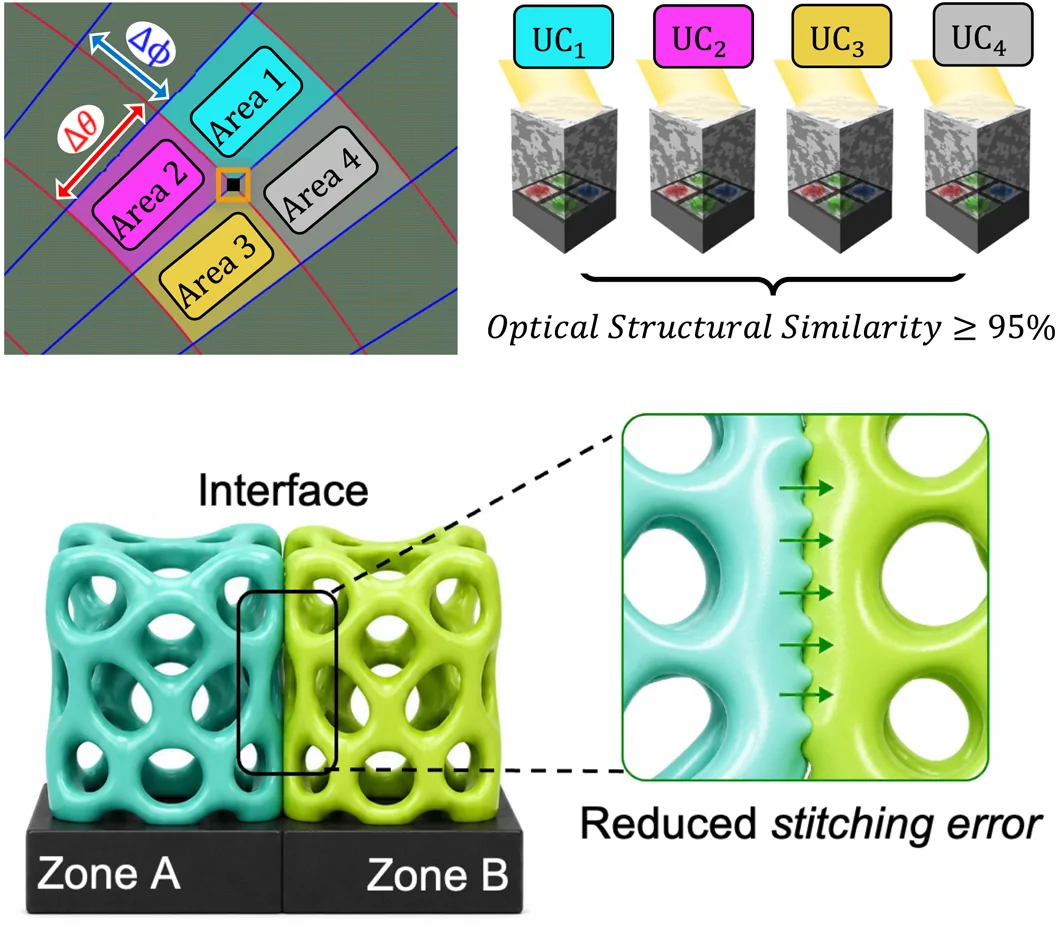

## 1 Introduction

Solid‐state image sensors are the foundation of modern digital imaging systems, enabling applications ranging from mobile photography [1, 2] to autonomous navigation [3, 4] and biomedical diagnostics [5, 6]. Complementary metal‐oxide‐semiconductor (CMOS) image sensors (CISs) have become the dominant technology, owing to their compatibility with mature CMOS fabrication and on‐chip integration [7]. A typical CIS pixel combines a photodetector with a microlens and a color filter, typically arranged in a Bayer mosaic [8]. As pixel pitches continue to shrink and have now reached 0.5μm [9], two limitations become increasingly severe. First, conventional microlenses operate in a wave‐optics regime, where diffraction and interference impose fundamental limits on light concentration and focusing performance [10, 11, 12]. Second, absorptive color filters intrinsically discard out‐of‐band photons; in a Bayer‐mosaic architecture under idealized illumination, the maximum theoretical photon utilization is limited to 25%, 50%, and 25% for the red, green, and blue pixels, respectively. These constraints have motivated the development of *color routers*, which separate spectral components by directing different wavelengths to designated photodetector regions rather than rejecting undesired light through absorption.

Early demonstrations of color routers were largely based on physics‐inspired metasurface concepts, including plasmonic resonances, local phase modulation, and others [13, 14, 15, 16, 17, 18, 19, 20, 21]. However, because these devices were typically constructed from relatively simple parametric elements, such as cylinders or rectangular pillars, the available design space remained limited, thereby constraining achievable routing efficiency. Recent advances in computational power and large‐scale optimization methods have made it increasingly practical to explore high‐dimensional design spaces, leading to growing interest in freeform and inverse‐designed nanophotonic color routers [22, 23, 24, 25, 26, 27, 28, 29, 30, 31, 32, 33, 34, 35, 36, 37, 38, 39]. By introducing complex subwavelength dielectric features with many degrees of freedom, these approaches can harness scattering, near‐field coupling, and resonant effects in a coordinated manner to improve spectral‐routing performance. Indeed, several recent studies have reported high optical efficiencies. For example, a freeform grayscale design achieved efficiencies exceeding 99% at the target color channels [25]; a double‐layer architecture reported efficiencies of approximately 51%, 82%, and 69% for red, green, and blue light, respectively [38]; and a metasurface color router achieved corresponding efficiencies of about 60%, 57%, and 65% [30]. By redistributing photons rather than intrinsically rejecting them, color routers offer a promising approach to substantially improving photon utilization in aggressively scaled CIS architectures.

A major remaining challenge is angular robustness. Most reported nanophotonic color routers are optimized primarily for normal incidence and degrade substantially under oblique illumination [22, 30, 35, 36, 39]. In practical camera modules, however, the incident angle generally increases with radial distance from the optical axis [40] and can exceed 30° near the image periphery in wide‐field‐of‐view systems [41, 42]. Commercial CISs partly compensate for this variation by laterally offsetting the microlens and color‐filter stack relative to the photodetectors [42, 43, 44]. Yet achieving comparable angular tolerance with nanophotonic color routers remains difficult. Camayd‐Muñoz et al. [22] showed that a layered meta‐optic optimized at normal incidence loses roughly half of its peak efficiency by θ=±8°. A similar trend is observed in our own analysis: As shown in Figure 1a, a freeform router optimized only for normal incidence falls to about half of its maximum efficiency near θ=±6° (See Supporting Information S1: Note S1 for details).

**FIGURE 1:**
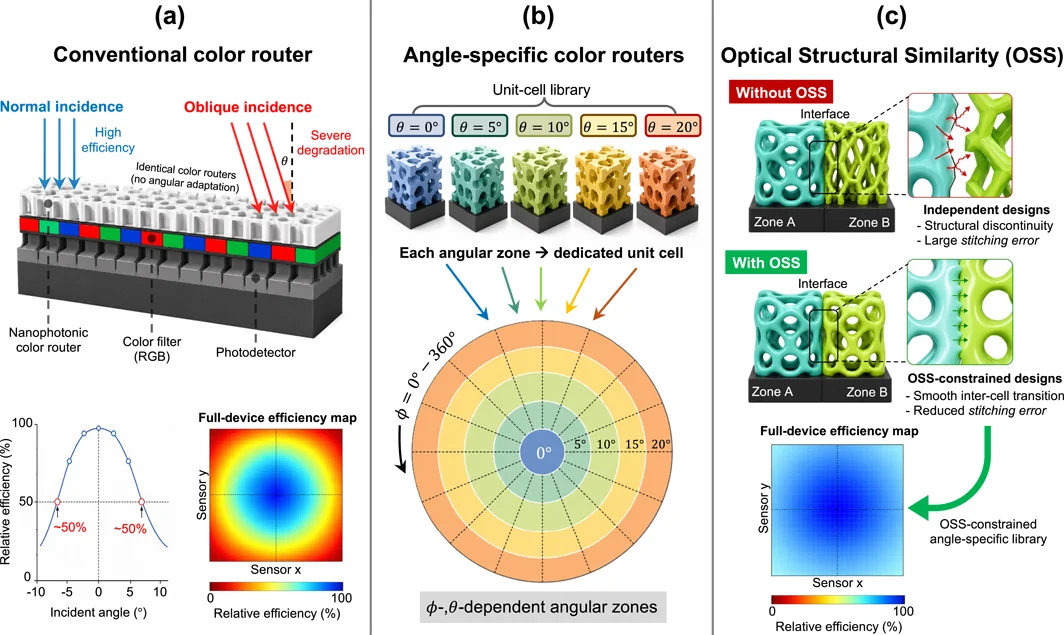
Conceptual overview of angle‐specific nanophotonic color routers with optical structural similarity (OSS)‐constrained stitching for CMOS image sensors. (a) A conventional spatially uniform color‐router array, designed for normal incidence, can efficiently route on‐axis light but suffers substantial efficiency degradation under oblique illumination, resulting in a spatially nonuniform full‐sensor efficiency distribution. (b) In the proposed angle‐specific strategy, the sensor plane is partitioned into local angular zones defined by the incident azimuthal and polar angles, (ϕ,θ). Each zone is assigned a dedicated unit‐cell color router optimized for the corresponding angular range, enabling angle‐adapted color routing across the sensor plane. (c) OSS‐constrained assembly of heterogeneous angle‐specific unit cells. Independently optimized neighboring cells can introduce abrupt permittivity discontinuities at zone interfaces, leading to severe stitching error. By contrast, OSS‐constrained optimization promotes smooth permittivity‐level transitions between adjacent unit cells, thereby suppressing stitching errors and enhancing full‐device efficiency uniformity.

More recently, Jeon et al. [35] improved angular tolerance by optimizing a single freeform router over a continuous polar‐angle range of ±12°, with an average efficiency of 55.9%. Nevertheless, the efficiency still drops to about 40%, and the optical cross talk rises to approximately 30% at θ=±15°. These results point to a practical trade‐off between angular tolerance and peak efficiency. Moreover, extending the optimization to include multiple azimuthal angles introduces additional constraints on the design space, thereby further reducing the maximum attainable efficiency. This empirical trend is also consistent with prior studies on the fundamental performance limits of metasurfaces, which indicate that broadband and wide‐angle functionalities are subject to physical upper bounds [45, 46, 47, 48, 49, 50]. Consequently, simultaneously achieving high efficiency, low cross talk, and wide‐angle operation within a single spatially uniform color‐router design remains intrinsically difficult.

A natural alternative is to partition the sensor plane into discrete *angular zones* and assign each zone an *angle‐specific unit‐cell color router* optimized for its local illumination condition under Bloch boundary conditions, as illustrated in Figure 1b. Because each unit cell then needs to operate over only a limited angular range, this strategy can, in principle, preserve near‐optimal performance across the full sensor plane while relaxing the trade‐off between angular acceptance and peak efficiency.

A key challenge, however, arises in integrating these independently optimized unit cells into a single device. Neighboring angular zones generally require distinct permittivity distributions, which can produce abrupt structural discontinuities at their interfaces, as illustrated in Figure 1c. These discontinuities may generate parasitic scattering and boundary‐induced *stitching errors*, resulting in local image blur and color aberrations near interfaces. A similar limitation has been reported in large‐area metasurfaces designed using the locally periodic approximation (LPA) [46, 51, 52, 53], where independently optimized unit cells are stitched together to form a global device. For volumetric three‐dimensional (3D) color routers, more intricate inter‐cell coupling can further aggravate these stitching errors.

Stitching errors have motivated the development of coupling‐aware strategies for large‐area metasurfaces. The overlapping‐domain approximation of Lin and Johnson [52] improves accuracy by extending each subdomain into neighboring regions and terminating the extended domain with perfectly matched layers (PMLs), thereby capturing interactions that are neglected under the LPA. However, each subproblem remains position‐dependent and typically requires many full‐wave solves, making it difficult to build a compact, reusable unit‐cell library as the overlap or subdomain size increases. Phan et al. [53] reduced the computational burden by optimizing wavelength‐scale finite sections under PML boundary conditions and stitching those sections into a full device, but coupling across stitched interfaces is still not explicitly included, so performance may degrade as section size decreases and the number of interfaces increases. Sun et al. [54] introduced zoned discrete axisymmetry (ZDA) for wide‐aperture 3D metalenses, achieving near‐linear aperture scaling while retaining a larger design space than continuous axisymmetry; nevertheless, ZDA is tailored to radially symmetric devices and does not directly extend to fully 3D, angle‐segmented CIS arrays. In parallel, deep‐learning approaches have been explored to account for inter‐unit‐cell coupling [55, 56], but current models remain focused on finite local neighborhoods and relatively simple wavefront‐shaping tasks. They therefore do not yet offer the predictive fidelity needed for CIS color‐router arrays, where broadband operation, strong coupling, and spatially varying incident‐angle conditions must be handled simultaneously across the sensor plane. These limitations motivate a more direct method for suppressing stitching errors between adjacent angle‐specific unit cells.

In this work, we propose a design paradigm based on an *optical structural similarity* (OSS) constraint that enforces structural continuity between neighboring angle‐specific unit cells during topology optimization. OSS measures the similarity between the unit cell under optimization and a previously optimized neighboring cell and acts as a regularization term until a prescribed similarity threshold is met. As illustrated in Figure 1c, the resulting continuity suppresses stitching errors and yields more spatially uniform efficiency across the sensor plane. In our simulations, the OSS constraint reduces the stitching error at zone boundaries from 24.6% to 5.99%. We validate the proposed framework using 3D freeform color routers and further extend it to multilayer architectures, demonstrating compatibility with practical CIS implementations. Instead of requiring a single‐color router to accommodate a broad angular range, our method decouples angular requirements by using spatially graded unit cells while maintaining smooth inter‐zone transitions via OSS. More generally, OSS establishes a broadly applicable design principle for large‐area metasurfaces with spatially varying functionality by enabling local optimization together with global structural continuity at the permittivity level.

## 2 Design Strategy

This section introduces an angle‐specific design strategy for realizing high‐efficiency color routing over the full CIS domain while preserving smooth transitions between adjacent angular zones. The framework consists of three key steps: (i) The sensor plane is partitioned into angular zones, each assigned a dedicated unit‐cell color router optimized for its corresponding incident angle (Section 2.1); (ii) each unit cell is designed via adjoint‐based topology optimization to maximize the band‐averaged optical efficiency for directing the red, green, and blue spectral bands to their respective photodetectors (Section 2.2); and (iii) to suppress stitching errors arising from the tiling of structurally dissimilar unit cells, an OSS constraint is imposed to regularize the evolving permittivity distribution toward that of previously optimized neighboring cell(s) (Section 2.3).

### 2.1 Angle‐Specific Partitioning

We assume that the sensor plane lies in the xy‐plane, with the optical axis oriented along the +z direction. At each position (x,y) on the sensor plane, the local illumination condition is characterized by a polar angle θ(x,y), defined with respect to the surface normal, and an azimuthal angle ϕ(x,y), measured counterclockwise in the xy‐plane from the +x axis. To represent this continuous angular distribution using a finite and designable library of unit cells, we discretize the (ϕ,θ) space with angular step sizes Δϕ and Δθ. Each region is indexed by its center angle $\left(\phi_{0},\theta_{0}\right)$, and the corresponding *angular zone* on the sensor plane is defined as follows:

(1) $$Z_{\phi_{0},\theta_{0}}=\left\{\left(x,y\right)\;|\;\left|\phi \left(x,y\right)-\phi_{0}\right|\leq \frac{\Delta \phi }{2},\;\left|\theta \left(x,y\right)-\theta_{0}\right|\leq \frac{\Delta \theta }{2}\right\},$$

where the azimuthal difference $\left|\phi \left(x,y\right)-\phi_{0}\right|$ is interpreted modulo 360°. This discretization partitions the sensor plane into a finite set of regions, each associated with a specific incident‐angle condition and thus with a corresponding angle‐specific unit‐cell design.

For each angular zone $Z_{\phi_{0},\theta_{0}}$, we assign a dedicated unit‐cell color router, denoted by ${UC}_{\phi_{0},\theta_{0}}$, which is optimized for illumination centered near $\left(\phi_{0},\theta_{0}\right)$ under Bloch boundary conditions (Section 2.2). The angular discretization steps (Δϕ,Δθ) define a practical trade‐off between angular specificity and the size of the unit‐cell library. Finer angular sampling reduces the range of incident angles that each unit cell must accommodate and can therefore improve angle‐matched performance; however, it also increases the total number of distinct unit cells required to cover the full sensor plane. In this work, we consider two angular grids: Grid 1 with (Δϕ,Δθ)=(10°,4°) and Grid 2 with (Δϕ,Δθ)=(5°,2°). The specific grid used in each case is identified in the corresponding results section.

### 2.2 Adjoint‐Based Topology Optimization

We consider a 3D color router positioned above a 2×2 Bayer‐pattern photodetector array comprising one red pixel, one blue pixel, and two green pixels located at diagonally opposite corners, denoted by $G_{1}$ and $G_{2}$ (Figure 2). The routing performance is quantified by the optical efficiency, defined as the fraction of incident optical power delivered to the designated photodetector region. For a plane wave of wavelength λ incident at azimuthal and polar angles (ϕ,θ), the optical efficiency of color channel i∈{R,G,B} is defined as follows:

(2) $${OE}_{i}\left(\lambda ,\phi ,\theta \right)=\frac{\Phi_{i}\left(\lambda ,\phi ,\theta \right)}{\Phi_{\text{inc}}\left(\lambda ,\phi ,\theta \right)},$$

where $\Phi_{i}\left(\lambda ,\phi ,\theta \right)$ denotes the transmitted electromagnetic power flux integrated over the target photosensitive region associated with color channel i, and $\Phi_{\text{inc}}\left(\lambda ,\phi ,\theta \right)$ is the total incident power flux through the entrance plane of the unit cell. For the green channel, the target region is given by $G_{1}\cup G_{2}$.

**FIGURE 2:**
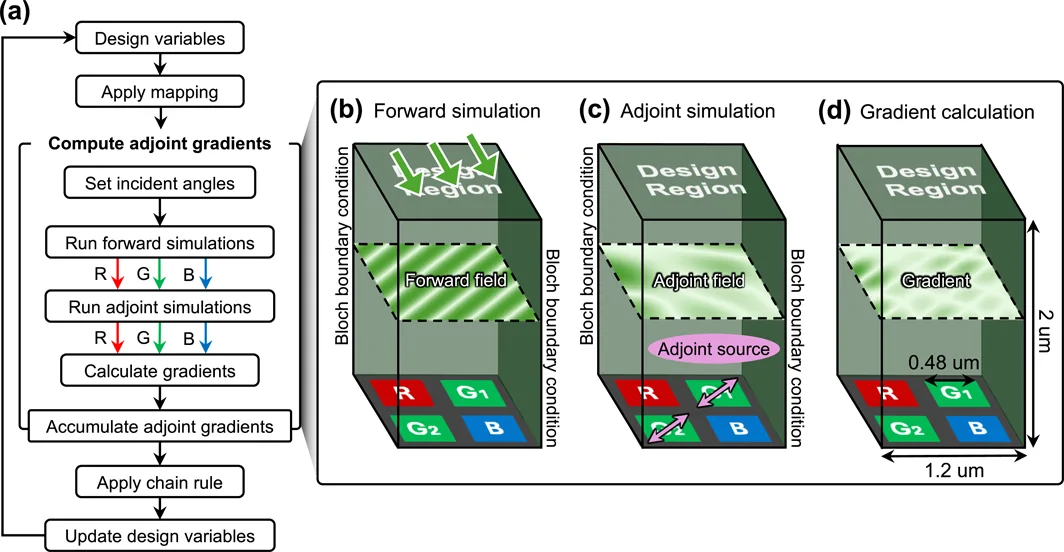
Adjoint‐based topology‐optimization workflow for the angle‐specific 3D color router. (a) Overall iterative optimization loop. Starting from the design variables, a filtering/projection mapping generates the physical permittivity distribution used in the simulations. For the selected incident angle, separate forward simulations are performed for the red, green, and blue spectral channels, followed by the corresponding adjoint simulations. The channel‐wise gradients are then calculated, accumulated, backpropagated through the mapping by the chain rule, and used to update the design variables for the next iteration. The repeated R/G/B labels indicate that the forward and adjoint simulations are carried out independently for each color channel. (b) Forward simulation under Bloch boundary conditions applied on the lateral boundaries. Green arrows denote the obliquely incident plane‐wave excitation illuminating the $1.2\times 1.2\times 2.0\;\mu m^{3}$ design region placed above a 2×2 Bayer‐pattern photodetector array composed of red (R), blue (B), and two green pixels ($G_{1}$ and $G_{2}$). The dashed slice inside the unit cell partially visualizes the forward field distribution. (c) Adjoint simulation in the same unit cell. Pink arrows denote adjoint sources excited from the target photodetector region(s); the schematic shows the green‐channel case, in which the sources are placed at $G_{1}$ and $G_{2}$. The resulting adjoint field is displayed on the same slice plane. (d) Gradient evaluation. The sensitivity of the objective with respect to the permittivity distribution is obtained from the overlap of the forward and adjoint fields and is visualized on the slice plane. The dimensional annotations indicate a unit‐cell pitch of 1.2μm, a design‐region height of 2μm, and a photodetector width of 0.48μm.

Each ${UC}_{\phi_{0},\theta_{0}}$ is optimized to maintain high performance over the full angular extent of $Z_{\phi_{0},\theta_{0}}$. Accordingly, the optimization uses a discrete set of sampled incidence angles $\left\{\left(\phi_{m},\theta_{m}\right)\right\}$. In the optimization loop, we fix the azimuth at $\phi_{m}=\phi_{0}$ and sample the polar angle $\theta_{m}$ at 1° intervals across the θ range of the angular zone. This is motivated by the weak dependence of optical efficiency on ϕ within the azimuthal span of a zone, for example, $\phi_{0}\pm 5{}^{\circ}$ for Grid 1 or $\phi_{0}\pm 2.5{}^{\circ}$ for Grid 2, as shown in Supporting Information S1: Figure S2. For the selected angle pair $\left(\phi_{m},\theta_{m}\right)$, we maximize the optical figure of merit:

(3) $$F_{\text{OE}}=\sum_{i\in \left\{R,G,B\right\}}{OE}_{i}-\gamma {\left({OE}_{G_{1}}-{OE}_{G_{2}}\right)}^{2},$$

where

$${OE}_{i}=\frac{1}{n_{i}}\sum_{k=1}^{n_{i}}{OE}_{i}\left(\lambda_{i,k},\phi_{m},\theta_{m}\right)$$

is the band‐averaged efficiency of color channel i, computed from $n_{i}$ uniformly sampled wavelengths $\lambda_{i,k}$ within the corresponding spectral band. The penalty term enforces a balanced response between the two green pixels, and throughout this work we set γ=0.3. At each iteration, the objective is evaluated for only one sampled angle pair, and the full set is traversed deterministically over successive iterations, as shown in Figure 2a.

The 3D freeform design region occupies $1.2\times 1.2\times 2.0\;\mu m^{3}$ and is discretized into ${\left(20\;nm\right)}^{3}$ cubic voxels, corresponding to approximately $3.6\times 10^{5}$ degrees of freedom. To solve this large‐scale optimization problem efficiently, we employ adjoint‐based topology optimization [57, 58, 59, 60, 61, 62, 63, 64]. Rather than perturbing each voxel independently, the adjoint method obtains the gradient of $F_{\text{OE}}$ with respect to the permittivity distribution from two full‐wave simulations per color channel: (i) a forward simulation under the prescribed illumination (Figure 2b) and (ii) an adjoint simulation excited by sources derived from the objective functional (Figure 2c). The voxel‐wise sensitivity over the entire design region is then computed from the overlap of the forward and adjoint fields (Figure 2d).

To reduce the computational cost of oblique‐incidence optimization, we use band‐centered Bloch excitations for the three spectral channels and a single 45° linearly polarized input during the optimization loop, whereas final device performance is evaluated using wavelength‐resolved simulations at the exact incident‐angle condition. Note that the optical efficiencies reported in this work correspond to 45° linearly polarized incidence and should not be interpreted as unpolarized‐light efficiencies (see Supporting Information S1: Note S2.4). In this work, the color‐router designs are represented by two lossless dielectric materials, silica and silicon nitride, with dielectric constants of $\varepsilon_{r}=2.10$ and $\varepsilon_{r}=4.41$, respectively [65, 66]. The photodetector array is modeled with the same dielectric constant as silicon nitride to reduce computational load; however, this formulation can be readily extended to absorptive materials such as Si, Ge, or InGaAs [67]. The design variables are mapped through filtering and projection to a physical silica/silicon‐nitride permittivity distribution, which enforces a minimum feature size and yields binarized final structures [68, 69]. The design variables are updated using the Adam optimizer [70]. Full implementation details, including the validation of these computational approximations and the sensitivity derivations, are provided in Supporting Information S1: Note S2.

### 2.3 Optical Structural Similarity (OSS)

We introduce an OSS metric that promotes continuity of the permittivity distribution during topology optimization. Let ε(x) denote the mapped permittivity distribution of the current unit cell after filtering and projection, and let $\varepsilon_{\text{ref},j}\left(x\right)$ denote the mapped permittivity distribution of an already‐optimized neighboring unit cell in direction j∈{ϕ,θ}. For each available neighbor direction, the OSS is defined as follows:

(4) $${OSS}_{j}=1-\frac{1}{N{\left(\Delta \varepsilon \right)}^{2}}\sum_{n=1}^{N}{\left[\varepsilon \left(x_{n}\right)-\varepsilon_{\text{ref},j}\left(x_{n}\right)\right]}^{2},\;j\in \left\{\phi ,\theta \right\},$$

where N is the number of design voxels, $x_{n}$ is the position of the n‐th voxel, and $\Delta \varepsilon =\varepsilon_{\text{high}}-\varepsilon_{\text{low}}$ is the permittivity contrast between the two design materials. Because both ε(x) and $\varepsilon_{\text{ref},j}\left(x\right)$ are bounded by the constituent permittivities, Equation (4) satisfies $0\leq {OSS}_{j}\leq 1$, with ${OSS}_{j}=1$ indicating identical permittivity distributions, whereas smaller values indicate larger voxel‐wise structural mismatch.

We incorporate OSS into the same update loop as the optical objective in Equation (3) by defining the total figure of merit as follows:

(5) $$F_{\text{tot}}=F_{\text{OE}}+\sum_{j\in \left\{\phi ,\theta \right\}}\beta_{j}\min\left({OSS}_{j},\tau \right),$$

where τ is a prescribed similarity threshold and $\beta_{j}$ controls the regularization strength along direction j; $\beta_{j}=0$ when the corresponding reference neighbor does not exist. When ${OSS}_{j}<\tau$, the OSS term increases as the current design becomes more similar to the reference cell and therefore suppresses abrupt structural discontinuities between neighboring angular zones. Once ${OSS}_{j}\geq \tau$, the min(⋅) operator saturates the contribution, so the OSS term adds only a constant offset and does not further influence the design update. In this manner, the optimization enforces structural continuity up to a prescribed level of similarity without excessively constraining the optical objective.

A key advantage of OSS is that it depends only on the permittivity distributions and therefore requires no additional full‐wave simulations. Its contribution can be evaluated analytically and incorporated directly into the adjoint gradient. Differentiating Equation (4) yields the following equation:

(6) $$\frac{\partial F_{\text{tot}}}{\partial \varepsilon \left(x\right)}=\frac{\partial F_{\text{OE}}}{\partial \varepsilon \left(x\right)}+\sum_{j\in \left\{\phi ,\theta \right\}}\beta_{j}\left\{\begin{array}{cc} -\frac{2}{N{\left(\Delta \varepsilon \right)}^{2}}\left[\varepsilon \left(x\right)-\varepsilon_{\text{ref},j}\left(x\right)\right], & {OSS}_{j}<\tau ,\\ 0, & {OSS}_{j}\geq \tau , \end{array}\right.$$

where $\partial F_{\text{OE}}/\partial \varepsilon \left(x\right)$ is obtained from the forward–adjoint field overlap. A schematic overview of the per‐neighbor OSS evaluation and its gradient contribution is provided in Figure 3; the same procedure is applied independently to each available neighboring direction, and the resulting contributions are accumulated in the design update. In this formulation, the OSS term acts as a lightweight structural regularizer that suppresses permittivity‐level discontinuities while remaining fully compatible with standard adjoint‐based topology optimization.

**FIGURE 3:**
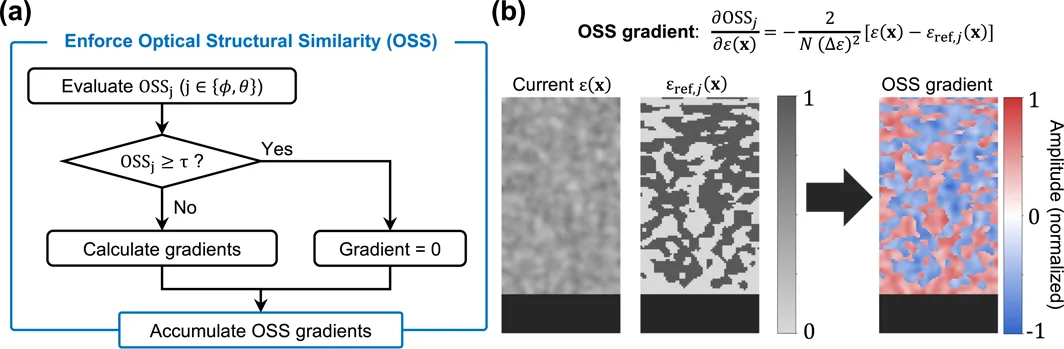
Evaluation of OSS and construction of its analytical gradient. (a) Flowchart of the OSS regularization step used in each optimization iteration. For each available neighboring direction j∈{ϕ,θ}, the similarity metric ${OSS}_{j}$ is evaluated independently and subjected to the threshold test, ${OSS}_{j}\geq \tau$. If the threshold is satisfied, the clipped OSS term is already saturated and the contribution from that neighbor is set to zero. If ${OSS}_{j}<\tau$, the analytical OSS gradient is calculated. The nonzero contributions from all available neighbors are then accumulated and combined with the adjoint sensitivity used for the design update. (b) Spatial interpretation of the analytical gradient contribution associated with a representative OSS term, $\partial {OSS}_{j}/\partial \varepsilon \left(x\right)=-2{\left[N{\left(\Delta \varepsilon \right)}^{2}\right]}^{-1}\left[\varepsilon \left(x\right)-\varepsilon_{\text{ref},j}\left(x\right)\right]$. Representative xz cross‐sections show the current permittivity distribution ε(x) (left), the reference distribution $\varepsilon_{\text{ref},j}\left(x\right)$ (center), and the resulting OSS gradient map (right). The current and reference permittivity fields are depicted on a normalized grayscale scale from 0 to 1, whereas the gradient is shown on a normalized diverging scale from −1 to 1. The gradient field is obtained directly from the voxel‐wise difference between the two permittivity maps. Regions with strong mismatch produce large‐magnitude red and blue responses, indicating opposite local correction directions required to drive the current structure toward the reference and thereby improve structural continuity.

## 3 Results and Discussion

Building on the angle‐specific design framework introduced in Section 2, we first establish stitching compatibility as a key requirement for practical sensor‐plane color routing and then assess how effectively the OSS constraint satisfies this requirement. To separate intrinsic unit‐cell performance from assembly‐induced degradation, we compare isolated and stitched responses in terms of optical efficiency, optical cross talk, and stitching error. We begin with a 2D model that isolates stitching‐induced degradation in independently optimized neighboring cells (Section 3.1), then validate OSS‐constrained 3D freeform libraries across representative multi‐zone boundaries relevant to full sensor‐plane coverage (Section 3.2), and finally extend the same framework to a reduced‐complexity five‐layer architecture to evaluate its practical generality (Section 3.3).

Table 1 places these results in the context of prior color‐router studies. Most previous designs were optimized exclusively for normal incidence or treated oblique illumination only partially, typically without simultaneously accounting for both the polar and azimuthal angles of incidence. By contrast, our framework explicitly incorporates both angular coordinates and, crucially, enforces structural continuity between neighboring angle‐specific unit cells, thereby enabling stitched sensor‐plane assemblies with reduced boundary‐induced degradation.

**TABLE 1:** Comparison with prior studies.

Ref.	Design	Angle consideration		Efficiency	
		θ	ϕ	Normal incidence	Oblique incidence
1[22]	Five‐layer	No	No	57%^a^	Drop to half by ±v8°
2[27]	Single‐layer metasurface	No	No	61, 57, 50%^b^	Severe cross talk at 40° ^e^
3[30]	Single‐layer metasurface	No	No	60, 57, 65%^b^	Drop to half by ±12°
4[36]	Single‐layer metasurface	No	No	49.7, 55.4, 43.2, 61.9%^c^	Stable within ±5° ^e^
5[35]	Freeform volumetric	Yes (−12°≤θ≤12°)	No	64.3%^a^ ^,^ ^d^	55.9%^a^ ^,^ ^d^
This work	Freeform volumetric	Yes (θ≤20°)	**Yes** (0°≤ϕ≤360°) ^g^	**88.8**%^a^	**85.3**%^a^ ^,^ ^f^
	Five‐layer	Yes (θ≤20°)	**Yes** (0°≤ϕ≤45°) ^h^	**71.5**%^a^	**71.1**%^a^ ^,^ ^f^

*Note:* Bold entries highlight the results of this work.

^a^ Average efficiency of three color channels.

^b^ Peak efficiencies of R, G, and B channels, respectively.

^c^ Peak efficiencies of R, G, B, and NIR channels, respectively.

^d^ Average efficiencies were calculated based on Supporting Information S1: Figure S15 in Ref. [5].

^e^ Exact efficiency values were not explicitly reported.

^f^ Average efficiencies were computed over the unit cells explicitly evaluated in the stitched‐supercell study.

^g^ For the freeform library, high‐polar‐angle stitching validation was performed over the representative azimuthal sector 0°≤ϕ≤45° up to $\theta_{0}=20{}^{\circ}$, whereas full 360° azimuthal wrap‐around closure was explicitly constructed and validated for the low‐polar‐angle branches $\theta_{0}\leq 8{}^{\circ}$.

^h^ For the five‐layer library, stitching validation was performed over the representative azimuthal sector 0°≤ϕ≤45° up to $\theta_{0}=20{}^{\circ}$.

### 3.1 Stitching Errors in Independently Optimized Two‐Dimensional Angle‐Specific Color Routers

To numerically analyze stitching error, we consider a computationally tractable 2D model to examine whether stitching alone can induce substantial performance degradation. A rigorous evaluation of the fully stitched structure would require full‐wave simulation of the entire device. As a practical alternative, we model a single stitched interface using an elongated 2D supercell comprising 20 repeated unit cells on each side of the interface. This configuration ensures that the interface‐adjacent cells are sufficiently separated from the supercell boundaries, thereby minimizing artifacts arising from the outer simulation domain (See Supporting Information S1: Note S3 for details). Extending this buffer region to three dimensions would require a supercell on the order of 40×40 unit cells in the xy plane, corresponding to a lateral domain exceeding 80λ×80λ. For the volumetric 3D structures considered in this work, wavelength‐resolved full‐wave simulation over such a domain would be computationally intensive. The 2D model thus provides an efficient and physically transparent testbed for isolating stitching‐induced degradation prior to addressing the practical 3D angle‐specific color‐routing problem.

In this 2D formulation, the color‐routing structure is modeled in the xz plane with TE plane‐wave illumination, where each angular zone is represented by a 2D unit‐cell color router optimized for a specific polar angle of incidence. As shown in Figure 4a, the design region is 1.5μm wide and 2.0μm high and is discretized into ${\left(20\;nm\right)}^{2}$ pixels. The light‐gray and dark‐gray regions denote silica and silicon nitride, respectively. Beneath the design region, three laterally arranged photodetectors corresponding to the red, green, and blue pixels collect the transmitted optical power. Each photodetector has a width of 0.38μm, and adjacent photosensitive regions are separated by 0.12μm‐wide deep‐trench‐isolation regions.

**FIGURE 4:**
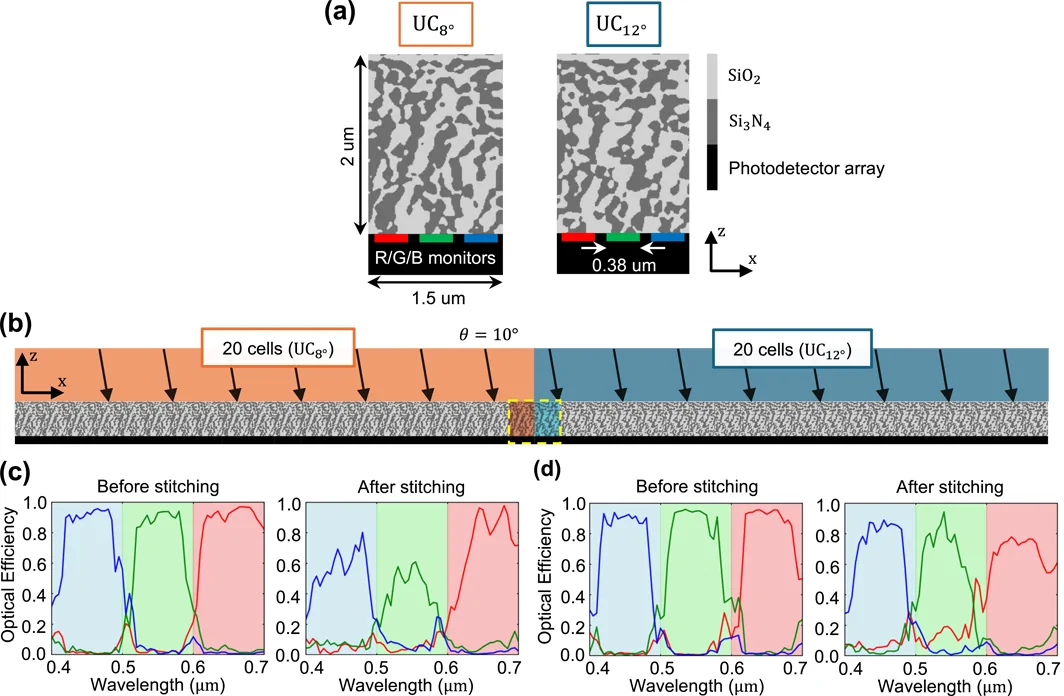
Two‐dimensional validation of stitching‐induced degradation in independently optimized angle‐specific color routers. (a) Independently optimized 2D unit cells, ${UC}_{8{}^{\circ}}$ and ${UC}_{12{}^{\circ}}$, for adjacent angular zones centered at θ=8° and θ=12° with Δθ=4°. (b) Long 2D supercell formed by concatenating 20 copies each of ${UC}_{8{}^{\circ}}$ and ${UC}_{12{}^{\circ}}$ across a single inter‐zone boundary and illuminated at the boundary angle $\theta_{\text{inc}}=10{}^{\circ}$. (c, d) Optical‐efficiency spectra of the two cells adjacent to the stitched interface, compared with the corresponding isolated Bloch‐periodic responses at the same incidence angle. For the interface‐adjacent ${UC}_{8{}^{\circ}}$ cell, the average optical efficiencies in the red, green, and blue channels decrease from 93.2%, 90.0%, and 92.3% to –81.9%, 49.1%, and 60.0%, respectively. For the interface‐adjacent ${UC}_{12{}^{\circ}}$ cell, the corresponding values decrease from 93.4%, 91.5%, and 89.9% to –73.5%, 71.0%, and 83.7%, respectively. The corresponding spectrally normalized $L_{2}$ errors are 37.8% for ${UC}_{8{}^{\circ}}$ and 28.9% for ${UC}_{12{}^{\circ}}$.

We consider two neighboring angular zones with a step size of Δθ=4°, centered at θ=8° and θ=12°. The corresponding independently optimized unit cells, denoted as ${UC}_{8{}^{\circ}}$ and ${UC}_{12{}^{\circ}}$, are shown in Figure 4a. *Before stitching*, both unit cells exhibit strong performance within their respective angular ranges. Specifically, ${UC}_{8{}^{\circ}}$ achieves average optical efficiencies of 95.62%, 91.04%, and 92.95% for the red, green, and blue channels, respectively, with corresponding average optical crosstalk values of 1.09%, 1.92%, and 1.18%. Similarly, ${UC}_{12{}^{\circ}}$ attains average optical efficiencies of 95.21%, 91.88%, and 91.23%, with corresponding average optical crosstalk values of 1.08%, 1.42%, and 1.01% (See Supporting Information S1: Figures S7 and S8 for details). We note that these values are averaged over the angular range assigned to each unit cell and are therefore not directly comparable to the “*before stitching*” results presented below, which are evaluated at the boundary angle θ=10° to provide a consistent baseline for comparison with the stitched supercell.

We next construct the elongated supercell shown in Figure 4b by concatenating 20 copies of ${UC}_{8{}^{\circ}}$ and 20 copies of ${UC}_{12{}^{\circ}}$, thereby forming a single inter‐zone boundary. The supercell is illuminated at θ=10°, corresponding to the boundary angle between the two angular zones. For a consistent comparison, the “*before stitching*” spectra in Figure 4c,d correspond to the isolated Bloch‐periodic responses of ${UC}_{8{}^{\circ}}$ and ${UC}_{12{}^{\circ}}$ evaluated at the same incident angle, whereas the “*after stitching*” spectra are extracted from the two unit cells immediately adjacent to the stitched interface within the supercell. For quantitative comparison, we define the stitching error as follows:

(7) $$e_{\text{stitching}}=\frac{{\left\|\eta^{\text{after}}-\eta^{\text{before}}\right\|}_{2}}{{\left\|\eta^{\text{before}}\right\|}_{2}}\times 100\%,$$

where $\eta =\left[{OE}_{R}\left(\lambda \right);\;{OE}_{G}\left(\lambda \right);\;{OE}_{B}\left(\lambda \right)\right]$ denotes the concatenated optical‐efficiency vector evaluated at n uniformly sampled wavelengths λ across the visible range. All stitching errors reported in this work are evaluated using Equation (7), including the two‐dimensional analysis in this section (Section 3.1), the three‐dimensional freeform libraries (Section 3.2), and the five‐layer architecture (Section 3.3).

The results reveal pronounced degradation in both interface‐adjacent cells *after stitching*. For the ${UC}_{8{}^{\circ}}$ cell, the average optical efficiencies in the red, green, and blue channels decrease from 93.2%, 90.0%, and 92.3% to 81.9%, 49.1%, and 60.0%, respectively, as shown in Figure 4c. For the adjacent ${UC}_{12{}^{\circ}}$ cell, the corresponding efficiencies decrease from 93.4%, 91.5%, and 89.9% to 73.5%, 71.0%, and 83.7%, respectively, as shown in Figure 4d. The corresponding stitching errors are 37.8% for the interface‐adjacent ${UC}_{8{}^{\circ}}$ cell and 28.9% for the interface‐adjacent ${UC}_{12{}^{\circ}}$ cell. These results demonstrate that independently optimized angle‐specific color routers can experience substantial local performance degradation once physically stitched together, even for neighboring angular zones separated by only 4°. A similar interface‐localized degradation trend is observed for the θ=18° case, as shown in Supporting Information S1: Figure S10. In a full sensor comprising many such interfaces, the repeated occurrence of these local mismatches would be expected to produce spatially nonuniform sensitivity and severe color distortion. The 2D study thus identifies stitching error as a critical limitation of independently optimized angle‐specific color routers and directly motivates the OSS‐constrained framework introduced in Section 2.

### 3.2 Three‐Dimensional Volumetric, OSS‐Constrained Color Routers Toward Full Sensor‐Plane Coverage

We employ the OSS framework to construct a 3D freeform color‐router library that provides a scalable route toward full sensor‐plane coverage. Specifically, our approach proceeds in three key stages. First, we generate the unit‐cell library via a sequential optimization strategy that yields smoothly evolving structures across the angular grid. Second, we evaluate the isolated optical performance and structural continuity of these individual unit cells. Third, we quantify the stitching error and overall performance degradation by extracting the spectral responses of stitched supercells across four representative multi‐zone boundary configurations.

To rigorously assess stitching‐induced degradation, we focus on the most stringent multi‐zone interfaces: four‐zone junctions where the incident angles in both ϕ and θ change simultaneously. To model the local stitched geometry under such conditions, we construct a 6×6 supercell assembled from the four adjacent angular zones, with each zone represented by nine copies of its corresponding unit cell. As illustrated in Figure 5a and (d) (see also Supporting Information S1: Figure S11), we classify these junctions into *Q‐type* and *H‐type* boundaries, which correspond to representative four‐zone intersections located near azimuthal angles of ϕ≈0° and ϕ≈45°, respectively. Although this supercell is smaller than the 2D domains used in Section 3.1, Supporting Information S1: Note S6 confirms, through both optical‐efficiency spectra and field‐profile convergence analyses, that it is sufficiently large to capture the relevant local stitching behavior.

**FIGURE 5:**
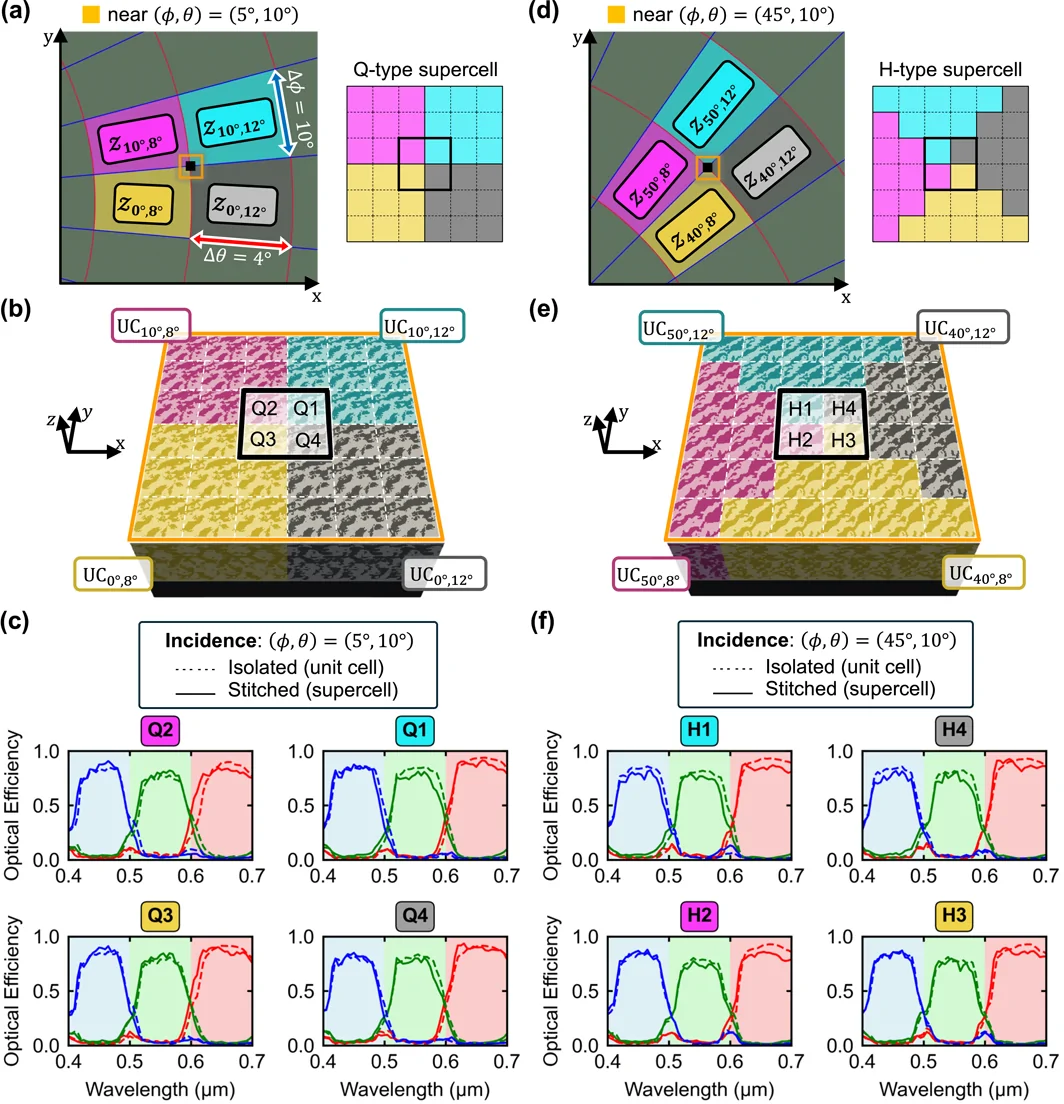
Representative stitched‐supercell validation of the OSS‐constrained 3D angle‐specific color‐router library on Grid 1 with (Δϕ,Δθ)=(10°,4°) and τ=0.90. Two stringent four‐zone junctions at θ=10° are shown: (a–c) Q‐type near (ϕ,θ)=(5°,10°) and (d–f) H‐type near (ϕ,θ)=(45°,10°). A *Q‐type* junction denotes the quadrant‐like four‐zone meeting that arises near ϕ≈0°, whereas an *H‐type* junction denotes the staggered, step‐like meeting that arises near ϕ≈45°. In each case, the panels show the local angular partition, the corresponding 6×6 stitched supercell formed from the four neighboring unit cells, and optical‐efficiency spectra of the boundary‐adjacent cells (Q1–Q4 or H1–H4), comparing isolated unit‐cell responses (dashed) with stitched‐supercell responses (solid). The close spectral agreement across the three color channels confirms minimal stitching‐induced degradation even at four‐zone junctions.

We first adopt *Grid 1* with (Δϕ,Δθ)=(10°,4°) and impose an OSS threshold of τ=0.90. The choice of τ reflects a trade‐off between preserving isolated optical efficiency and enforcing structural similarity between neighboring angular zones; the threshold dependence is examined in Supporting Information S1: Note S4.6. The unit‐cell library is then constructed sequentially, beginning from the innermost unit cell and progressively expanding outward across the angular grid (See Supporting Information S1: Note S4 and Figure S15 for details). This sequential construction yields a library that evolves smoothly; neighboring cells achieve structural similarities of 90%–92% primarily through sparse, localized modifications.

Importantly, this structural continuity is achieved without compromising optical performance. Under isolated Bloch boundary conditions, the Grid 1 library maintains strong optical responses. As shown in Supporting Information S1: Figure S18, within these ranges, the average optical efficiency reaches 85.1%, whereas the average optical cross talk remains as low as 2.00%. These values are comparable to those obtained from an independently optimized unit‐cell library on the same Grid 1, which achieves an average optical efficiency of 90.5% and an average optical cross talk of 1.60% (See Supporting Information S1: Note S4 and Figure S13 for details). Taken together, these results demonstrate that the OSS framework effectively enforces structural continuity while preserving the design flexibility required for angle‐specific adaptation.

We next examine four specific incident‐angle configurations: two Q‐type boundaries centered at (ϕ,θ)=(5°,10°) and (5°,18°), and two H‐type boundaries at (45°,10°) and (45°,18°). Figure 5a,d illustrates the local angular partitions for the representative θ=10° cases, whereas the corresponding results for the θ=18° cases are provided in Supporting Information S1: Figure S19. We assess the stitching errors by comparing the optical‐efficiency spectra of the boundary‐adjacent cells (denoted as Q1–Q4 and H1–H4) within the stitched supercells against their isolated‐unit‐cell references. As shown in Figure 5c,f, the stitched spectra closely match the isolated responses across all three color channels, confirming that stitching introduces only minor perturbations even at these stringent four‐zone junctions. Quantitatively, the average stitching error across the four tested Grid 1 configurations is 11.9%, representing a 2.1× reduction compared to the independently optimized library (24.6%; see Supporting Information S1: Figure S14). Relative to the isolated responses, the OSS‐constrained stitched array exhibits merely a 3.40% decrease in optical efficiency and a 1.06% increase in cross talk. These results demonstrate that sequential OSS‐constrained library construction provides a practical strategy for realizing 3D angle‐specific color‐router libraries that can be extended toward full sensor‐plane coverage while maintaining high optical performance and mitigating stitching errors.

Although Grid 1 confirms the validity of the OSS constraint, the residual stitching errors suggest a natural direction for further refinement. By adopting a finer angular grid and imposing stricter structural similarity, we may systematically suppress these perturbations. We thus construct a refined unit‐cell library, denoted as Grid 2, utilizing reduced step sizes of (Δϕ,Δθ)=(5°,2°) and a more stringent OSS threshold of τ=0.95.

Angular discretization is a critical design parameter that governs the trade‐off between library complexity and inter‐cell continuity at stitched interfaces. For a fixed angular coverage, reducing the step sizes from (Δϕ,Δθ)=(10°,4°) to (5°,2°) increases the number of angular zones, and thus the number of distinct unit cells, by approximately a factor of four. As a result, finer discretization leads to a larger library and more stitching interfaces. However, it also ensures that adjacent zones correspond to more similar incident‐wave conditions and that each unit cell covers a narrower angular range, making it easier to satisfy a stringent OSS constraint without significantly degrading isolated performance.

In practice, prior to stitching, the OSS‐constrained Grid 2 library exhibits strong optical performance while maintaining at least 95% OSS between all adjacent unit cells. As detailed in Supporting Information S1: Figure S22, the average optical efficiencies in the red, green, and blue channels are 91.15%, 80.87%, and 83.93%, respectively, whereas the corresponding optical crosstalk values are 1.26%, 2.72%, and 1.81%. These metrics remain comparable to those of Grid 1, verifying that tighter angular sampling combined with stricter OSS enforcement provides a highly continuous library without compromising design capability.

Applying the same supercell evaluation method to Grid 2, we examine four corresponding multi‐zone junctions defined by the finer discretization: two Q‐type boundaries centered at (ϕ,θ)=(2.5°,9°) and (2.5°,19°), and two H‐type boundaries at (42.5°,9°) and (42.5°,19°). Figure 6 illustrates the representative θ=19° cases, whereas the corresponding θ=9° results are provided in Supporting Information S1: Figure S23. In these critical four‐zone junctions, the optical‐efficiency spectra extracted from the boundary‐adjacent cells in the stitched supercells exhibit near‐perfect agreement with their isolated counterparts, as shown in Figure 6c,f. Consistently, the stitching error decreases to 5.99%, which is approximately half that of the OSS‐constrained Grid 1 library (11.9%); for prior reference, the independently optimized Grid 1 library yielded 24.6%.

**FIGURE 6:**
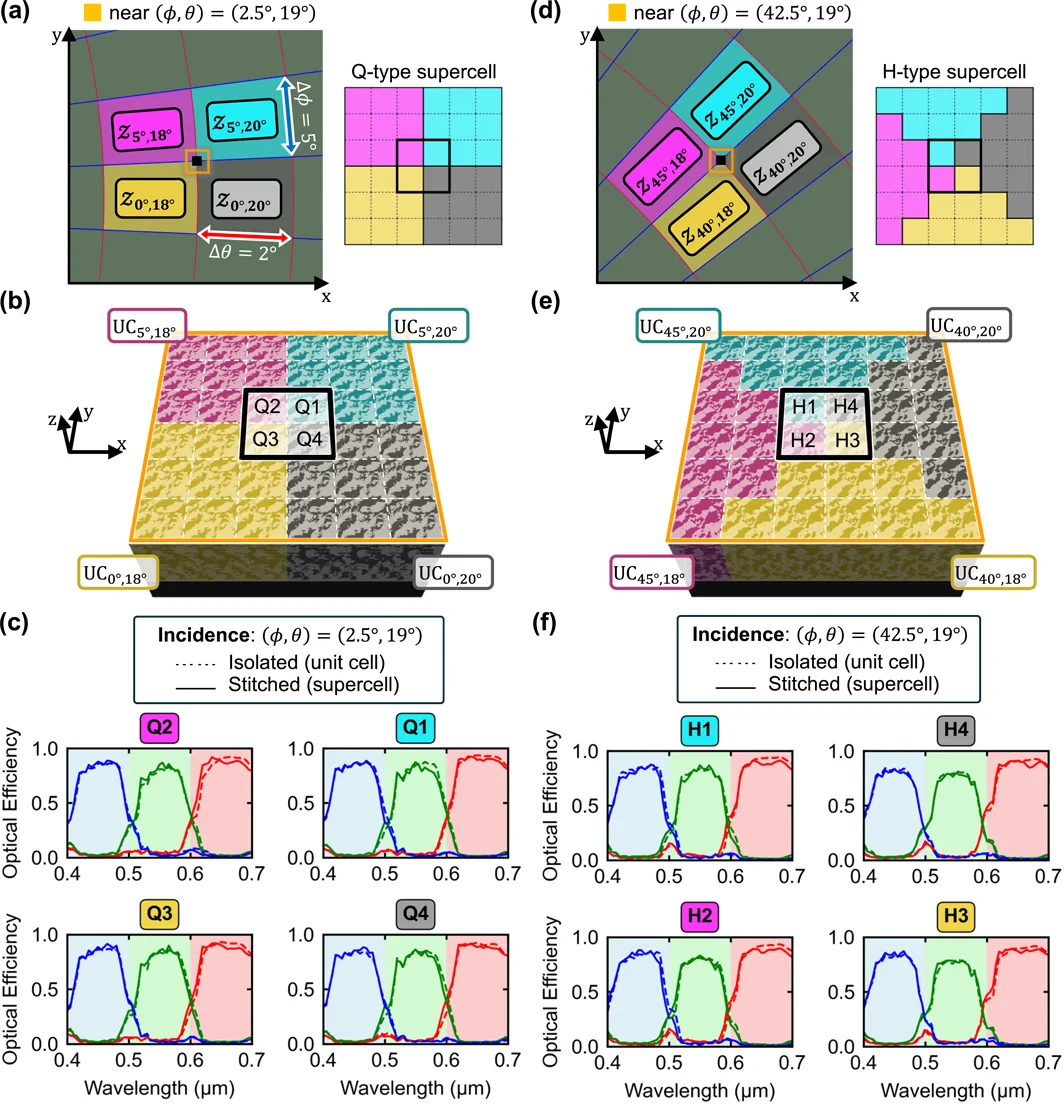
Representative stitched‐supercell validation of the OSS‐constrained 3D angle‐specific color‐router library on Grid 2 with (Δϕ,Δθ)=(5°,2°) and τ=0.95. Two representative four‐zone junctions at fixed θ=19° are shown: (a–c) Q‐type near (ϕ,θ)=(2.5°,19°) and (d–f) H‐type near (ϕ,θ)=(42.5°,19°). In each case, the panels show the local angular partition, the corresponding 6×6 stitched supercell formed from the four neighboring unit cells, and optical‐efficiency spectra of the boundary‐adjacent cells (Q1–Q4 or H1–H4), comparing isolated unit‐cell responses under Bloch boundary conditions (dashed) with stitched‐supercell responses (solid). In both cases, the stitched‐supercell spectra closely track the isolated unit‐cell references across the red, green, and blue channels, confirming that the finer angular sampling and stricter OSS enforcement of Grid 2 effectively suppress stitching‐induced perturbations.

These results indicate that once discontinuities between neighboring angular zones are sufficiently suppressed, isolated unit‐cell analysis becomes a significantly more reliable predictor of stitched‐array behavior. More broadly, these findings further point to the possibility that, with sufficient computational resources, a fully continuous spatially graded unit‐cell distribution could be leveraged to realize color routers operating over ultra‐wide angular ranges.

The Grid 2 core library spans $\phi_{0}=0{}^{\circ},5{}^{\circ},\text{…},45{}^{\circ}$ and $\theta_{0}=2{}^{\circ},4{}^{\circ},\text{…},20{}^{\circ}$, yielding 100 optimized unit cells, together with the innermost cell ${UC}_{0{}^{\circ}}$. Extending the library to explicit 360° azimuthal coverage for every polar zone would increase the number of unit cells proportionally and incur substantial computational overhead. Accordingly, in the present work, the high‐polar‐angle designs are restricted to the sector $0{}^{\circ}\leq \phi_{0}\leq 45{}^{\circ}$. To examine whether the OSS‐based construction can nevertheless be closed over the full azimuthal circle, we additionally construct circular branches for the four smallest polar zones, $\theta_{0}\in \left\{2{}^{\circ},4{}^{\circ},6{}^{\circ},8{}^{\circ}\right\}$, by explicitly optimizing unit cells for $\phi_{0}=0{}^{\circ},5{}^{\circ},\text{…},355{}^{\circ}$. This extension adds 248 unit cells, bringing the explicit angular library to 348 cells in total.

The computational cost associated with this library construction is quantified in Supporting Information S1: Note S4.5. In the present CPU‐only implementation, each unit‐cell optimization required approximately 3.5 h on a 128‐core workstation; thus, the practical library size should be selected according to the desired trade‐off among angular resolution, stitching robustness, and computational budget.

As illustrated in Supporting Information S1: Figure S25, the circular branch is generated by continuing the sequential OSS‐constrained optimization from ${UC}_{45{}^{\circ},\theta_{0}}$ in the direction of increasing $\phi_{0}$ up to ${UC}_{355{}^{\circ},\theta_{0}}$. At the wrap‐around seam, the terminal cell is simultaneously adjacent to the two azimuthal neighbors ${UC}_{350{}^{\circ},\theta_{0}}$ and ${UC}_{0{}^{\circ},\theta_{0}}$, as well as to the corresponding lower‐θ reference cell, and therefore requires simultaneous OSS matching to three reference neighbors. The resulting closure cells satisfy OSS≥95% with respect to all reference neighbors. Their isolated optical responses remain clearly separated over the assigned angular sets, as shown in Supporting Information S1: Figure S26. Moreover, stitched‐supercell evaluation at the closure boundary (ϕ=357.5°) yields an average stitching error of 4.95%, which is comparable to the values obtained at interior boundaries (See Supporting Information S1: Note S4 and Figure S27 for details). These results demonstrate that the OSS framework can accommodate full azimuthal wrap‐around without loss of stitching robustness in the low‐polar‐angle regime considered here.

The OSS‐based framework reveals a broader practical advantage of the angle‐specific approach. Because each unit cell is required to operate only within a limited angular range, rather than across the full range of incident angles in a CIS, the structural design burden associated with maintaining high optical performance is significantly reduced. This relaxation suggests that the same framework can be implemented using architectures with fewer design degrees of freedom, such as multilayer stacks, rather than being limited to fully freeform geometries. Moreover, whereas conventional CIS architectures often accommodate oblique incidence through angle‐tailored optical stacks, such as laterally shifted microlens and color‐filter configurations, the present framework achieves robust oblique‐incidence color routing through spatially varying nanophotonic design itself, without requiring such geometrical adjustments. These observations straightforwardly motivate the multilayer implementation discussed in Section 3.3.

### 3.3 Five‐Layer Extension of the OSS‐Constrained Design

Motivated by the relaxed structural design burden within narrow angular zones, we investigate whether strong stitching robustness can be maintained under substantially reduced geometric degrees of freedom. We thus extend the OSS‐constrained design to a *five‐layer* architecture.

The design region retains its overall volume (1.2μm×1.2μm×2.0μm) but is now restricted to five stacked layers, each 0.4μm thick, with a minimum feature size of 60nm. We adopt the same Grid 2 angular discretization with (Δϕ,Δθ)=(5°,2°) and OSS threshold (τ=0.95) used for the fully freeform library in Section 3.2. Despite the highly constrained design space, the resulting five‐layer library successfully supports clear angle‐specific color routing. Prior to stitching, the average isolated optical efficiencies for the red, green, and blue pixels reach 83.6%, 60.0%, and 69.8%, respectively, with corresponding crosstalk values of 2.66%, 9.34%, and 6.72% (See Supporting Information S1: Note S5 and Figure S30 for details).

We evaluate the stitching robustness of the five‐layer library using the same four‐junction configurations described in Section 3.2. Representative results for θ=19° are shown in Figure 7, whereas the θ=9° cases are provided in Supporting Information S1: Figure S31. The optical‐efficiency spectra of the boundary‐adjacent cells in the stitched supercells closely track their isolated references. The stitched structures exhibit an average efficiency reduction of merely 1.81% and a cross talk increase of only 0.19%. The average stitching error is 5.65%, which remains comparable to that of the freeform Grid 2 library (5.99%) reported in Section 3.2, despite the lower isolated optical performance. These results indicate that the OSS‐based angle‐specific design strategy can be effectively extended to reduced‐complexity multilayer stacks, thereby enhancing its practical applicability to manufacturable color‐router libraries.

**FIGURE 7:**
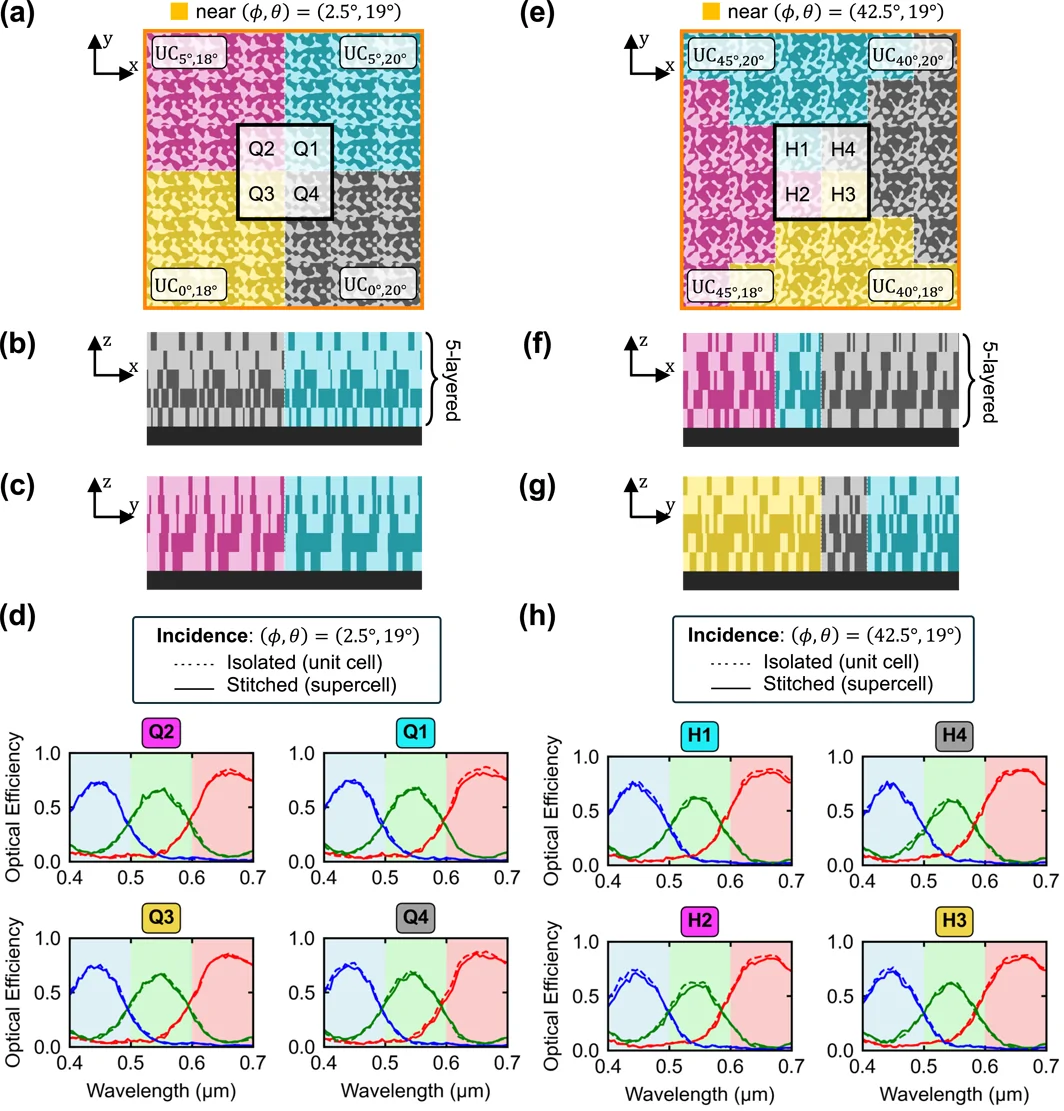
Representative θ=19° stitched‐boundary validation of the five‐layer OSS‐constrained library on Grid 2 [(Δϕ,Δθ)=(5°,2°), τ=0.95]. Two representative boundaries are shown: (a–d) Q‐type near (ϕ,θ)=(2.5°,19°) and (e–h) H‐type near (ϕ,θ)=(42.5°,19°). In each case, the panels show the middle‐layer layout of the corresponding 6×6 stitched supercell, the associated xz and yz cross‐sections, and optical‐efficiency spectra of the boundary‐adjacent cells (Q1–Q4 or H1–H4), comparing isolated unit‐cell responses under Bloch boundary conditions (dashed) with stitched‐supercell responses (solid). In both representative cases, the stitched spectra remain close to the isolated references, confirming that the five‐layer OSS‐constrained design largely preserves the intended angle‐specific spectral routing after stitching.

## 4 Conclusion

We presented an angle‐specific inverse‐design framework for nanophotonic color routers that addresses a key barrier to practical deployment in CISs: stitching‐induced degradation between adjacent angular zones. Instead of seeking a single design that performs well over a broad angular range, we discretize the incident‐angle space into local zones and assign each zone a dedicated unit‐cell color router. To ensure compatibility after assembly, we introduced an *optical structural similarity* (OSS) constraint that enforces controlled continuity in permittivity during topology optimization, without requiring additional full‐wave simulations.

Our results show that stitching compatibility is essential for practical angle‐specific color‐router libraries. In 2D, neighboring unit cells optimized independently suffered severe degradation after stitching despite strong isolated performance. In 3D freeform designs, the OSS constraint substantially improved agreement between isolated and stitched responses while maintaining high routing performance. The average stitching error decreased from 24.6% for the independently optimized library to 11.9% on Grid 1, and further to 5.99% on Grid 2 with finer angular sampling and a stricter similarity threshold. The approach also extended to a reduced‐complexity five‐layer architecture, which achieved a comparable stitching error of 5.65%, although its isolated optical performance was lower than that of the fully freeform designs.

These results indicate that the primary challenge in angle‐specific color routing lies in achieving both high performance of individual unit cells and compatibility across stitched angular zones. By incorporating structural continuity into the local optimization process, the OSS framework makes isolated unit‐cell analysis a more reliable predictor of stitched‐array behavior and provides a practical pathway toward scalable, full‐sensor‐plane color‐router libraries. We note, however, that the present explicit validation does not constitute a complete all‐angle library over the entire sensor plane. In this work, high‐polar‐angle stitching robustness was validated over representative azimuthal sectors up to $\theta_{0}=20{}^{\circ}$, whereas full 360° azimuthal wrap‐around closure was explicitly demonstrated for the low‐polar‐angle branches $\theta_{0}\leq 8{}^{\circ}$. A clear trade‐off therefore remains: Finer angular discretization and broader azimuthal coverage enhance sensor‐plane generality but increase library size and optimization cost.

We also emphasize that, although the present libraries were optimized under a single 45° linearly polarized excitation, the OSS regularizer itself is defined only by the permittivity distribution and is therefore independent of the optical excitation or polarization state. Supporting Information S1: Note S2.4 further shows that representative unit cells preserve wavelength‐selective routing under separate TE‐ and TM‐polarized oblique incidence and that polarization‐insensitive, OSS‐constrained libraries can be straightforwardly constructed by including both TE and TM responses in the optimization objective. These results suggest that polarization robustness can be incorporated together with OSS‐based stitching‐error suppression within the same design framework.

Future work should complete the full 360° azimuthal extension at larger polar angles, incorporate fabrication tolerances and realistic material losses more explicitly, exploit parallel and GPU‐accelerated electromagnetic solvers for larger angular libraries, and provide experimental validation. More broadly, the OSS framework suggests a general design principle for large‐area metasurfaces and meta‐optic systems that require spatially varying functionality while maintaining robustness against inter‐element discontinuities.

## Author Contributions

**Donghyun Kim:** methodology, conceptualization, software, data curation, investigation, validation, formal analysis, visualization, writing – original draft, writing – review and editing. **Junseo Han:** software, data curation, formal analysis. **Seunghyun Lee:** software, data curation, formal analysis. **Min Seok Jang:** supervision, writing – review and editing. **Haejun Chung:** supervision, funding acquisition, visualization, project administration, resources, writing – review and editing, writing – original draft.

## Conflicts of Interest

The authors declare no conflicts of interest.

## Supporting information


Supporting Information S1

## Data Availability

The simulation data and optimization code will be made available upon reasonable request.
