# Supplementary material for: Suppressing Stitching Errors in Full‐Sensor‐Plane Color Routers via Optical Structural Similarity
Source: Nanophotonics. 2026 Jul 20;15(14):e70220. doi: 10.1002/nap2.70220 (PMC13642622; doi:10.1002/nap2.70220)
Supplement: Supplementary file 1 [file NAP2-15-e70220-s001.pdf]

Supporting Information: Suppressing Stitching Errors in Full-Sensor-Plane Color Routers via Optical Structural Similarity

Donghyun Kim[1]**, Junseo Han**[2]**, Seunghyun Lee**[1]**, Min Seok Jang**[4]**, Haejun Chung**[1,2,3,*]
[1]Department of Electronic Engineering, Hanyang University, Seoul 04763, Republic of Korea
[2]Department of Artificial Intelligence Semiconductor Engineering, Hanyang University, Seoul 04763, Republic of Korea
[3]Department of Artificial Intelligence, Hanyang University, Seoul 04763, Republic of Korea
[4]School of Electrical Engineering, Korea Advanced Institute of Science and Technology, Daejeon 34141, Republic of Korea

This supplementary document provides additional analysis, derivations, optimization details, isolated performance of each unit-cell library, and stitched-supercell validation results that support the main manuscript.

Note S1. Angular sensitivity of a color router optimized exclusively for normal incidence

To assess how rapidly performance degrades when the color router is optimized only for normal incidence, we evaluated the resulting device under oblique illumination while sweeping the polar angle from $-10°$ to $10°$. As shown in Figure S1, the red, green, and blue optical efficiencies all exhibit a pronounced maximum at $\theta = 0°$, indicating that the device performs best at the design angle.

As $|\theta|$ increases, the efficiencies of all three channels decrease rapidly. The average optical efficiency,

$$\bar{\eta} = \frac{\eta_{\mathrm{R}} + \eta_{\mathrm{G}} + \eta_{\mathrm{B}}}{3},$$

shows a narrow on-axis peak and falls to approximately one-half of its maximum value by $|\theta| \approx 6°$. The angular response is also nearly symmetric with respect to positive and negative tilt, indicating that the dominant degradation is governed primarily by the magnitude of the deviation from the design angle rather than by its sign. These results directly motivate the angle-specific color-routing strategy developed in the main text.

Note S2. Optimization Implementation and Approximation Validation

S2.1. Adjoint-based topology optimization

The 3D freeform color router consists of a $1.2 \times 1.2 \times 2.0\ \mu\mathrm{m}^3$ design region placed above a 2×2 Bayer-pattern photodetector array. Each photodetector has a lateral footprint of $0.48 \times 0.48\ \mu\mathrm{m}^2$, and adjacent photodetectors are separated by $0.12\ \mu\mathrm{m}$-wide deep-trench-isolation regions, resulting in a unit-cell pitch of $1.2\ \mu\mathrm{m}$. The design region is discretized into $(20\ \mathrm{nm})^3$ cubic voxels, corresponding to approximately 3.6×10^5 degrees of freedom. In this work, the color-router designs are represented by two lossless dielectric materials, silica and silicon nitride, with dielectric constants $\varepsilon_{\mathrm{low}} = 2.10$ and $\varepsilon_{\mathrm{high}} = 4.41$, respectively.

The design region is parameterized by a continuous variable $\rho(\mathbf{x}) \in [0, 1]$, where $\mathbf{x}$ denotes the spatial coordinate. To impose a minimum feature size, the raw design variable is first smoothed by a conic filter:[1]

$$\tilde{\rho}(\mathbf{x}) = \omega(\mathbf{x}) * \rho(\mathbf{x}), \tag{S1}$$

where $\omega(\mathbf{x})$ is the filter kernel and $*$ denotes convolution. The filtered variable is then projected toward binary values using a hyperbolic-tangent mapping:[2]

$$\bar{\rho}(\mathbf{x}) = \frac{\tanh(\beta\eta) + \tanh\big(\beta(\tilde{\rho}(\mathbf{x}) - \eta)\big)}{\tanh(\beta\eta) + \tanh\big(\beta(1 - \eta)\big)}, \tag{S2}$$

where β is the projection sharpness and $\eta = 0.5$ is the threshold. During optimization, β is gradually increased so that the final design approaches a binary distribution. The permittivity used in the electromagnetic simulation is obtained by linear interpolation between the two design

materials:

$$\varepsilon(\mathbf{x}) = \varepsilon_{\text{low}} + \bar{\rho}(\mathbf{x})\big(\varepsilon_{\text{high}} - \varepsilon_{\text{low}}\big). \tag{S3}$$

At convergence, this filtering/projection schedule yields binarized final silica/silicon-nitride structures.

For a sampled incident angle pair (ϕ_m, θ_m), the optical figure of merit used in the main text is

$$\mathcal{F}_{\text{OE}} = \sum_{i\in\{\text{R,G,B}\}} \text{OE}_i - \gamma\big(\text{OE}_{\text{G}_1} - \text{OE}_{\text{G}_2}\big)^2, \tag{S4}$$

where each OE_i is the band-averaged optical efficiency of the corresponding color channel and the penalty term enforces balanced power delivery between the two green pixels. Once the objective is specified, the adjoint source is defined from the functional derivative of $\mathcal{F}_{\text{OE}}$ with respect to the field on the measurement plane:

$$\mathbf{J}_i^{\text{adj}}(\mathbf{x}') = -j\omega \frac{\partial \mathcal{F}_{\text{OE},i}}{\partial \mathbf{E}(\mathbf{x}')}, \tag{S5}$$

where $\mathbf{x}'$ denotes the measurement-plane coordinate and $\mathcal{F}_{\text{OE},i}$ is the channel-wise contribution to the objective.

For each color channel, the forward simulation gives the field $\mathbf{E}_i^{\text{fwd}}(\mathbf{x};\phi_m,\theta_m)$ inside the design region, and the corresponding adjoint simulation gives $\mathbf{E}_i^{\text{adj}}(\mathbf{x};\phi_m,\theta_m)$. The channel-wise permittivity gradient is then obtained from the forward–adjoint field overlap:

$$\frac{\partial \mathcal{F}_{\text{OE},i}}{\partial \varepsilon(\mathbf{x})} = \text{Re}\Big[\mathbf{E}_i^{\text{fwd}}(\mathbf{x};\phi_m,\theta_m)\cdot \mathbf{E}_i^{\text{adj}}(\mathbf{x};\phi_m,\theta_m)\Big], \tag{S6}$$

and the total optical gradient is the sum over the three color channels:

$$\frac{\partial \mathcal{F}_{\mathrm{OE}}}{\partial \varepsilon(\mathbf{x})} = \sum_{i \in \{\mathrm{R,G,B}\}} \frac{\partial \mathcal{F}_{\mathrm{OE},i}}{\partial \varepsilon(\mathbf{x})}. \tag{S7}$$

The gradient with respect to the underlying design variable $\rho(\mathbf{x})$ is obtained by backpropagating the permittivity gradient through the material interpolation, hyperbolic-tangent projection, and conic-filter operations via the chain rule. When OSS regularization is included, its analytical gradient is added to $\partial \mathcal{F}_{\mathrm{OE}}/\partial \varepsilon(\mathbf{x})$ before this backpropagation. The resulting gradient is then used to update $\rho(\mathbf{x})$ with the Adam optimizer.[3] For each selected incident angle, one forward and one adjoint simulation are carried out independently for each of the three color channels, so each optimization iteration involves six full-wave simulations in total.

S2.2. Effective incident-angle deviation under Bloch boundary conditions

Under lateral Bloch boundary conditions, the in-plane wavevector is fixed during a given simulation. For a target plane wave specified by azimuthal angle ϕ_0, polar angle θ_0, and reference wavelength λ_0, the imposed in-plane wavevector is

$$\mathbf{k}_{\parallel}^{(0)} = \frac{2\pi}{\lambda_0} \sin\theta_0 \begin{bmatrix} \cos\phi_0 \\ \sin\phi_0 \end{bmatrix}. \tag{S8}$$

If the same wavevector is reused at another wavelength λ, the direction of $\mathbf{k}_{\parallel}$ is unchanged, so the effective azimuthal angle remains $\phi_{\mathrm{eff}} = \phi_0$, whereas the effective polar angle becomes wavelength dependent:

$$\theta_{\mathrm{eff}}(\lambda) = \sin^{-1}\left(\frac{|\mathbf{k}_{\parallel}^{(0)}|}{2\pi/\lambda}\right) = \sin^{-1}\left(\frac{\lambda}{\lambda_0}\sin\theta_0\right). \tag{S9}$$

The resulting polar-angle deviation is therefore

$$\Delta\theta(\lambda;\lambda_0,\theta_0) = \theta_{\text{eff}}(\lambda) - \theta_0. \tag{S10}$$

Equation (S10) shows that the mismatch vanishes at normal incidence and increases with both the spectral detuning $|\lambda - \lambda_0|$ and the target polar angle θ_0. Table S1 first reports the deviation obtained when a single broadband source centered at 550 nm is used over the visible range. In that case, the mismatch becomes substantial near the spectral edges and reaches $\pm 5.59^\circ$ at $\theta_0 = 22^\circ$.

To reduce this mismatch inside the optimization loop, we divide the visible band into three sub-bands and use separate simulations centered at $\lambda_0 = 650$ nm (red), 550 nm (green), and 450 nm (blue). Table S2 summarizes the resulting deviations at wavelengths ± 30 nm from each band center. For $\theta_0 \leq 20^\circ$, the maximum deviation remains within approximately $\pm 1.4^\circ$, and even at $\theta_0 = 22^\circ$ it is limited to about $\pm 1.55^\circ$. This comparison justifies the three-source approximation used during optimization. We emphasize that this approximation is used only within the optimization loop; all final device performances reported in the main text are evaluated using wavelength-resolved simulations in which $\mathbf{k}_\parallel$ is recalculated for each (λ, ϕ, θ).

S2.3. Fixed-azimuth sampling approximation

The optimization loop in the main text fixes the sampled azimuth at $\phi_m = \phi_0$ and sweeps only the polar angle θ_m within the angular zone $\mathcal{Z}_{\phi_0,\theta_0}$. To assess the validity of this approximation for the final 3D freeform library, we evaluated the azimuthal-angle dependence of representative unit cells using post-optimization wavelength-resolved simulations. We chose the same high-polar-angle representative cells used in the isolated-spectrum validation of Grid 1 and Grid 2, namely $\mathrm{UC}_{0^\circ,20^\circ}$

and $\mathrm{UC}_{50^\circ,20^\circ}$ from Grid 1 and $\mathrm{UC}_{0^\circ,20^\circ}$ and $\mathrm{UC}_{45^\circ,20^\circ}$ from Grid 2. All simulations in this note were performed at the zone-center polar angle $\theta = \theta_0 = 20^\circ$, where any residual angular sensitivity is expected to be most visible.

Figure S2 shows the optical-efficiency spectra of these four unit cells as the azimuth is varied about the zone center. For Grid 1, we sweep $\phi = \phi_0 + \delta\phi$ with $\delta\phi \in \{-5^\circ, -4^\circ, \ldots, 5^\circ\}$, consistent with the $\Delta\phi = 10^\circ$ angular discretization. For Grid 2, we use $\delta\phi \in \{-2.5^\circ, -2^\circ, \ldots, 2.5^\circ\}$, corresponding to representative azimuthal offsets within the finer $\Delta\phi = 5^\circ$ discretization. In all four cases, the red-, green-, and blue-channel spectra remain largely unchanged as ϕ varies around ϕ_0.

The overlap is especially tight for the Grid 2 examples, consistent with the narrower azimuthal span of the finer angular sampling. These results support the reduced angular sampling strategy adopted in the optimization loop: within a given angular zone, the device response is substantially more sensitive to variations in θ than to the comparatively small variations in ϕ. Fixing $\phi_m = \phi_0$ therefore reduces the computational cost of each optimization sweep without materially changing the target angle-robust behavior of the final unit-cell library.

S2.4. Polarization study

In natural-scene imaging, light incident on a CIS is generally unpolarized. A rigorous assessment of polarization-independent operation would thus require separate simulations for TE- and TM-polarized illumination and incoherent averaging of the resulting responses, which approximately doubles the computational cost of each optimization iteration. To improve efficiency, we use a single excitation condition throughout the main text: a plane wave linearly polarized at 45° with respect to the TE/TM basis. While this choice contains equal TE and TM amplitudes, it is a coher-

ent superposition and therefore not strictly equivalent to unpolarized illumination. The reported results should therefore be interpreted as responses to 45° linearly polarized incidence.

To assess the role of polarization, we examine the color-router response at three levels. First, representative unit cells from the main-text libraries are re-evaluated under separate TE- and TM-polarized illumination to quantify the polarization dependence of the designs used in the main text. Second, an auxiliary dual-polarization optimization is performed for the innermost angular zone to examine whether high-efficiency color routing can be obtained when TE and TM responses are explicitly included in the objective. Third, an additional polarization-insensitive, OSS-constrained Grid 2 library subset is constructed and evaluated at both the isolated-unit-cell and stitched-supercell levels, thereby testing whether polarization-resolved operation can be combined with the OSS-based stitching-error suppression strategy.

To examine the polarization dependence of the unit-cell libraries used in the main text, we performed polarization-resolved simulations for representative unit cells under separate TE- and TM-polarized illumination. Figure S3 summarizes the optical-efficiency spectra of five representative unit cells from the freeform Grid 2 and five-layered Grid 2 libraries. In addition, the freeform Grid 1 library was evaluated quantitatively under the same polarization-resolved conditions. For each architecture, the responses were analyzed separately for TE- and TM-polarized incidence, and the red, green, and blue target spectral bands are shaded in Fig. S3 for clarity.

For the freeform libraries, the representative unit cells preserve the intended color-routing behavior under both TE- and TM-polarized illumination. For the freeform Grid 2 library, this behavior is shown in Figs. S3(a) and S3(b): the red-targeting designs exhibit high optical efficiency in the red spectral band, while the green- and blue-targeting designs maintain their corresponding band-selective responses. Quantitatively, the average optical efficiency of the examined freeform

Grid 1 unit cells decreases from 85.1% under the $45°$ linearly polarized excitation used in the main text to 70.0% and 68.8% under TE- and TM-polarized illumination, respectively. Similarly, the average optical efficiency of the examined freeform Grid 2 unit cells decreases from 85.3% to 69.9% and 68.0% under TE- and TM-polarized illumination, respectively. The corresponding TE–TM efficiency differences are 1.2 and 1.9 percentage points for Grid 1 and Grid 2, respectively, indicating that the two polarization states lead to similar averaged efficiencies for the representative sets, although the absolute efficiency is reduced under the polarization-resolved oblique-incidence conditions.

A similar trend is observed for the five-layered Grid 2 library. As shown in Figs. S3(c) and S3(d), the five-layered unit cells also retain their wavelength-selective routing characteristics for both TE and TM incidence. The average optical efficiency decreases from 71.1% under the $45°$ linearly polarized excitation to 59.4% and 59.5% under TE- and TM-polarized illumination, respectively. The nearly identical TE and TM efficiencies indicate that the five-layered designs exhibit only a small polarization imbalance for the representative unit cells considered here.

These results clarify the role of polarization in the main-text libraries. The main-text libraries were primarily constructed to satisfy the OSS constraint and suppress stitching-error-induced performance degradation, rather than to explicitly optimize polarization-insensitive operation. Consequently, a reduction in absolute optical efficiency is observed when the designs are evaluated under separate TE- and TM-polarized oblique incidence. Nevertheless, the representative unit cells maintain their target spectral selectivity, and the averaged TE–TM efficiency differences remain small. These observations motivate the polarization-explicit optimizations described below.

As a first step toward polarization-explicit design, a separate auxiliary optimization was performed for the innermost angular zone by explicitly including both TE- and TM-polarized illu-

mination cases in the objective. Here, UC_{0° refers to the newly optimized dual-polarization unit cell for the innermost angular zone, and should be distinguished from the UC_{0° design used in the main-text library.

The auxiliary UC_{0° was assigned to the polar-angle range $\theta \in [-2^\circ, 2^\circ]$. In this optimization, the TE and TM responses were weighted equally so that the structure was required to maintain high color-routing performance for both orthogonal polarization states across the full assigned angular range. Since this design corresponds to the innermost angular zone and is optimized without OSS constraints from neighboring cells, it provides a direct assessment of the intrinsic polarization dependence of the angle-specific color-routing mechanism.

Figure S4 summarizes the performance of the resulting design. Panels (a) and (b) show the optical-efficiency spectra under TE- and TM-polarized illumination, respectively, evaluated at the five sampled incident angles $\theta = -2^\circ, -1^\circ, 0^\circ, 1^\circ$, and 2°. For both polarization states, the red, green, and blue responses remain clearly separated and retain high efficiency in their respective target channels over the entire assigned angular range. Moreover, within each color channel, the spectra obtained at the five sampled angles are closely grouped, indicating that the device performance varies only weakly across $\theta \in [-2^\circ, 2^\circ]$. Panel (c) further shows the field-intensity distributions at the photodetector-array plane under normal incidence for TE- and TM-polarized illumination. In both cases, the optical energy is routed predominantly to the intended photodetector region: red light to the red pixel, green light to the two green pixels, and blue light to the blue pixel. The close similarity of the detector-plane intensity profiles for the two polarization states confirms that the underlying routing behavior is preserved under both TE and TM excitation.

Taken together, these results show that angle-specific color routing can also be achieved using a design obtained from a full dual-polarization optimization. Thus, although the main text

focuses on $45°$ linearly polarized incidence for computational efficiency, the auxiliary optimization demonstrates that polarization-robust color-router designs can be obtained when both TE- and TM-polarized illumination are explicitly considered.

While the auxiliary single-zone design confirms the intrinsic feasibility of dual-polarization color routing, it does not address whether such polarization-explicit optimization remains compatible with the OSS-constrained library construction used to suppress stitching errors. To examine this point, the dual-polarization treatment was extended to the OSS-constrained library level. Specifically, an additional polarization-insensitive, OSS-constrained Grid 2 library subset was constructed by including both TE- and TM-polarized responses during the unit-cell optimization. Unlike the auxiliary single-zone design discussed above, this library subset was generated under the same angle-discretized and OSS-constrained framework used in the main text. This subset covers $\phi = 0°$ and $5°$ with $\theta = 2°, 4°, 6°, 8°$, and $10°$, including the four boundary-adjacent unit cells used for the representative Q-type stitched-supercell analysis. Therefore, this analysis tests whether TE/TM polarization robustness can be added to the library construction without sacrificing the OSS-enforced structural similarity between neighboring angular zones that is needed to suppress stitching errors.

Figure S5 summarizes the resulting polarization-insensitive, OSS-constrained Grid 2 library subset and its stitched-supercell response. Figure S5(a) shows representative unit cells from the newly constructed library subset, covering $\phi = 0°$ and $5°$ with $\theta = 2°, 4°, 6°, 8°$, and $10°$. This subset retains the same angle-discretized structure as the original OSS-constrained Grid 2 library, while the optimization objective explicitly includes both TE and TM illumination.

Figure S5(b) shows the isolated optical-efficiency spectra for four representative boundary-adjacent unit cells, $\mathrm{UC}_{0°,8°}$, $\mathrm{UC}_{0°,10°}$, $\mathrm{UC}_{5°,8°}$, and $\mathrm{UC}_{5°,10°}$, evaluated under separate TE- and

TM-polarized illumination. These four unit cells correspond to the angular zones forming the representative Q-type boundary used for the stitched-supercell analysis. For each unit cell, the spectra are shown at the center polar angle and at the two neighboring sampled angles, $\theta_0 - 1^\circ$, θ_0, and $\theta_0 + 1^\circ$, while keeping the corresponding azimuthal angle ϕ fixed.

To quantify the angular robustness of these isolated unit cells, we computed the target-band-averaged optical efficiencies over all incidence conditions plotted in Fig. S5(b). Across the four representative unit cells and the three sampled polar angles for each unit cell, the RGB target-band mean efficiencies are 77.9% for TE-polarized incidence and 82.7% for TM-polarized incidence, corresponding to a TE/TM-averaged value of 80.3%. The corresponding channel-resolved TE/TM-averaged efficiencies are 88.4%, 75.2%, and 77.2% for the red, green, and blue target bands, respectively. The RGB target-band mean efficiency ranges from 75.2% to 79.5% for TE incidence and from 80.9% to 84.0% for TM incidence over the plotted angle set. These results indicate that the isolated boundary-adjacent unit cells maintain wavelength-selective color-routing behavior over the local angular range shown in Fig. S5(b).

For quantitative comparison with the stitched-supercell calculation, the representative boundary cells were also evaluated at the common boundary incidence condition $(\phi, \theta) = (2.5^\circ, 9^\circ)$. At this incidence condition, the target-band-averaged optical efficiencies remain high for both polarization states. Averaged over the four representative unit cells, the RGB mean efficiencies are 77.6% for TE-polarized incidence and 82.1% for TM-polarized incidence, corresponding to a TE/TM-averaged value of 79.8%. The channel-resolved TE/TM-averaged efficiencies are 88.0%, 74.6%, and 77.0% for the red, green, and blue target bands, respectively. Thus, although a small TE–TM difference remains, both polarization states preserve high wavelength-selective routing performance in the representative boundary-adjacent cells.

We then evaluated whether the same polarization-insensitive library subset remains compatible after stitching neighboring angular zones. Figures S5(c) and S5(d) compare the isolated-unit-cell spectra with the corresponding spectra extracted from a stitched 6×6 Q-type supercell under TE- and TM-polarized illumination, respectively. The Q-type supercell consists of $\mathrm{UC}_{5^\circ,10^\circ}$, $\mathrm{UC}_{5^\circ,8^\circ}$, $\mathrm{UC}_{0^\circ,8^\circ}$, and $\mathrm{UC}_{0^\circ,10^\circ}$ assigned to the four quadrants Q1–Q4. The stitched-supercell spectra were evaluated at the boundary incidence condition $(\phi, \theta) = (2.5^\circ, 9^\circ)$ and compared against the corresponding isolated-unit-cell spectra under the same incidence condition.

The stitched-supercell analysis shows that the spectral response is largely preserved after stitching. Using the RGB-concatenated relative L_2 difference between the isolated and stitched spectra as the stitching-error metric, the average stitching errors are 6.15% for TE polarization and 5.59% for TM polarization, corresponding to a TE/TM-averaged stitching error of 5.87%. These results indicate that the polarization-insensitive unit cells can be stitched into the representative OSS-constrained Q-type supercell with only moderate spectral deviation from the isolated-unit-cell references.

These results extend the conclusions of the polarization study in two ways. First, they show that wavelength-selective color routing with high target-band efficiency can be maintained when both TE- and TM-polarized illumination are explicitly included in the optimization objective. Second, they demonstrate that the resulting polarization-insensitive unit cells can still be stitched into a representative OSS-constrained supercell with only moderate spectral deviation from the isolated-unit-cell references. Therefore, while the main text uses a 45° linearly polarized excitation for computational efficiency, the present analysis demonstrates, for a representative oblique-angle library subset and stitched Q-type boundary, that the proposed OSS-constrained color-router framework can be extended toward polarization-insensitive library construction by explicitly incorporating

both TE and TM responses into the optimization objective.

S2.5. Photodetector model study

Throughout this work, the photodetector array beneath the color-router design region is modeled using a constant dielectric permittivity corresponding to silicon nitride ($\varepsilon_r = 4.41$), rather than a dispersive and absorptive silicon medium. This approximation is primarily introduced to reduce computational cost. In particular, the high refractive index of silicon ($n \approx 3.8$–5.6 over the 400–700 nm wavelength range) requires a significantly finer spatial discretization in finite-difference simulations, thereby substantially increasing the computational burden.

To assess the impact of this approximation, we re-evaluated representative unit cells using a Lorentzian model for silicon that captures the dispersive refractive index and non-negligible extinction coefficient across the visible range, as shown in Figure S6(a). The silicon photodetector model includes a dispersive silicon photodetector array with deep-trench isolation (DTI) between neighboring photodetector regions. The optical-efficiency spectra obtained using the SiN-equivalent photodetector model and the silicon photodetector array are compared in Figure S6(b)–(d).

For the innermost unit cell UC_{0° under normal incidence, the spectral shapes of all three color channels are well preserved under the silicon photodetector model. The band-averaged optical efficiency decreases from 91.9% to 82.4%, corresponding to an absolute reduction of 9.5 percentage points. We also evaluate representative oblique-incidence cases. For $\mathrm{UC}_{0^\circ,20^\circ}$ under $(\phi, \theta) = (0^\circ, 20^\circ)$ incidence, the band-averaged optical efficiency decreases from 85.9% to 77.8%, corresponding to an absolute reduction of 8.1 percentage points. For $\mathrm{UC}_{45^\circ,20^\circ}$ under $(\phi, \theta) = (45^\circ, 20^\circ)$ incidence, the efficiency decreases from 84.6% to 76.2%, corresponding to an absolute reduction of 8.4 percentage points.

Although the silicon photodetector model leads to a moderate reduction in the absolute optical efficiency, the spectral selectivity and color-routing behavior of the red, green, and blue channels are retained for both normal and oblique incidence. The efficiency reduction is mainly attributed to the high refractive index and absorption of silicon in the visible range. In practical CIS stacks, however, the photodetector region is typically accompanied by additional optical layers such as anti-reflection coatings and passivation layers, which can reduce the reflection penalty associated with the high refractive index of silicon. Similar anti-reflection treatments are also generally known to be incorporated in commercial nanophotonic color-router or nanoprism-type image-sensor stacks. Therefore, the efficiency reduction observed here should be interpreted as a conservative estimate based on a simplified silicon photodetector model, rather than as a fundamental limitation of the proposed color-router design. These results confirm that the lower-permittivity approximation for the photodetector array preserves the essential color-routing functionality of the optimized designs.

Note S3. Two-dimensional angle-specific color routers

The 2D model is used as a numerically transparent proof-of-concept for isolating stitching-induced degradation before treating the full 3D problem. Each 2D unit cell consists of a $1.5\ \mu\mathrm{m} \times 2.0\ \mu\mathrm{m}$ design region above three laterally arranged photodetector regions. Independent optimizations were carried out for multiple target polar angles, and the resulting designs were subsequently stitched into long 2D supercells to separate genuine interface effects from finite-domain artifacts.

S3.1. Isolated unit-cell performance

Figures S7 and S8 show representative optimization histories and isolated optical responses for UC_{8° and UC_{12°, respectively. In both cases, the color-channel figures of merit rise rapidly during the early stage of the optimization, while the projection schedule steadily drives the design toward a binary silica/silicon-nitride distribution. The final spectra remain well separated over the sampled incident-angle range assigned to each unit cell, confirming that the isolated 2D designs achieve the intended angle-specific color-routing behavior before stitching.

S3.2. Long-supercell validation of stitching error

To verify that the spectral degradation observed after stitching originates from the structural discontinuity at the interface rather than from finite-supercell artifacts, we constructed elongated 40-cell supercells consisting of 20 copies of one unit cell and 20 copies of its neighboring unit cell. Cells far from the stitched interface remain surrounded by a nearly periodic local environment under the applied Bloch boundary conditions, so their responses closely reproduce the isolated references (e.g., L10 and R10 cells). By contrast, cells immediately adjacent to the interface experience a mismatched neighborhood and exhibit pronounced spectral distortion (e.g., L19, L20, R1, and R2

cells). The degradation is therefore localized to the stitched boundary itself and cannot be attributed to the finite supercell length.

Figure S9 shows the long-supercell analysis for the $\mathrm{UC}_{8^\circ}/\mathrm{UC}_{12^\circ}$ pair at the boundary angle $\theta = 10^\circ$. A similar interface-localized degradation trend is observed for the $\theta = 18^\circ$ case, as shown in Figure S10.

Note S4. Three-dimensional freeform angle-specific color routers

This note collects additional information for the 3D freeform libraries. Unless otherwise stated, dashed curves denote isolated unit-cell spectra under Bloch boundary conditions and solid curves denote spectra extracted from the corresponding boundary-adjacent cells in stitched supercells.

For convenience, we classify the two representative four-zone stitched-boundary layouts into *Q-type* and *H-type* configurations (Figure S11). The *Q-type* boundary denotes the quadrant-like arrangement in which the four angular regions meet in the usual four-quadrant form. The *H-type* boundary denotes a staggered, step-like four-zone junction, named as a shorthand description of its distinct local topology. These labels are introduced only as compact descriptors of the stitched-boundary geometries used in the supercell validation.

S4.1. Independently optimized unit cells (Grid 1)

We first consider a Grid 1 library built without OSS, with $(\Delta\phi, \Delta\theta) = (10^\circ, 4^\circ)$. The representative unit cells selected for stitched-supercell validation span both low- and high-azimuth branches so that Q-type and H-type four-zone junctions can be examined. As shown in Figure S12, the independently optimized geometries vary substantially from one angular zone to the next. The isolated optical performance of these cells is summarized in Figure S13. Although the isolated designs exhibit strong angle-specific color routing, Figure S14 shows that the stitched spectra near the junctions deviate markedly from the isolated references in all four tested boundary configurations. These results support the large stitching error discussed in the main text.

S4.2. Sequential construction and validation of OSS-constrained unit cells (Grid 1, OSS $\tau = 0.90$)

The construction of the angle-specific library follows a systematic sequential optimization procedure to ensure structural continuity across the entire angular space. The process starts with UC_{0°, which is optimized without an OSS constraint and serves as the reference for subsequent steps. From this initial cell, the first branch is generated along $\phi_0 = 0^\circ$, yielding $\mathrm{UC}_{0^\circ,4^\circ}$, $\mathrm{UC}_{0^\circ,8^\circ}$, $\mathrm{UC}_{0^\circ,12^\circ}$, $\mathrm{UC}_{0^\circ,16^\circ}$, and $\mathrm{UC}_{0^\circ,20^\circ}$. Each newly introduced unit cell is optimized under an OSS constraint with respect to the nearest previously optimized neighbor along θ, thereby enforcing gradual structural evolution along the branch.

Once this initial branch is established, additional branches are introduced at increasing ϕ_0. For example, along the $\phi_0 = 10^\circ$ branch shown in Figure S15(c), each new unit cell is optimized under OSS constraints with respect to the nearest previously optimized neighbors along both ϕ and θ. Repeating this outward-expansion rule progressively extends the library to larger azimuthal angles, as illustrated in Figure S15(d), up to $\phi_0 = 50^\circ$.

Following this procedure, we construct the complete 30-cell Grid 1 library. Figure S17 shows the resulting library (except UC_{0°), where unit cells evolve smoothly from one angular zone to the next. The optimization stability during this process is confirmed in Figure S16, which shows that the optical objective converges while strictly satisfying the imposed OSS constraints.

The isolated performance of these cells is evaluated in Figure S18, where representative spectra demonstrate that strong angle-specific color routing is maintained despite the structural constraints. Finally, Figure S19 provides the two additional Grid 1 stitched-boundary cases at $\theta = 18^\circ$; together with the $\theta = 10^\circ$ cases reported in Figure 5 of the main text, these results complete the four-

boundary Grid 1 validation. In all cases, the stitched spectra remain much closer to the isolated references than in the independently optimized library, consistent with the reduced stitching error reported in the main text.

S4.3. OSS-constrained unit cells (Grid 2, OSS $\tau = 0.95$)

Grid 2 uses finer angular step sizes, $(\Delta\phi, \Delta\theta) = (5^\circ, 2^\circ)$, together with a stricter OSS threshold of $\tau = 0.95$. The resulting 100-cell library (except UC_{0°) is shown in Figure S21. The optimization histories of representative unit cells are provided in Figure S20, confirming stable convergence under the stricter OSS requirement. Figure S22 confirms that representative unit cells maintain clear angle-specific color routing over their assigned angular zones. Figure S23 provides the two additional Grid 2 stitched-boundary cases at $\theta = 9^\circ$; together with the $\theta = 19^\circ$ cases reported in Figure 6 of the main text, these results complete the four-boundary Grid 2 validation. The close overlap between isolated and stitched spectra confirms that finer angular sampling and tighter OSS enforcement further suppress stitching-induced perturbations.

S4.4. Extension to Full 360° Azimuthal Coverage: Circular Branch and Wrap-around Closure

The freeform Grid 2 library reported in this work explicitly optimizes a 100-cell core sector spanning $\phi_0 = 0^\circ, 5^\circ, \ldots, 45^\circ$ and $\theta_0 = 2^\circ, 4^\circ, \ldots, 20^\circ$, together with the normal-incidence seed cell UC_{0°. To examine whether the same OSS-constrained construction can be continued over a full azimuthal circle, we additionally extend the library in ϕ_0 for the four smallest polar zones, $\theta_0 \in \{2^\circ, 4^\circ, 6^\circ, 8^\circ\}$. This choice targets the low-polar-angle region, where azimuthal variation is most frequent near the sensor center, while avoiding the substantially increased computational cost that would result from explicit full-360° optimization for every polar zone. The azimuthal

extension adds 248 optimized unit cells beyond the core sector, yielding 348 optimized unit cells in total, excluding the innermost cell.

Figure S25 summarizes this construction. Panel (a) shows the previously reported core sector. Panel (b) shows the circular branch obtained by continuing the sequential OSS-constrained optimization from $\mathrm{UC}_{45^\circ,\theta_0}$ through increasing ϕ_0 up to 350° for $\theta_0 \in \{2^\circ, 4^\circ, 6^\circ, 8^\circ\}$. Panel (c) highlights the final closure step at $\phi_0 = 355^\circ$. At this wrap-around seam, the terminal cell $\mathrm{UC}_{355^\circ,\theta_0}$ is simultaneously adjacent to two azimuthal neighbors, $\mathrm{UC}_{350^\circ,\theta_0}$ and $\mathrm{UC}_{0^\circ,\theta_0}$, as well as to the polar neighbor $\mathrm{UC}_{355^\circ,\theta_0-\Delta\theta}$. The closure optimization therefore requires simultaneous OSS matching to three reference cells, extending the single- and dual-reference construction used elsewhere in the library.

The resulting closure cells remain well behaved. For all four low-polar-angle branches, the optimized $\mathrm{UC}_{355^\circ,\theta_0}$ cells and their respective pairs satisfy OSS $\geq 95\%$, indicating that the three-reference closure converges without introducing an obvious structural discontinuity at the wrap-around seam. Their isolated optical responses are shown in Figure S26. The red, green, and blue target-channel spectra remain clearly separated over the assigned angular sets for $\mathrm{UC}_{355^\circ,2^\circ}$, $\mathrm{UC}_{355^\circ,4^\circ}$, $\mathrm{UC}_{355^\circ,6^\circ}$, and $\mathrm{UC}_{355^\circ,8^\circ}$. Averaged over the four closure cells, the mean optical efficiency is 84.5% and the mean optical crosstalk is 1.88%. These results confirm that the OSS framework can be extended to full azimuthal wrap-around while preserving strong angle-specific color routing in the closure branch.

Direct stitched-supercell validation of the wrap-around seam is provided in Figure S27. For all four polar zones $\theta_0 \in \{2^\circ, 4^\circ, 6^\circ, 8^\circ\}$, the optical-efficiency spectra of the boundary-adjacent cells (Q1–Q4) in the stitched supercells closely track their isolated references across the red, green, and blue channels, confirming that the OSS framework maintains stitching robustness at the azimuthal

wrap-around seam.

S4.5. Computational cost and scalability

To quantify the computational cost associated with the angle-specific library construction, we measured the wall-clock time required for unit-cell optimization in the present implementation. All unit-cell optimizations in this work were performed on a single workstation equipped with two AMD EPYC 9554 64-core processors, corresponding to 128 physical CPU cores and 256 hardware threads in total. One unit-cell optimization required approximately 3.5 h of wall-clock time when executed on the full CPU node. Based on the 128 physical CPU cores, this corresponds to approximately 448 core-hours per unit cell.

Using this estimate, the coarser Grid 1 sector library, which contains 30 optimized unit cells excluding the normal-incidence seed cell, corresponds to approximately 105 h of cumulative single-node wall-clock time, or 13,440 core-hours, under strictly sequential full-node execution. The finer Grid 2 sector library contains 100 optimized unit cells and therefore corresponds to approximately 350 h of cumulative single-node wall-clock time, or 44,800 core-hours. Thus, reducing the angular step sizes from $(\Delta\phi, \Delta\theta) = (10^\circ, 4^\circ)$ in Grid 1 to $(5^\circ, 2^\circ)$ in Grid 2 increases the number of optimized sector-library cells by a factor of approximately 3.3 in the demonstrated angular domain, with a proportional increase in cumulative optimization cost.

The same estimate also clarifies the cost of extending the angular library in azimuth. The explicit Grid 2 library including the low-polar-angle full-360° azimuthal extension contains 348 optimized unit cells, corresponding to approximately 1,218 h of cumulative single-node wall-clock time, or 155,904 core-hours. If the same Grid 2 angular discretization were explicitly extended over the full angular range $\phi_0 = 0^\circ, 5^\circ, \ldots, 355^\circ$ and $\theta_0 = 2^\circ, 4^\circ, \ldots, 20^\circ$, the resulting library

would contain 720 angle-specific unit cells, excluding the normal-incidence seed cell. This hypothetical complete Grid 2 angular library would require approximately 2,520 h, or about 105 days, of cumulative single-node wall-clock time under strictly sequential execution, corresponding to approximately 322,560 core-hours.

These estimates should be interpreted as cumulative CPU costs for the present CPU-only implementation, rather than as a fundamental scalability limit of the OSS framework. The OSS-constrained library construction contains local sequential dependencies because neighboring reference cells must be available before optimizing a new unit cell. However, once the required reference cells are specified, different angular branches or available optimization frontiers can be distributed across independent compute nodes. Therefore, the elapsed calendar time can be substantially reduced by parallel execution, even though the cumulative core-hour count remains a useful measure of total computational effort.

In addition, the computational cost of unit-cell optimization can be significantly reduced by using server-scale CPU resources or GPU-accelerated FDTD solvers. Recent GPU-based FDTD implementations have reported speedups of one to two orders of magnitude over conventional CPU-based FDTD simulations. Such acceleration is directly relevant to the present adjoint-optimization workflow because each iteration is dominated by repeated forward and adjoint time-domain simulations. Therefore, larger angular libraries can be constructed more efficiently by combining parallel library generation with accelerated electromagnetic solvers.

Finally, we emphasize that practical full-sensor deployment does not require a separate inverse design for every physical pixel. Instead, the optimized library is indexed by chief-ray-angle conditions and can be reused across sensor regions with similar angular incidence. The relevant computational task is therefore the construction of a finite angular library, rather than full-pixel-

scale inverse design over the entire sensor plane. The angular discretization should be selected according to the desired trade-off among optical efficiency, stitching-error suppression, angular coverage, and computational budget.

S4.6. Sensitivity to the OSS threshold τ

The OSS threshold τ defines the minimum structural similarity enforced between neighboring angular zones during the sequential construction of the unit-cell library. To examine how this parameter affects the optical response and stitching behavior, we performed a sensitivity study on a representative subset of the Grid 1 library. Specifically, we considered the four-zone Q-type junction at $(\phi, \theta) = (5^\circ, 10^\circ)$, corresponding to the boundary configuration shown in Figure 5 of the main text. The four boundary-adjacent unit cells, $\mathrm{UC}_{0^\circ,8^\circ}$, $\mathrm{UC}_{0^\circ,12^\circ}$, $\mathrm{UC}_{10^\circ,8^\circ}$, and $\mathrm{UC}_{10^\circ,12^\circ}$, labeled Q1–Q4, were optimized using $\tau = 0.85$, 0.90, and 0.95, while all other optimization parameters were kept fixed. For each threshold, we evaluated two averaged metrics: the average isolated optical efficiency of the four unit cells at their respective design angles and the average stitching error of the assembled 6×6 supercell, computed using the same spectrally normalized L_2 metric used throughout the main text. Figures S24(a) and (b) compare the isolated unit-cell spectra and stitched-supercell spectra for $\tau = 0.85$ and $\tau = 0.95$, respectively, while Figure S24(c) summarizes the two averaged metrics as functions of τ. The $\tau = 0.90$ case is the Grid 1 design used in the main-text analysis.

As τ increases from 0.85 to 0.90 and 0.95, the average isolated optical efficiency decreases from 92.96% to 91.10% and 88.31%, whereas the average stitching error decreases from 12.83% to 10.91% and 9.19%. For comparison, the same boundary assembled from independently optimized unit cells without the OSS constraint exhibits a substantially larger stitching error of 27.6%.

Thus, the tested thresholds exhibit a smooth and monotonic trade-off between isolated-cell performance and stitching-error suppression, without any abrupt transition. Over the full interval from $\tau = 0.85$ to $\tau = 0.95$, the stitching error decreases by 3.64 percentage points, while the isolated optical efficiency decreases by 4.65 percentage points. Importantly, all OSS-constrained cases reduce the stitching error to less than half of the unconstrained value, indicating that stitching-error suppression is a robust consequence of the OSS constraint rather than an effect that depends sensitively on a particular threshold value.

These results clarify the underlying design trade-off associated with the OSS threshold. A larger τ enforces stronger structural similarity between neighboring angular zones, thereby reducing the permittivity discontinuity at the stitching interface and suppressing the stitching error. At the same time, the stronger similarity constraint restricts the design space available to each unit cell and can therefore reduce the isolated optical efficiency. The appropriate threshold should thus be selected in conjunction with the angular grid resolution. In the coarser Grid 1 library, neighboring angular zones correspond to more widely separated incidence conditions, so an overly strict similarity requirement can unnecessarily penalize isolated-cell performance, as observed for the $\tau = 0.95$ case. The value $\tau = 0.90$ therefore provides a balanced choice for Grid 1, achieving substantial stitching-error suppression while preserving high isolated-cell efficiency. In the finer Grid 2 library, neighboring zones are intrinsically more similar in their target incidence conditions, allowing the stricter threshold $\tau = 0.95$ to be imposed with a smaller efficiency penalty while further suppressing stitching error. This trend is consistent with the improved stitching behavior of Grid 2 shown in the main text.

Note S5. Five-layer angle-specific color routers

This note provides additional results for the five-layer OSS-constrained design introduced in Section 3.3 of the main text, including representative optimization histories, the complete unit-cell library, isolated spectra, and complementary stitched-boundary validations. Compared with the freeform Grid 2 library, the five-layer designs exhibit somewhat lower isolated efficiencies, reflecting the reduced geometric degrees of freedom of the layered parameterization. Nevertheless, the stitched responses remain close to the isolated references, indicating that strong stitching robustness is preserved.

Representative optimization histories are shown in Figure S28, confirming that the optical objective converges stably while the structural-similarity constraint is maintained despite the reduced degrees of freedom. The complete 100-cell library is shown in Figure S29, where the design region retains the same overall footprint as the freeform case but is partitioned into five vertically stacked layers. The isolated optical-efficiency spectra of representative unit cells are provided in Figure S30; relative to the freeform Grid 2 cases, the five-layer spectra exhibit broader transitions and somewhat lower peak efficiencies, while still maintaining clear color-channel separation within the assigned angular ranges. Finally, Figure S31 provides the complementary stitched-boundary validations, where the stitched spectra remain close to the isolated references, confirming that the OSS framework preserves strong stitching robustness even in the reduced-complexity layered architecture.

Note S6. Supercell-size convergence

S6.1. Optical-efficiency spectra convergence

The stitched-supercell evaluations in this work are performed using a local 6×6 supercell, which provides a practical compromise between computational cost and sufficient spatial isolation of the stitched boundary under investigation. In a finite supercell calculation, the extracted response of a boundary-adjacent unit cell can in principle be affected not only by the local structural discontinuity at the intended stitching boundary, but also by the finite lateral extent of the computational domain and by interactions with periodically repeated copies of the supercell. Therefore, we examined whether the 6×6 domain used throughout this work is sufficiently large to provide a converged estimate of the local stitching response.

We compared optical-efficiency spectra obtained from otherwise identical 6×6 and 10×10 stitched-supercell configurations. The comparison was performed for representative boundary configurations covering the main cases considered in this work: a Freeform Grid 1 stitched boundary, a Freeform Grid 2 stitched boundary, and a 5-layer Grid 2 stitched boundary. For each representative case, the 6×6 and 10×10 simulations used the same local boundary geometry and the same incidence condition specific to that case. Specifically, the Freeform Grid 1 case was evaluated at $(\phi, \theta) = (5^\circ, 10^\circ)$, whereas the Freeform Grid 2 and 5-layer Grid 2 cases were evaluated at $(\phi, \theta) = (2.5^\circ, 9^\circ)$. The optical efficiencies were extracted from the four boundary-adjacent cells labeled Q1–Q4. The larger 10×10 supercell increases the separation between the analyzed boundary region and its periodically repeated replicas, and therefore serves as a stricter reference for assessing finite-domain effects.

For a quantitative comparison, we evaluated the normalized L_2 spectral difference between the

6×6 and 10×10 results for each boundary-adjacent cell and each color channel, expressed in percentage units:

$$\epsilon_{L_2}^{(q,c)} = 100 \left[\frac{1}{N_\lambda} \sum_{i=1}^{N_\lambda} \left(\mathrm{OE}_{6\times6}^{(q,c)}(\lambda_i) - \mathrm{OE}_{10\times10}^{(q,c)}(\lambda_i) \right)^2 \right]^{1/2}, \tag{S11}$$

where q denotes the boundary-adjacent cell index, c denotes the color channel, and N_λ is the number of sampled wavelengths. This metric is consistent with the L_2-type spectral error used in the main analysis, while being normalized by the number of wavelength samples to facilitate comparison between spectra. Since the optical efficiency is defined on a unitless 0–1 scale, the factor of 100 converts the resulting spectral difference into percentage units.

The normalized L_2 differences averaged over the four boundary-adjacent cells and the three color channels are 1.6%, 1.1%, and 0.8% for the Freeform Grid 1, Freeform Grid 2, and 5-layer Grid 2 cases, respectively. The corresponding maximum normalized L_2 differences among individual cell–channel spectra are 2.7%, 1.4%, and 1.0%, respectively. These values indicate that the spectral changes caused by increasing the supercell size from 6×6 to 10×10 are small for all tested boundary configurations.

The peak positions, passband shapes, and relative channel selectivities of the red, green, and blue optical-efficiency spectra are preserved when the supercell size is increased from 6×6 to 10×10. This trend is observed consistently for both Freeform designs and the 5-layer design.

S6.2. Field-profile convergence

The supercell-size dependence was also evaluated at the field-profile level using a normalized complex electric-field overlap. For each representative stitched boundary, the local field profile of

a boundary-adjacent cell embedded in the stitched supercell was compared with the corresponding isolated Bloch-periodic unit-cell field. The field overlap is defined as

$$M(q,\lambda) = \frac{|\langle \mathbf{E}_{\text{iso}}(q,\lambda), \mathbf{E}_{\text{sc}}(q,\lambda)\rangle|^2}{\langle \mathbf{E}_{\text{iso}}(q,\lambda), \mathbf{E}_{\text{iso}}(q,\lambda)\rangle \langle \mathbf{E}_{\text{sc}}(q,\lambda), \mathbf{E}_{\text{sc}}(q,\lambda)\rangle}, \tag{S12}$$

where $\mathbf{E}_{\text{iso}}$ and $\mathbf{E}_{\text{sc}}$ denote the complex electric fields obtained from the isolated unit-cell and stitched-supercell simulations, respectively. The inner products were evaluated over the same unit-cell design volume. The corresponding field-overlap error is defined as

$$e_{\text{field}}(q,\lambda) = 1 - M(q,\lambda). \tag{S13}$$

For each stitched-supercell configuration, e_{field} was evaluated for the four boundary-adjacent cells Q1–Q4 at the three RGB center wavelengths, resulting in 12 field-overlap error values. These values were summarized by their root-mean-square value, consistent with the L_2-type optical-efficiency stitching-error metric used in the main analysis.

For the Freeform Grid 2 Q-type junction at $(\phi,\theta) = (2.5°, 9°)$, the RMS field-overlap errors obtained from the 6×6 and 10×10 supercells were both 5.83%, differing by only 0.005 percentage points. The corresponding 6×6/10×10 differences were 0.86 percentage points for the Freeform Grid 1 case and 0.49 percentage points for the 5-layer Grid 2 case. The weak dependence of e_{field} on the supercell size indicates that the local field-profile perturbation is not dominated by the outer Bloch-periodic boundary or by the finite lateral extent of the computational domain.

Representative XZ cross-sections of $\text{Re}(\mathbf{E})$ for the Q1 boundary-adjacent cell are shown in Figure S33. For each case and wavelength, the isolated Bloch-periodic field is compared with the

corresponding local fields extracted from the 6×6 and 10×10 stitched supercells. The field maps are normalized by the maximum absolute $\mathrm{Re}(\mathbf{E})$ value among the isolated, 6×6, and 10×10 fields for each case and wavelength. The field profiles obtained from the 6×6 and 10×10 supercells exhibit close agreement, consistent with the small field-overlap-error differences quantified above.

Taken together, the optical-efficiency and field-profile convergence results confirm that the 6×6 supercell is sufficiently large to capture the local optical behavior of the stitched boundaries considered in this study. We therefore use the 6×6 stitched-supercell configuration for the systematic boundary evaluations reported in this work.

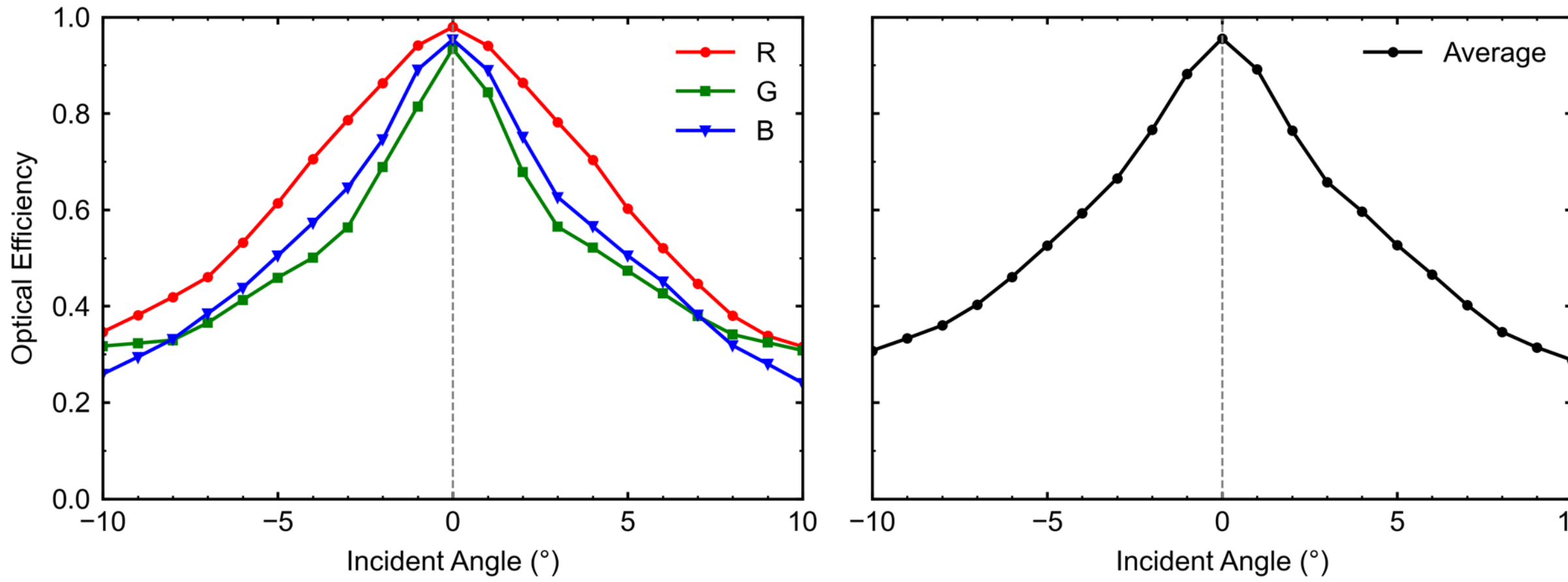

Fig S1: Angular response of a color router optimized exclusively for normal incidence. Left: red-, green-, and blue-channel optical efficiencies as functions of incident angle. Right: optical efficiency averaged over the three color channels. All channels exhibit a pronounced on-axis maximum, and the average efficiency decreases to approximately one-half of its peak value by $|\theta| \approx 6^{\circ}$.

Table S1: Polar-angle deviation $\Delta\theta = \theta_{\text{eff}} - \theta_0$ for a single broadband source centered at 550 nm, evaluated at representative wavelengths across the visible range. The deviation increases markedly toward the spectral edges, showing that a single fixed Bloch wavevector is inadequate for broadband oblique-incidence optimization.

θ_0 (deg)	420 nm	480 nm	520 nm	550 nm	580 nm	620 nm	680 nm
0	$+0.00$	$+0.00$	$+0.00$	$+0.00$	$+0.00$	$+0.00$	$+0.00$
4	-0.95	-0.51	-0.22	$+0.00$	$+0.22$	$+0.51$	$+0.95$
8	-1.90	-1.02	-0.44	$+0.00$	$+0.44$	$+1.03$	$+1.91$
12	-2.86	-1.55	-0.66	$+0.00$	$+0.67$	$+1.55$	$+2.90$
16	-3.85	-2.08	-0.89	$+0.00$	$+0.90$	$+2.10$	$+3.92$
20	-4.86	-2.63	-1.13	$+0.00$	$+1.14$	$+2.68$	$+5.02$
**22**	$\mathbf{-5.38}$	$\mathbf{-2.92}$	$\mathbf{-1.26}$	$\mathbf{+0.00}$	$\mathbf{+1.27}$	$\mathbf{+2.98}$	$\mathbf{+5.59}$

Table S2: Polar-angle deviation $\Delta\theta = \theta_{\text{eff}} - \theta_0$ for the three band-centered sources used in the optimization loop. For each source, the Bloch wavevector is fixed at the corresponding band-center wavelength and the deviation is evaluated at wavelengths ± 30 nm from the center. Since the direction of $\mathbf{k}_{\parallel}$ is unchanged, the azimuthal angle is unaffected and the mismatch is fully described by $\Delta\theta$.

θ_0 (deg)	R source (650 nm)		G source (550 nm)		B source (450 nm)	
	620 nm	680 nm	520 nm	580 nm	420 nm	480 nm
0	0	0	0	0	0	0
4	-0.18	$+0.18$	-0.22	$+0.22$	-0.27	$+0.27$
8	-0.37	$+0.37$	-0.44	$+0.44$	-0.54	$+0.54$
12	-0.56	$+0.56$	-0.66	$+0.67$	-0.81	$+0.81$
16	-0.76	$+0.76$	-0.89	$+0.90$	-1.09	$+1.10$
20	-0.96	$+0.97$	-1.13	$+1.14$	-1.38	$+1.40$
**22**	$\mathbf{-1.06}$	$\mathbf{+1.07}$	$\mathbf{-1.26}$	$\mathbf{+1.27}$	$\mathbf{-1.54}$	$\mathbf{+1.55}$

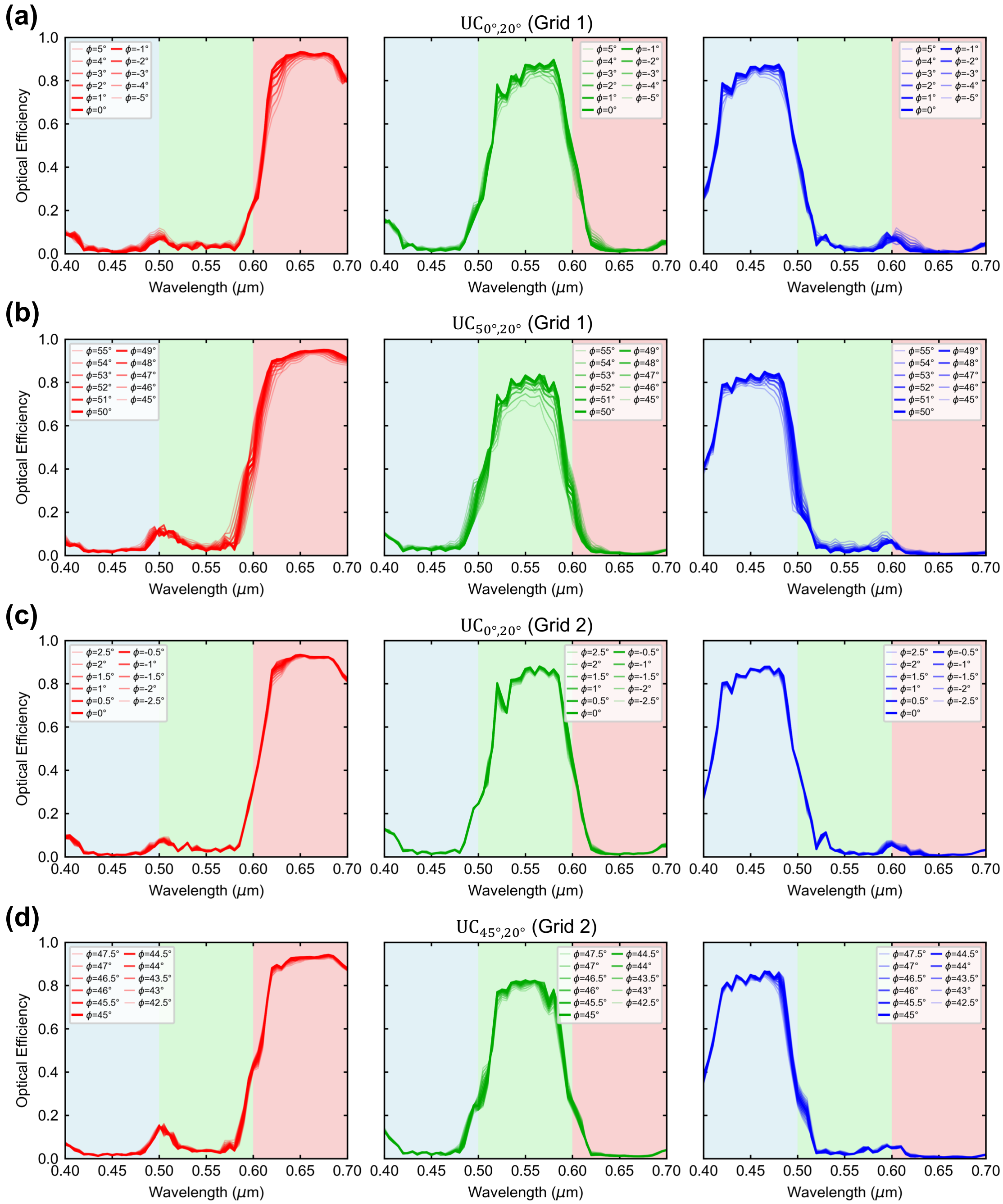

Fig S2: Azimuthal-angle dependence of representative OSS-constrained 3D freeform unit cells at the zone-center polar angle $\theta = \theta_0 = 20°$. Rows (a) and (b) correspond to $\mathrm{UC}_{0°,20°}$ and $\mathrm{UC}_{50°,20°}$ from Grid 1, and rows (c) and (d) correspond to $\mathrm{UC}_{0°,20°}$ and $\mathrm{UC}_{45°,20°}$ from Grid 2. In each row, the left, middle, and right panels show the optical efficiencies delivered to the red, green, and blue pixels, respectively, as the azimuth is varied around ϕ_0. Across all four cases, the target spectral responses remain nearly unchanged over the tested azimuthal offsets, indicating weak ϕ-dependence within the angular span of a zone.

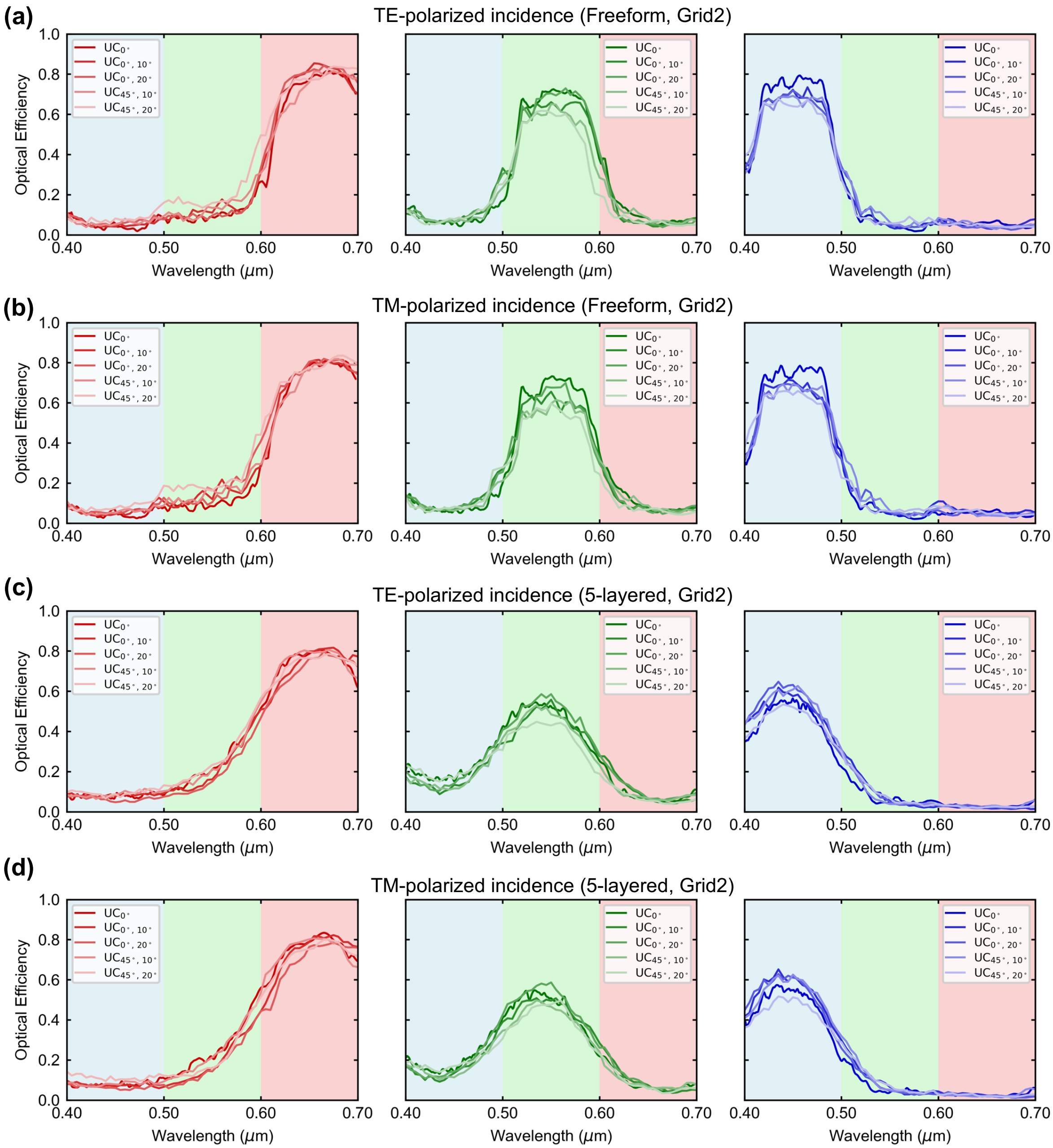

Fig S3: Polarization-resolved optical-efficiency spectra of representative unit cells from the main-text libraries. (a) TE-polarized response of five representative unit cells from the freeform Grid 2 library. (b) TM-polarized response of the same freeform Grid 2 unit cells. (c) TE-polarized response of five representative unit cells from the five-layered Grid 2 library. (d) TM-polarized response of the same five-layered Grid 2 unit cells. The shaded regions indicate the blue, green, and red target spectral bands. The representative unit cells preserve their wavelength-selective routing behavior under both TE- and TM-polarized illumination, although the absolute optical efficiency decreases compared with the 45° linearly polarized excitation used in the main text.

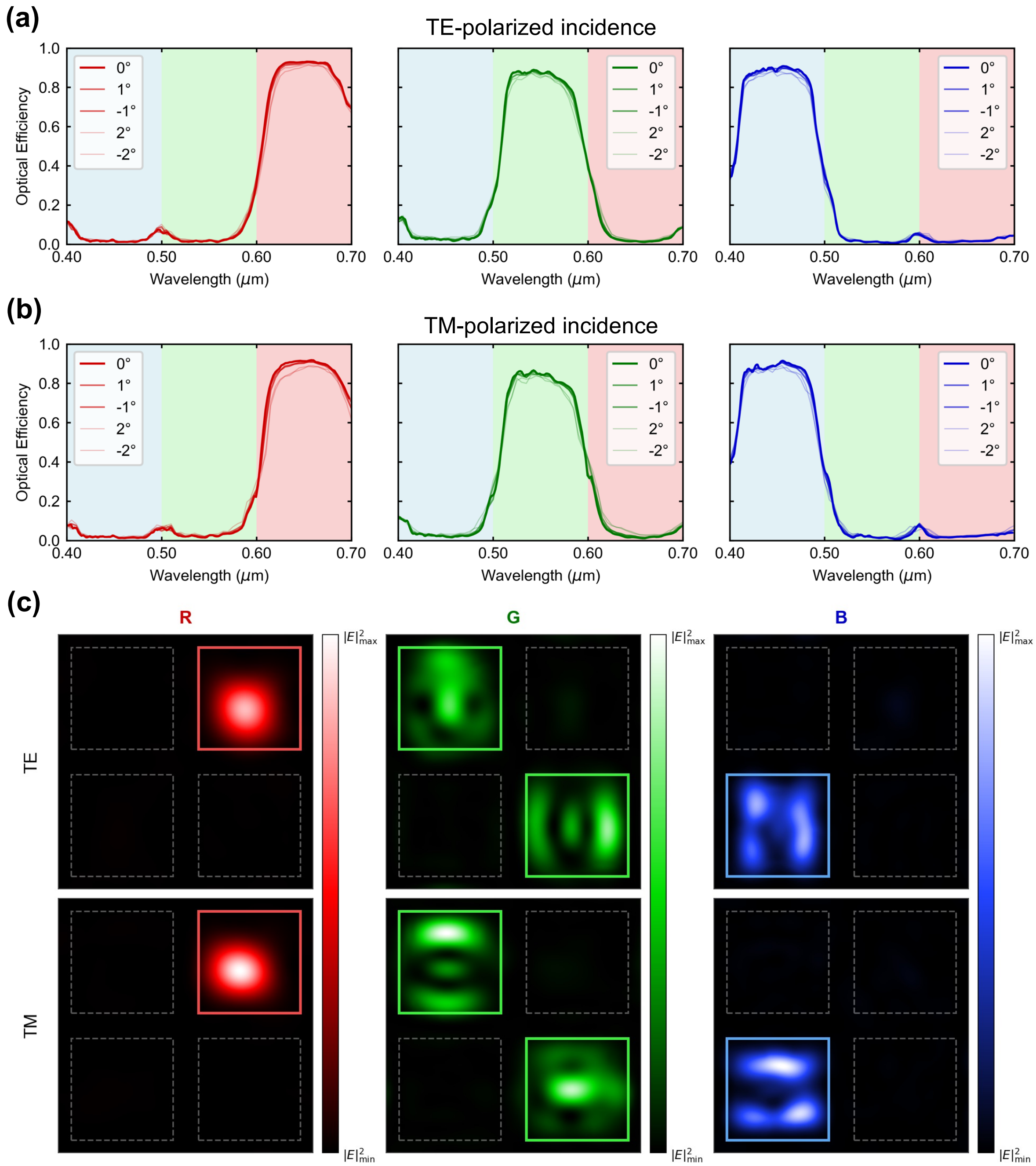

Fig S4: Polarization-resolved performance of the separately optimized auxiliary UC_{0° design. (a) Optical-efficiency spectra under TE-polarized illumination, evaluated at $\theta = -2^\circ, -1^\circ, 0^\circ, 1^\circ$, and 2°. (b) Corresponding optical-efficiency spectra under TM-polarized illumination. In both cases, the red, green, and blue target-channel responses remain clearly separated and maintain high efficiency over the full angular range assigned to the innermost zone. (c) Field-intensity distributions at the photodetector-array plane under normal incidence for TE- and TM-polarized illumination. In both polarization states, the optical intensity is routed predominantly to the intended photodetector region(s), confirming polarization-robust angle-specific color routing.

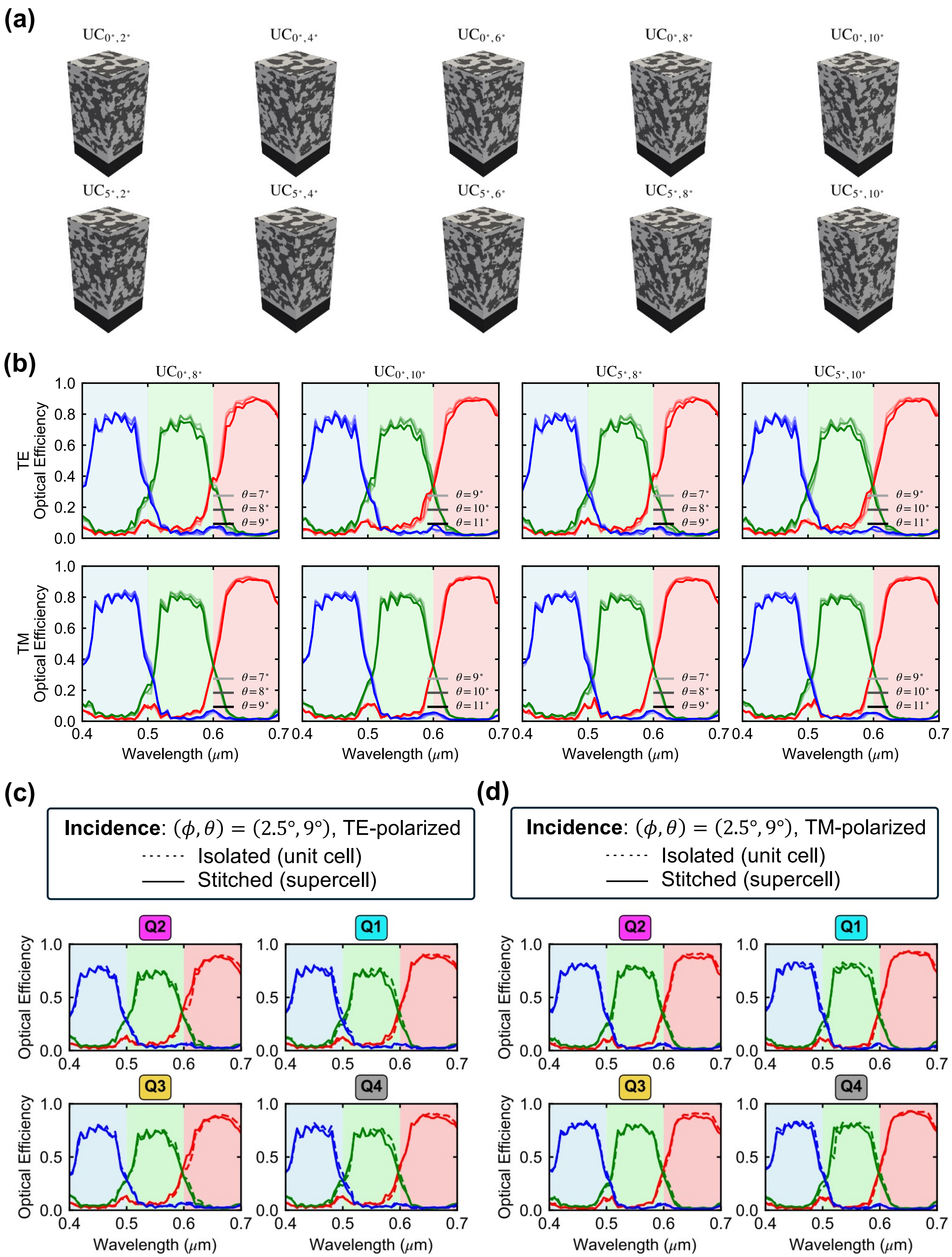

Fig S5: Polarization-insensitive, OSS-constrained Grid 2 library and stitched-supercell analysis. (a) Representative unit cells from the newly optimized library. (b) Isolated-unit-cell optical-efficiency spectra under TE- and TM-polarized illumination. (c,d) Comparison between isolated-unit-cell and stitched-supercell spectra under (c) TE- and (d) TM-polarized illumination.

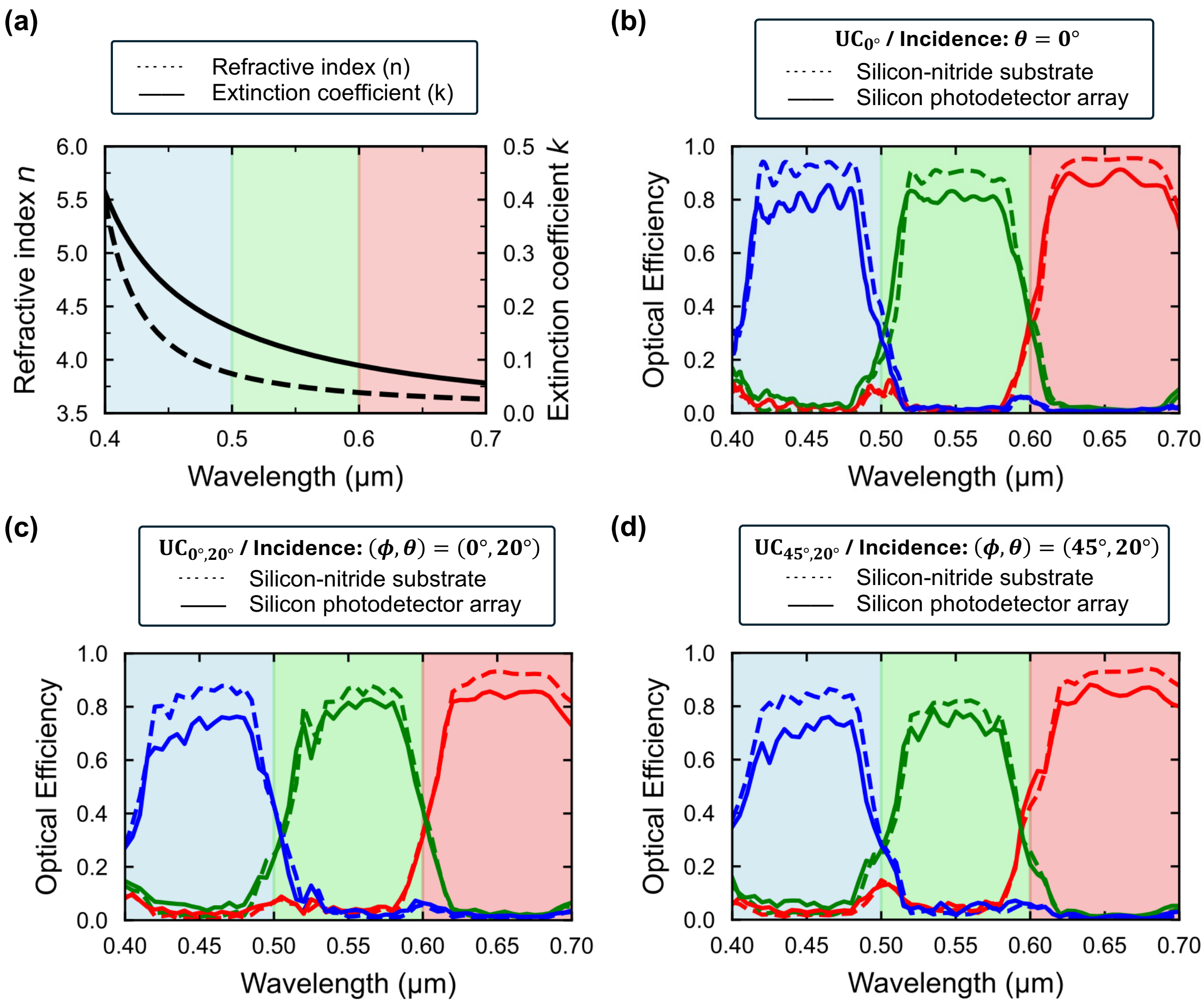

Fig S6: Validation of the lower-permittivity approximation for the photodetector array. (a) Optical constants of the silicon photodetector model across the visible wavelength range, obtained from a Lorentzian model. The refractive index n and extinction coefficient k are shown by the dashed and solid curves, respectively. (b) Optical-efficiency spectra for UC_{0° under normal incidence, comparing the SiN-equivalent photodetector model used during optimization with the dispersive silicon photodetector array including DTI. (c) Optical-efficiency spectra for $\mathrm{UC}_{0^\circ,20^\circ}$ under oblique incidence with $(\phi, \theta) = (0^\circ, 20^\circ)$. (d) Optical-efficiency spectra for $\mathrm{UC}_{45^\circ,20^\circ}$ under oblique incidence with $(\phi, \theta) = (45^\circ, 20^\circ)$. Red, green, and blue curves correspond to the respective color channels. The spectral shapes and color selectivity are preserved under the silicon photodetector model for both normal and oblique incidence, although the absolute efficiencies are moderately reduced.

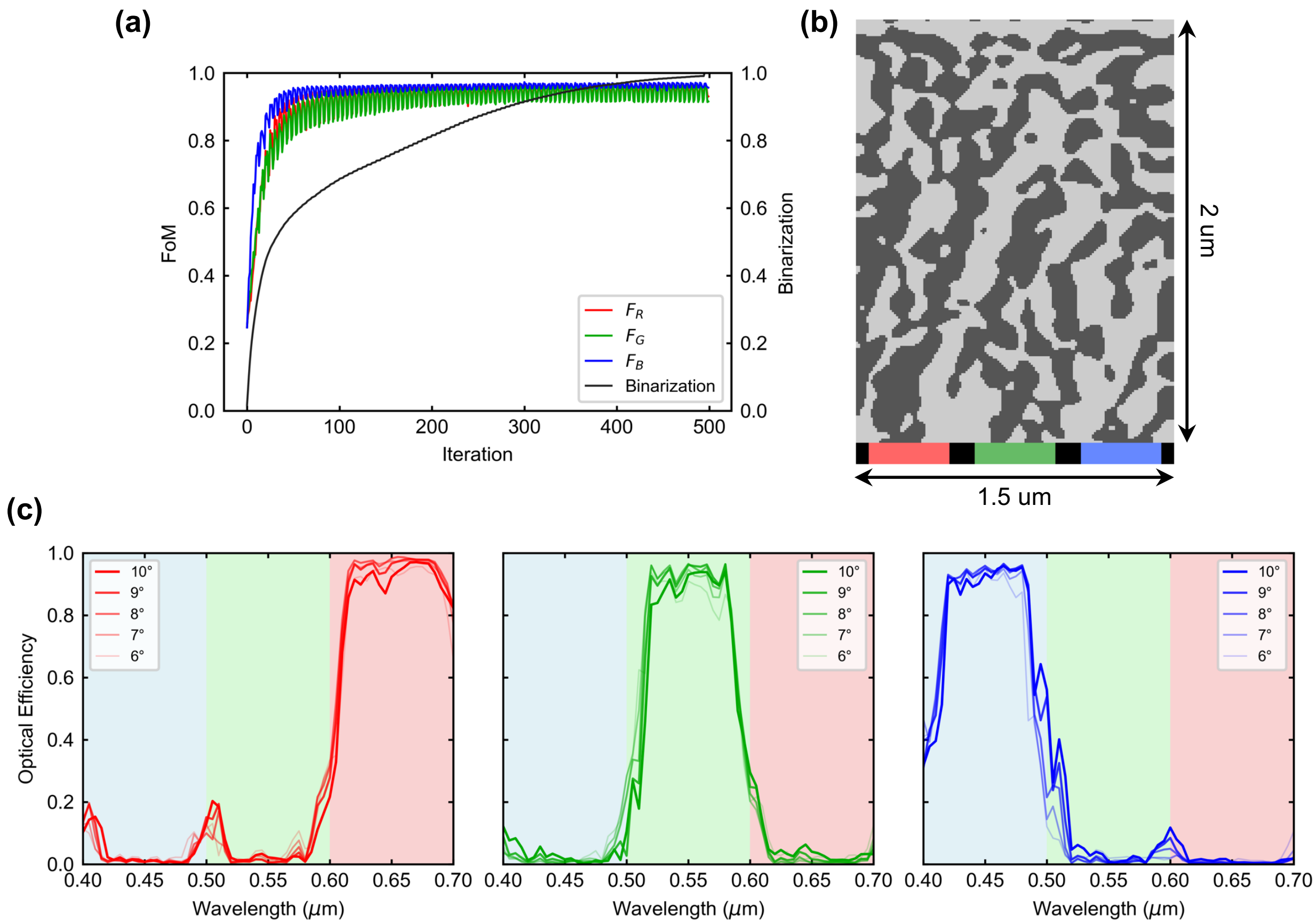

Fig S7: Optimization of the independently designed 2D unit cell UC_{8° for the angular zone centered at $\theta_0 = 8^\circ$ with $\Delta\theta = 4^\circ$. (a) Iteration-wise evolution of the red-, green-, and blue-channel figures of merit together with the binarization metric. (b) Final binarized structure in the $1.5\ \mu\mathrm{m} \times 2.0\ \mu\mathrm{m}$ design region. (c) Optical-efficiency spectra for the sampled incident angles within the assigned zone ($\theta = 6^\circ$–10°). Blue, green, and red shaded regions indicate the target spectral bands.

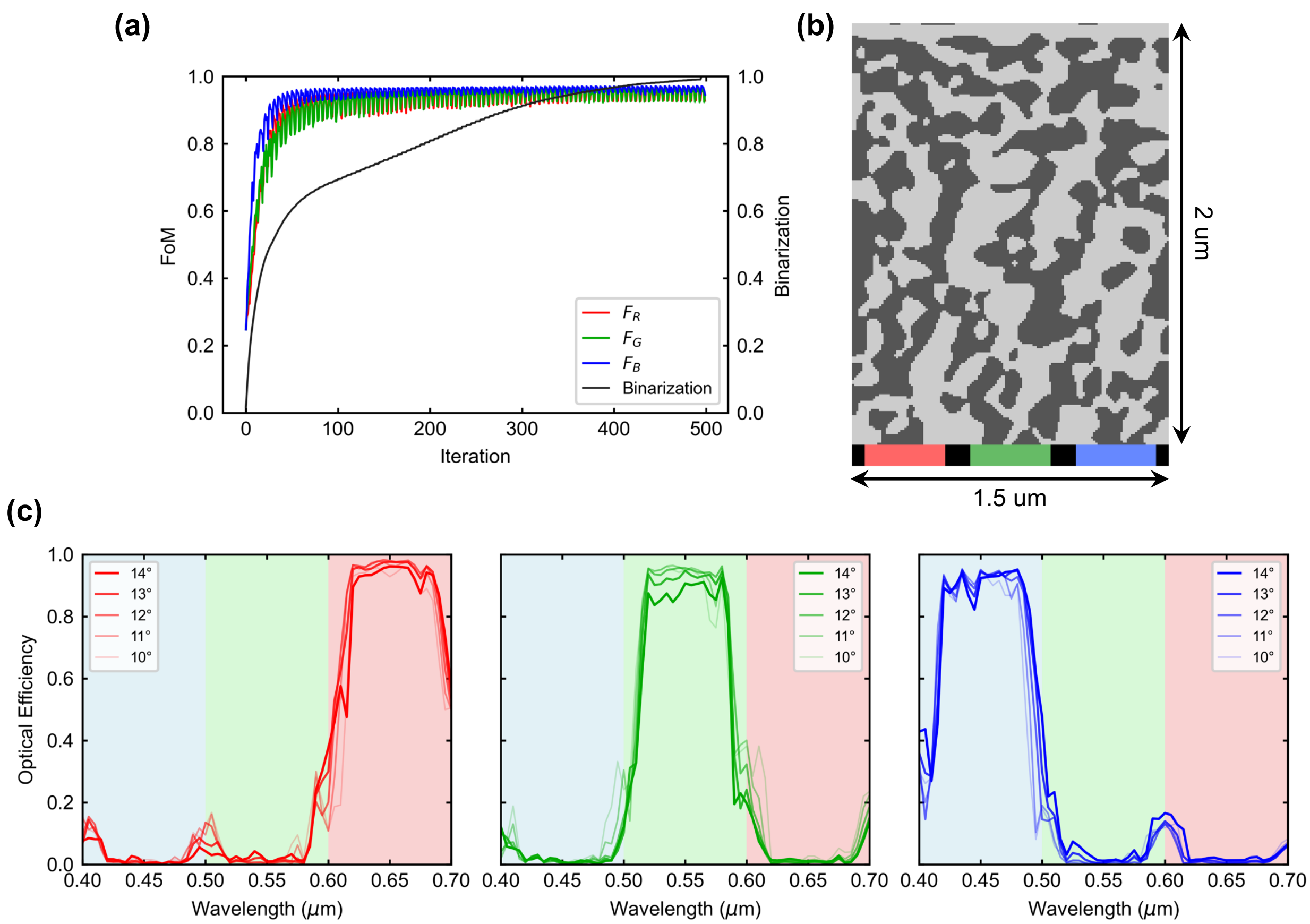

Fig S8: Optimization of the independently designed 2D unit cell UC_{12° for the angular zone centered at $\theta_0 = 12^\circ$ with $\Delta\theta = 4^\circ$. (a) Iteration-wise evolution of the channel-wise figures of merit together with the binarization metric. (b) Final binarized structure. (c) Optical-efficiency spectra for the sampled incident angles within the assigned zone ($\theta = 10^\circ$–14°). The isolated spectra remain clearly separated across the red, green, and blue target bands.

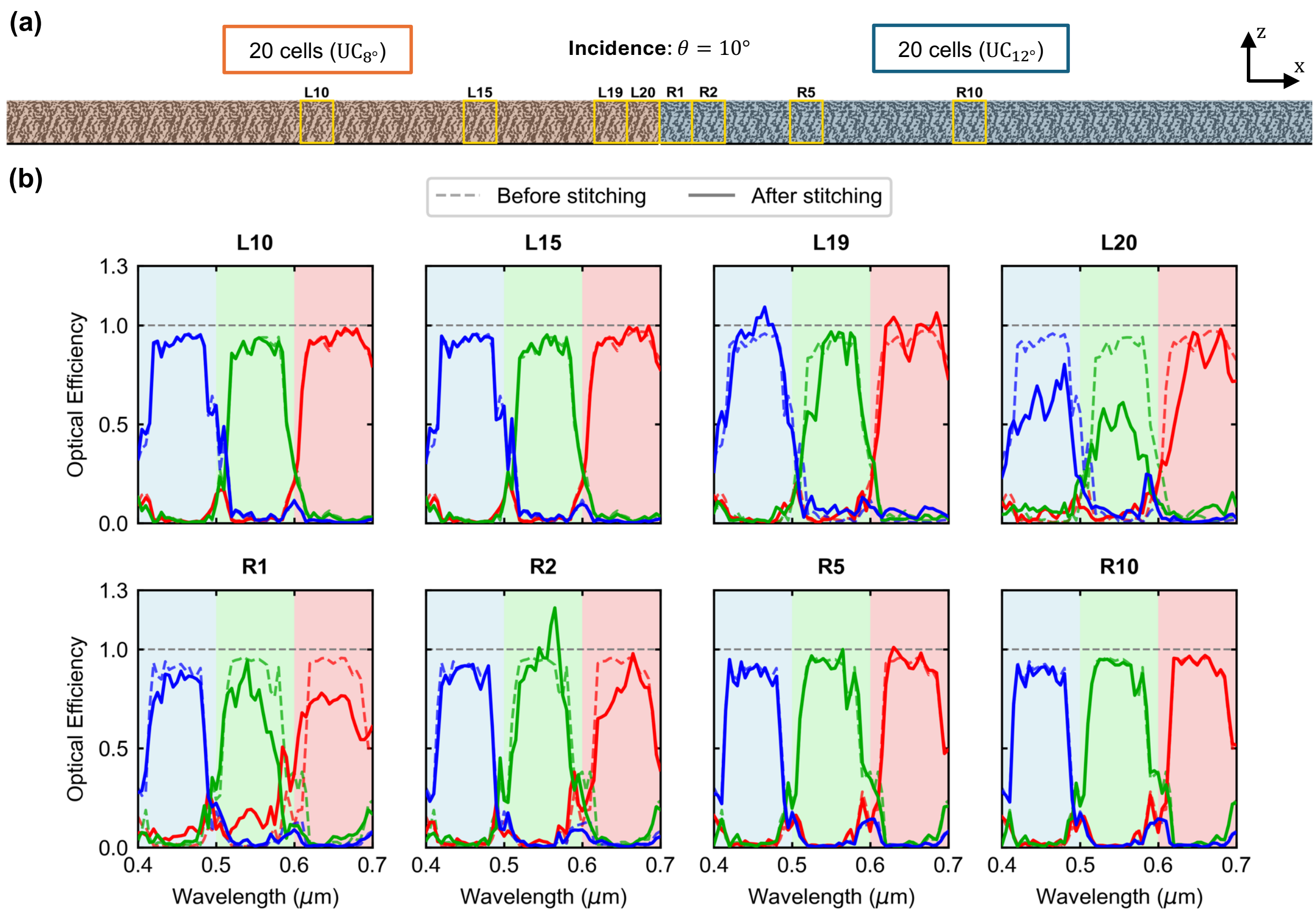

Fig S9: Long-supercell stitching analysis for the independently optimized 2D pair UC_{8° and UC_{12° at the boundary angle $\theta = 10^\circ$. (a) Forty-cell supercell composed of 20 copies of UC_{8° (left) and 20 copies of UC_{12° (right), with the marked positions L10, L15, L19, L20, R1, R2, R5, and R10 indicating the cells from which spectra are extracted. (b) Comparison of isolated responses (dashed, before stitching) and stitched-supercell responses (solid, after stitching) at the marked positions. Cells far from the interface, such as L10 and R10, closely reproduce the isolated Bloch-periodic responses, whereas cells immediately adjacent to the interface, such as L20, R1, and R2, exhibit pronounced spectral distortion across all three color channels.

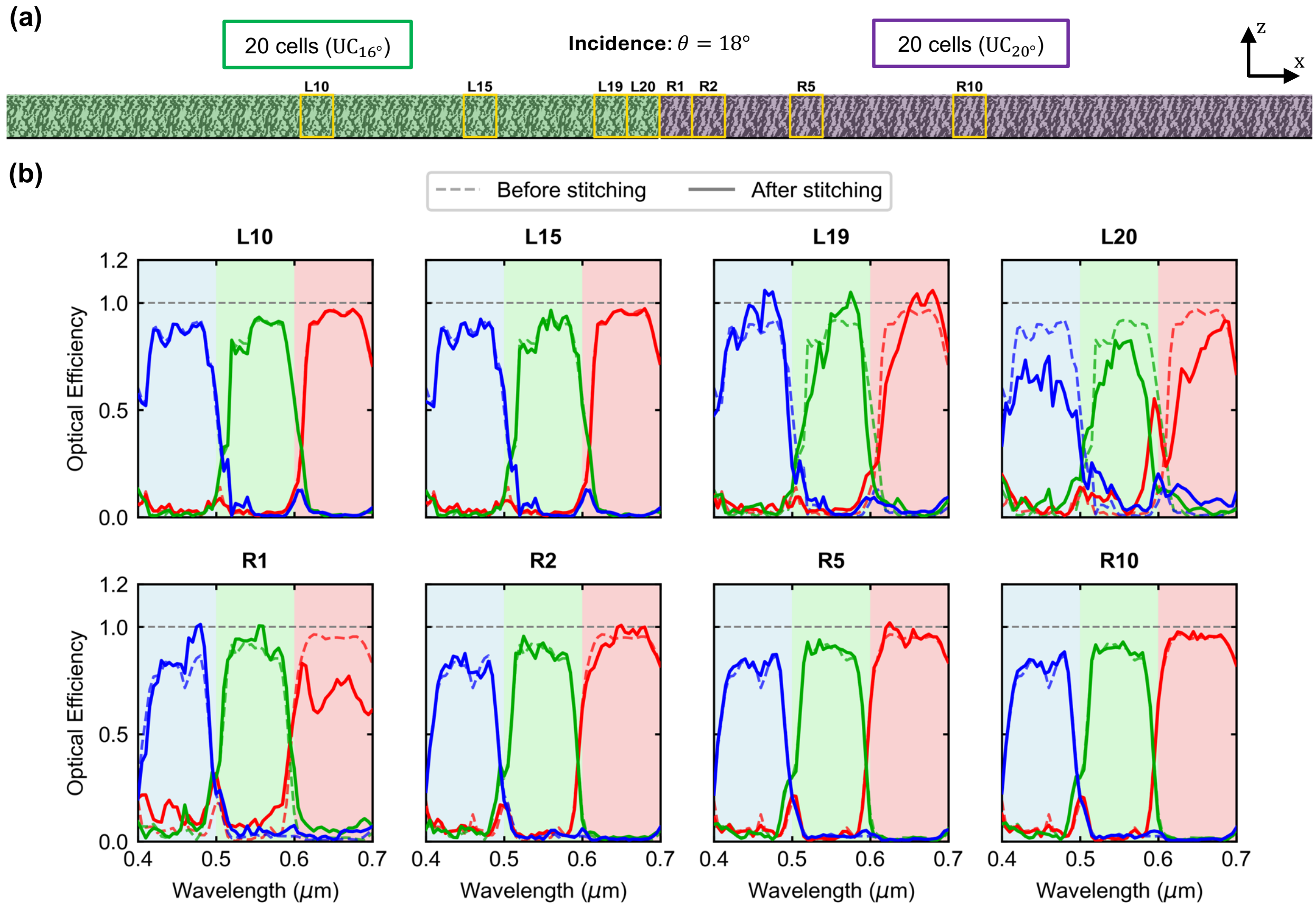

Fig S10: Long-supercell stitching analysis for the independently optimized 2D pair UC_{16° and UC_{20° at the boundary angle $\theta = 18^\circ$. The supercell is composed of 20 copies of UC_{16° and 20 copies of UC_{20°, with spectra extracted from the same set of marked positions as in Figure S9. The same spatial trend is observed: cells far from the interface remain close to their isolated references, while the boundary-adjacent cells show severe spectral degradation.

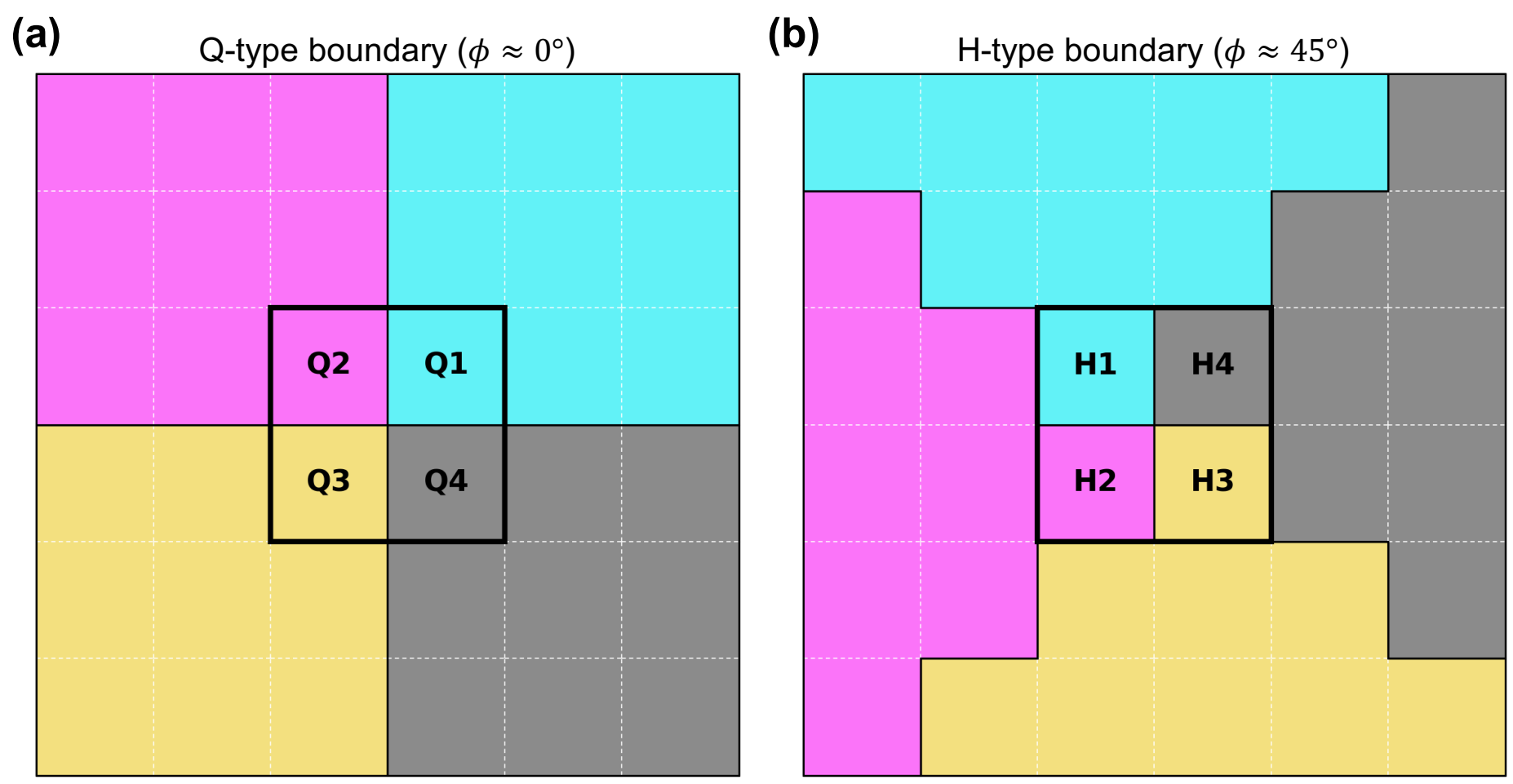

Fig S11: Schematic classification of the representative four-zone stitched-boundary configurations used in the stitched-supercell validation. (a) *Q-type* boundary, corresponding to a quadrant-like four-region junction near $\phi \approx 0°$. (b) *H-type* boundary, corresponding to a staggered, step-like four-zone junction near $\phi \approx 45°$. In each case, the boxed 2×2 region denotes the boundary-adjacent cells extracted for spectral comparison in the stitched-supercell analysis.

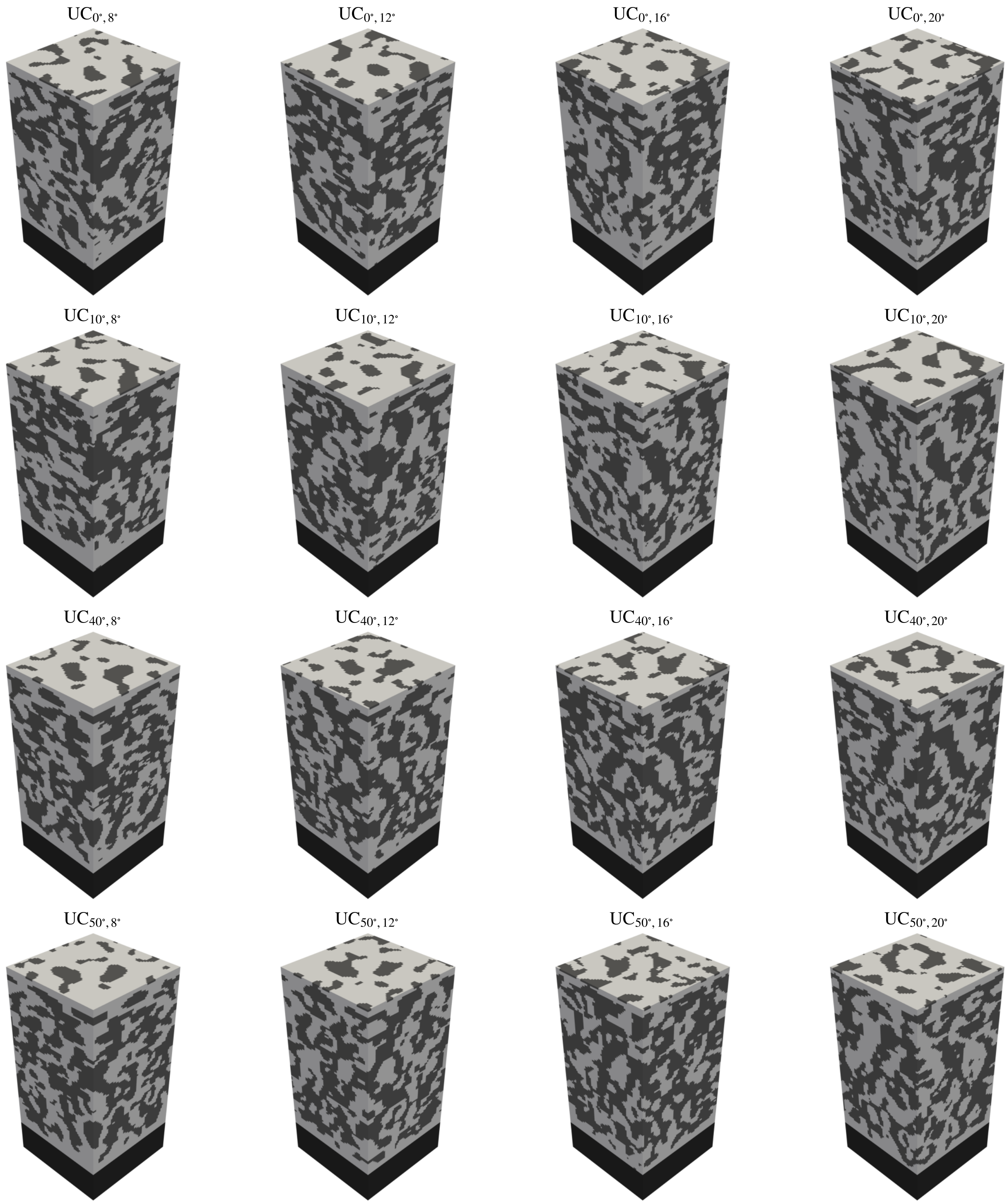

Fig S12: Representative independently optimized 3D freeform unit cells used in the stitched-supercell validation of Figure S14. The selected set spans low- and high-azimuth branches ($\phi_0 = 0°$, $10°$, $40°$, and $50°$) and four representative polar angles ($\theta_0 = 8°$, $12°$, $16°$, and $20°$). These 16 unit cells form the two Q-type boundaries ($\phi_0 = 0°/10°$) and two H-type boundaries ($\phi_0 = 40°/50°$) examined in Figure S14.

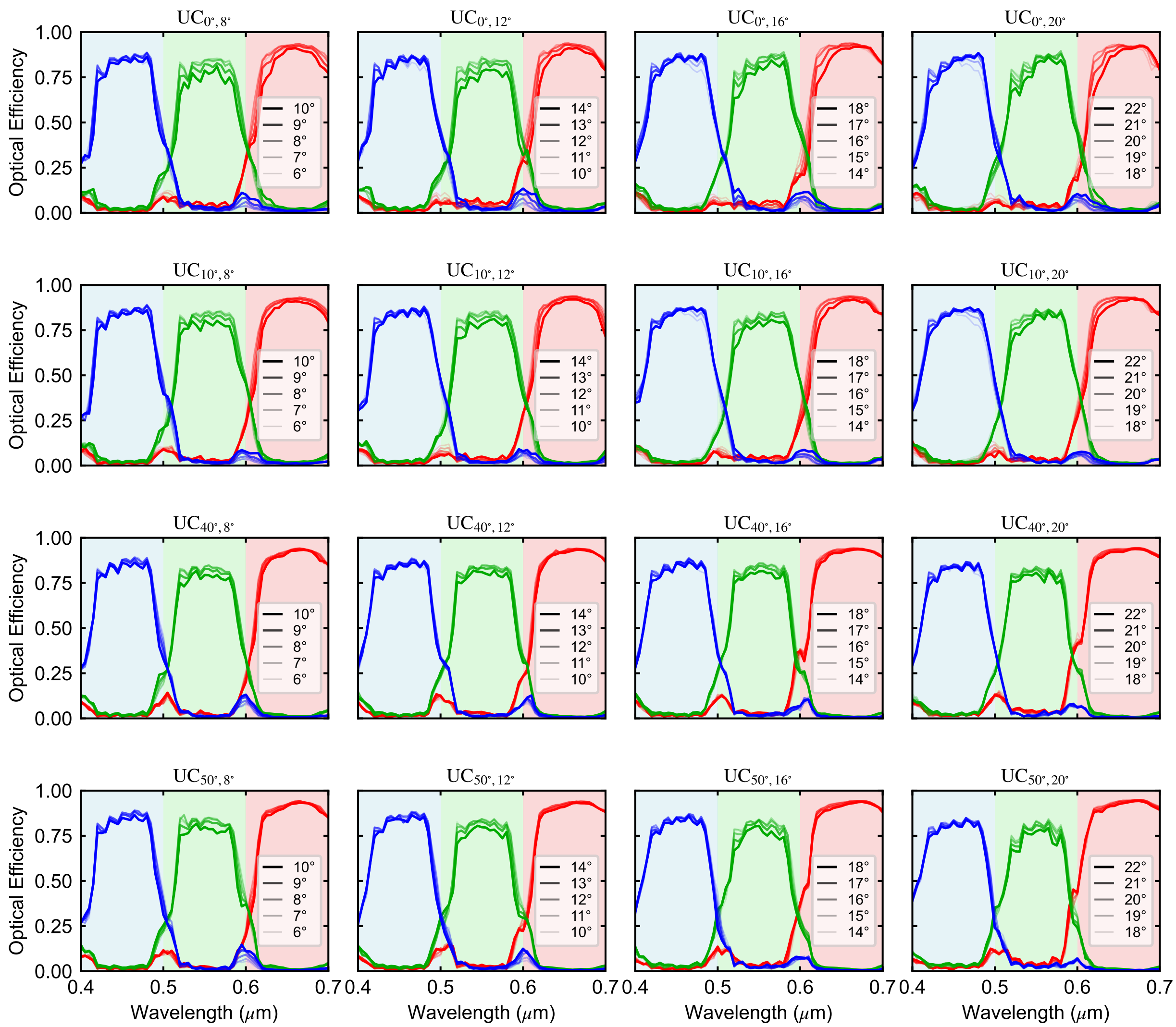

Fig S13: Isolated optical-efficiency spectra of representative independently optimized unit cells on Grid 1. The selected cells are $\mathrm{UC}_{\phi_0,\theta_0}$ for $\phi_0 \in \{0°, 10°, 40°, 50°\}$ and $\theta_0 \in \{8°, 12°, 16°, 20°\}$, corresponding to the same 16 cells used in Figure S12. Each panel reports the spectra over the sampled polar-angle set assigned to that unit cell, demonstrating strong optical performance.

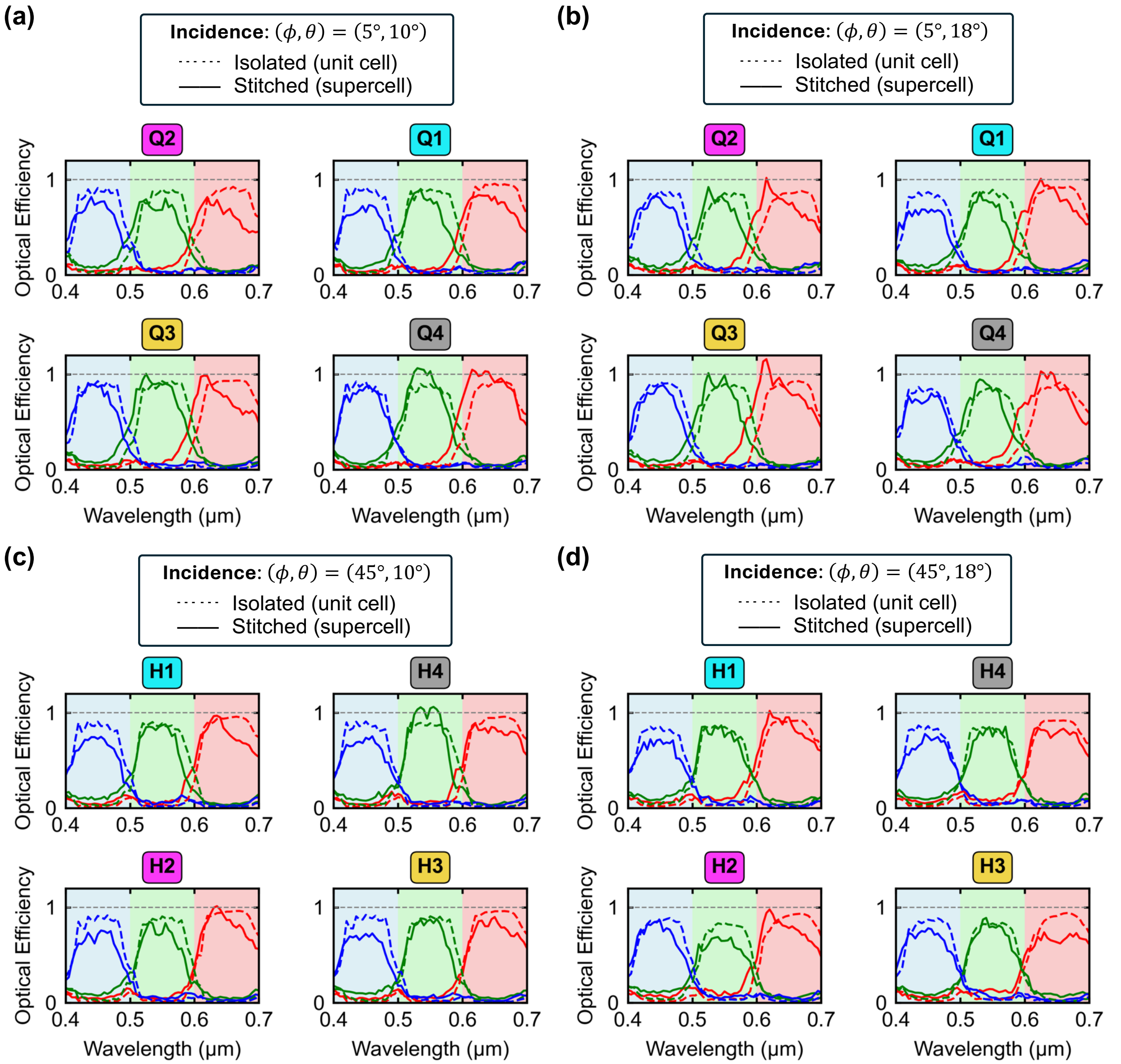

Fig S14: Complete stitched-supercell validation of the independently optimized 3D freeform library on Grid 1 $[(\Delta\phi, \Delta\theta) = (10^\circ, 4^\circ)]$ for four representative boundary configurations: (a) Q-type at $(\phi, \theta) = (5^\circ, 10^\circ)$, (b) Q-type at $(\phi, \theta) = (5^\circ, 18^\circ)$, (c) H-type at $(\phi, \theta) = (45^\circ, 10^\circ)$, and (d) H-type at $(\phi, \theta) = (45^\circ, 18^\circ)$. In all four cases, the stitched spectra near the four-zone junctions deviate substantially from the isolated references, confirming the strong stitching-induced degradation of independently optimized neighboring unit cells.

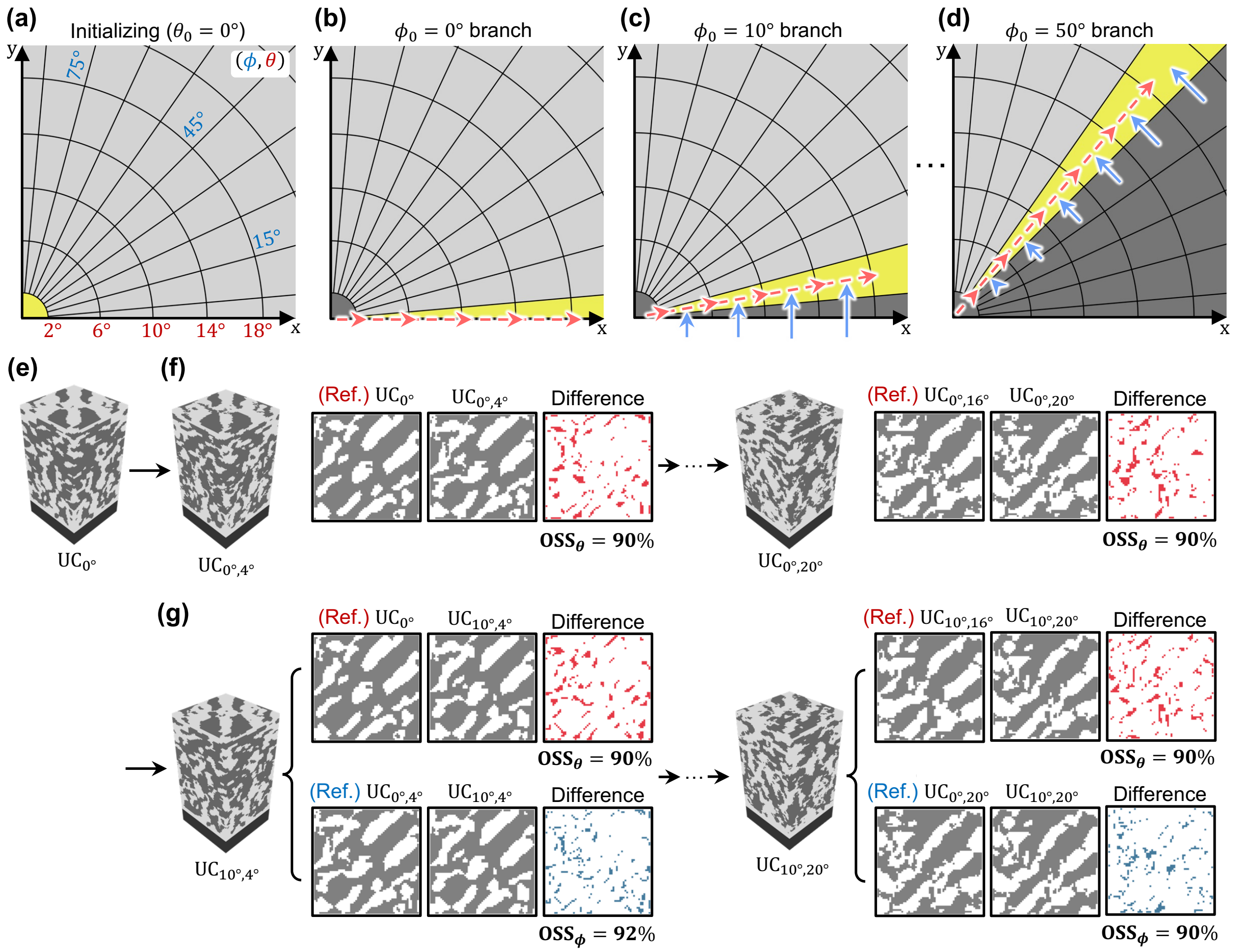

Fig S15: Sequential OSS-constrained construction and structural evolution of the 3D angle-specific unit-cell library on Grid 1 with $(\Delta\phi, \Delta\theta) = (10°, 4°)$ and $\tau = 0.90$. (a–d) Angular-space expansion from the seed cell $UC_{0°}$: initialization, growth of the $\phi_0 = 0°$ branch under nearest-neighbor OSS in θ, initiation of the $\phi_0 = 10°$ branch under OSS in both ϕ and θ, and continued outward expansion to $\phi_0 = 50°$. (e) A 3D layout of $UC_{0°}$. (f,g) Mid-height xy slices, reference slices, and difference maps for representative neighbors on the $\phi_0 = 0°$ and $\phi_0 = 10°$ branches, showing smooth structural adaptation under single- and dual-neighbor OSS constraints.

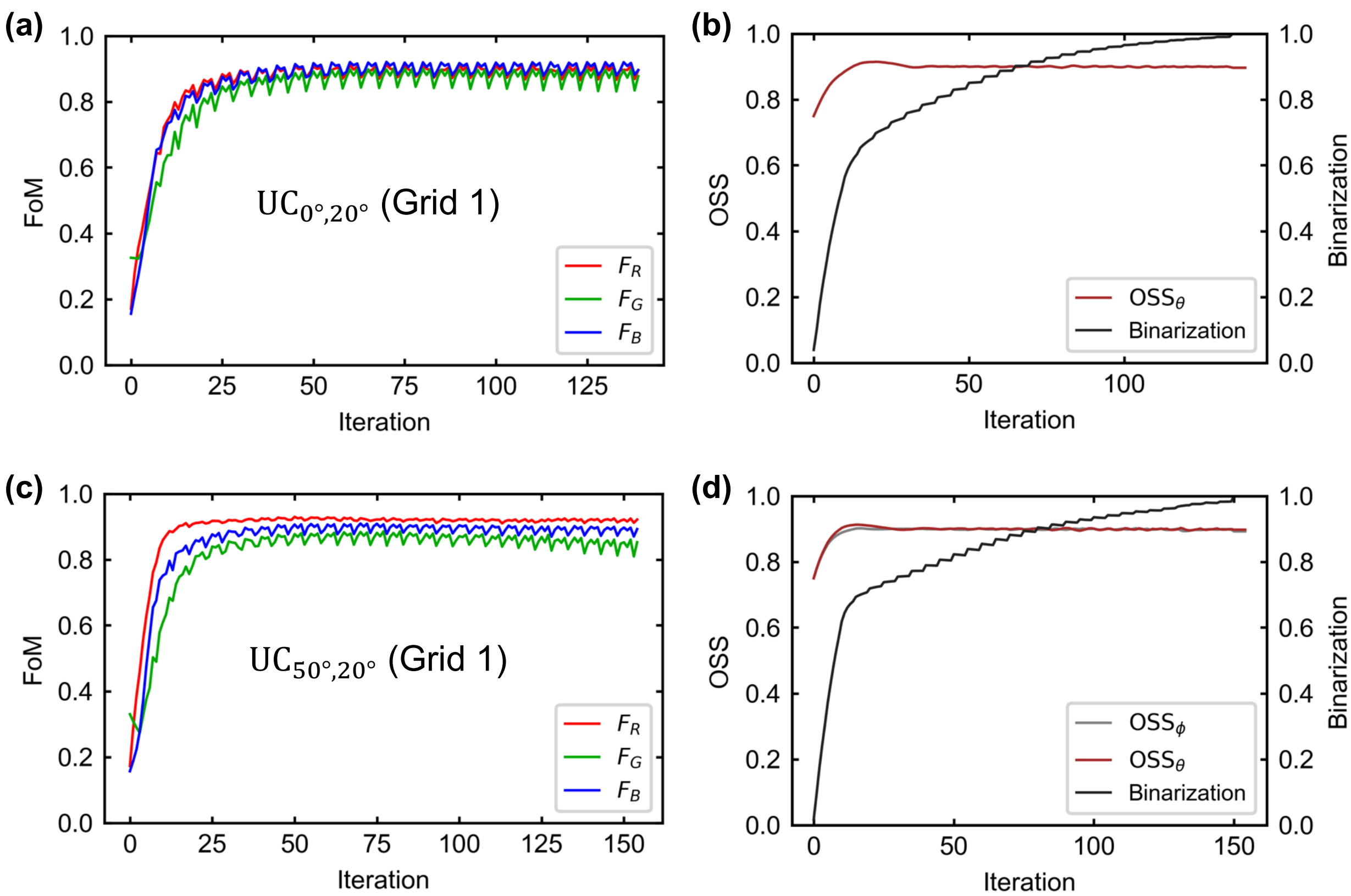

Fig S16: Representative optimization histories of OSS-constrained 3D freeform unit cells on Grid 1 with $\tau = 0.90$. Panels (a) and (c) show the iteration-wise evolution of the red-, green-, and blue-channel figures of merit for $\mathrm{UC}_{0°,20°}$ and $\mathrm{UC}_{50°,20°}$, respectively. Panels (b) and (d) show the corresponding OSS metric(s) and binarization metric. The results indicate stable convergence of the optical objective while satisfying the imposed structural-similarity constraint.

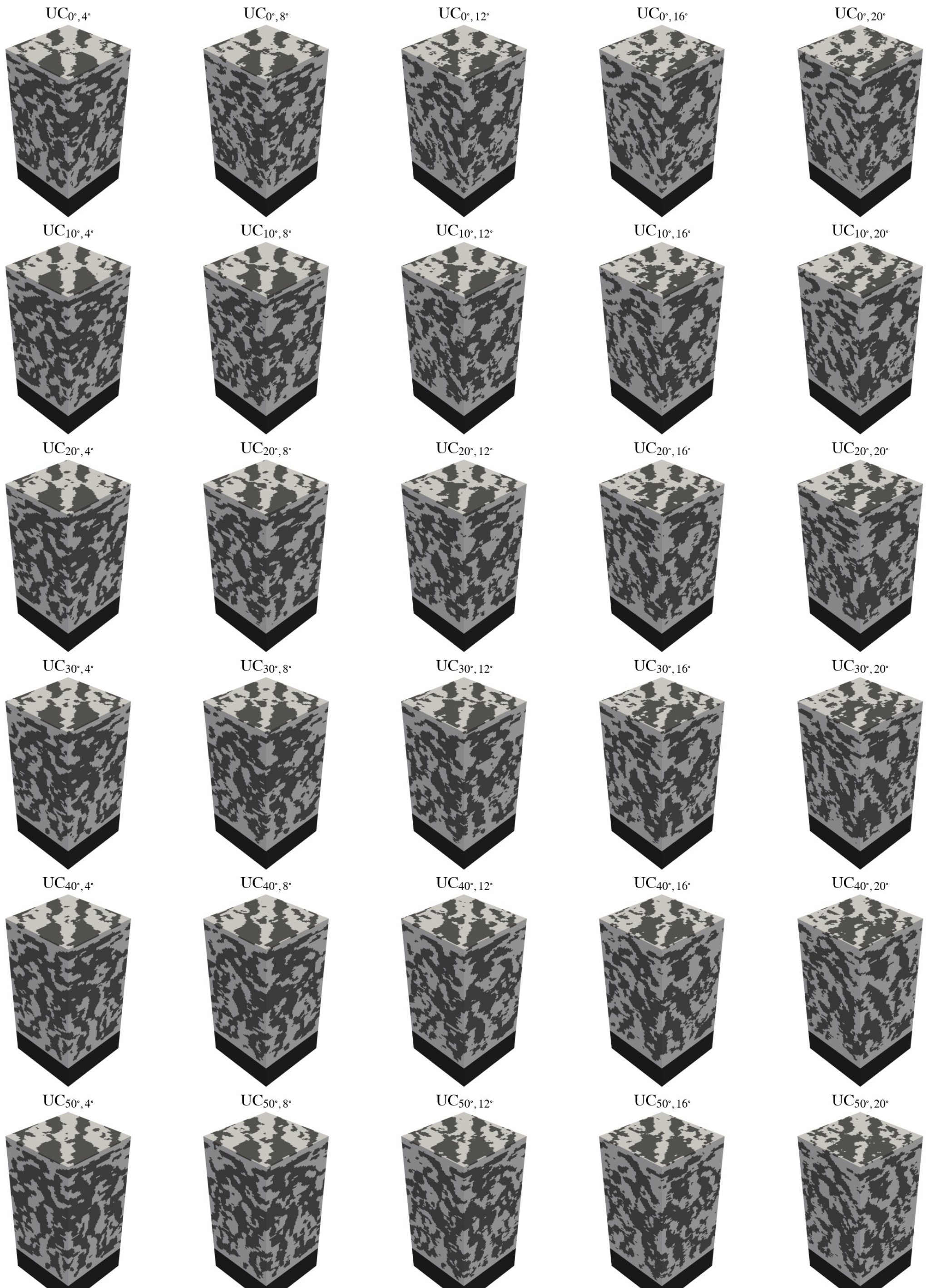

Fig S17: Full OSS-constrained 3D freeform unit-cell library on Grid 1 [$(\Delta\phi, \Delta\theta) = (10^\circ, 4^\circ)$, $\tau = 0.90$]. The 30 optimized unit cells correspond to $\phi_0 = 0^\circ, 10^\circ, \ldots, 50^\circ$ and $\theta_0 = 4^\circ, 8^\circ, 12^\circ, 16^\circ, 20^\circ$. Neighboring cells evolve smoothly across both the azimuthal and polar directions, reflecting the structural continuity enforced by the OSS constraint during sequential library construction.

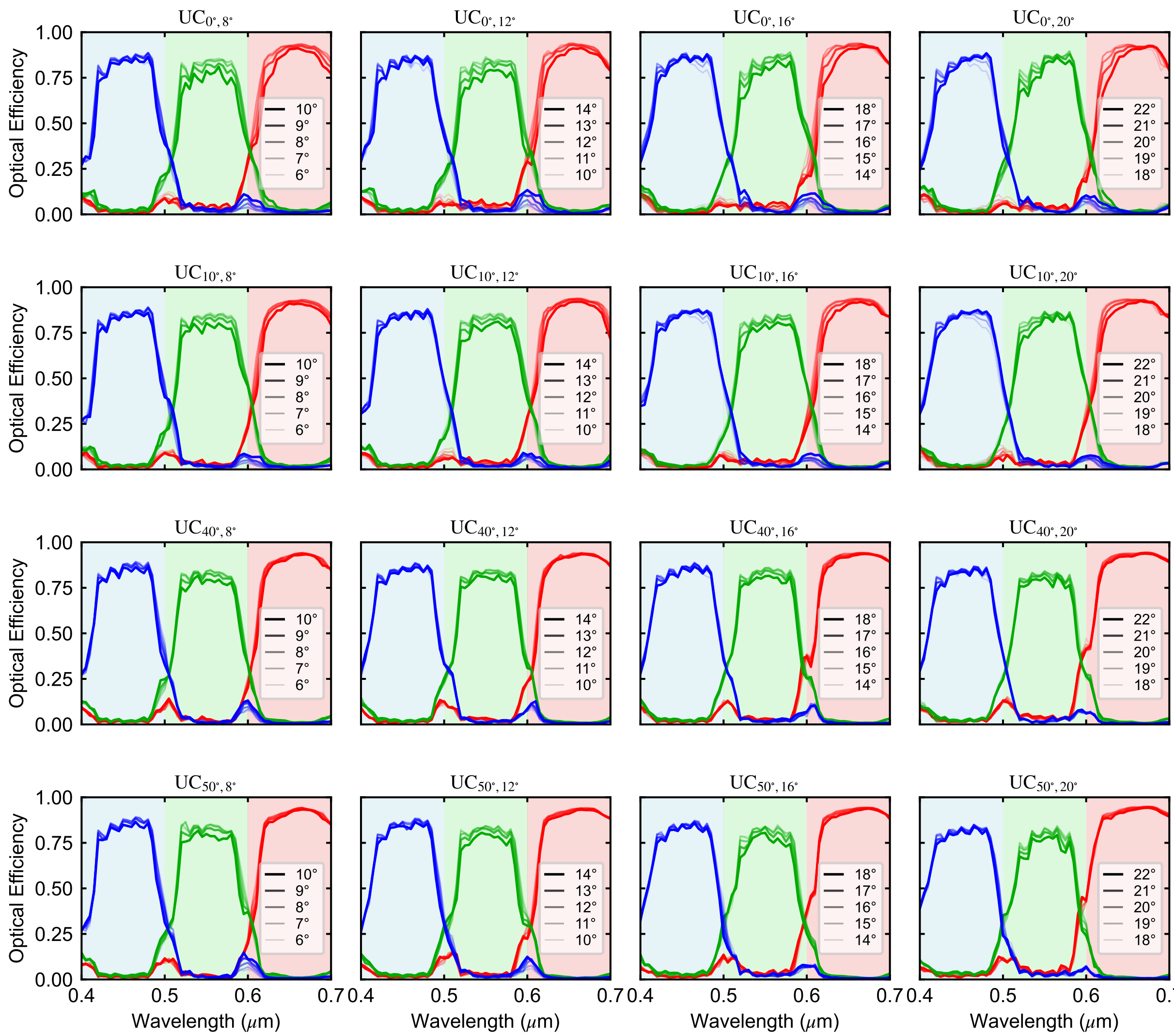

Fig S18: Isolated optical-efficiency spectra of representative OSS-constrained unit cells on Grid 1. The selected cells are $\mathrm{UC}_{\phi_0,\theta_0}$ for $\phi_0 \in \{0°, 10°, 40°, 50°\}$ and $\theta_0 \in \{8°, 12°, 16°, 20°\}$, corresponding to the same set of 16 cells used in Figure S12. Each panel reports the spectra over the sampled polar-angle set assigned to that unit cell, demonstrating that strong angle-specific color routing is retained despite the structural continuity enforced by OSS.

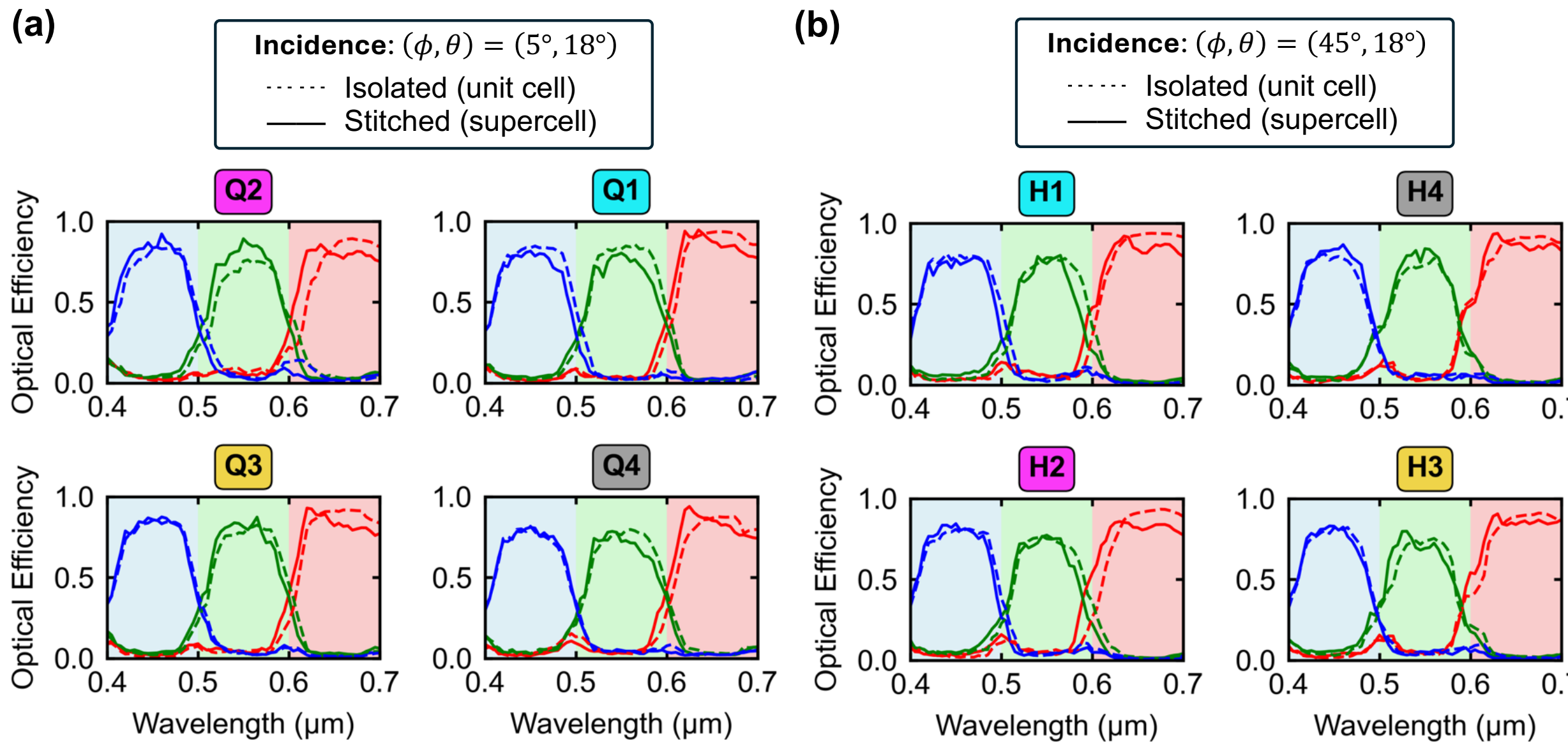

Fig S19: Additional stitched-supercell validation of the OSS-constrained 3D freeform library on Grid 1 $[(\Delta\phi, \Delta\theta) = (10°, 4°), \tau = 0.90]$. The two boundaries shown here complement Figure 5 of the main text and complete the four-boundary Grid 1 validation: (a) Q-type at $(\phi, \theta) = (5°, 18°)$ and (b) H-type at $(\phi, \theta) = (45°, 18°)$. In both cases, the stitched spectra of the boundary-adjacent cells remain close to the isolated unit-cell references across the red, green, and blue channels.

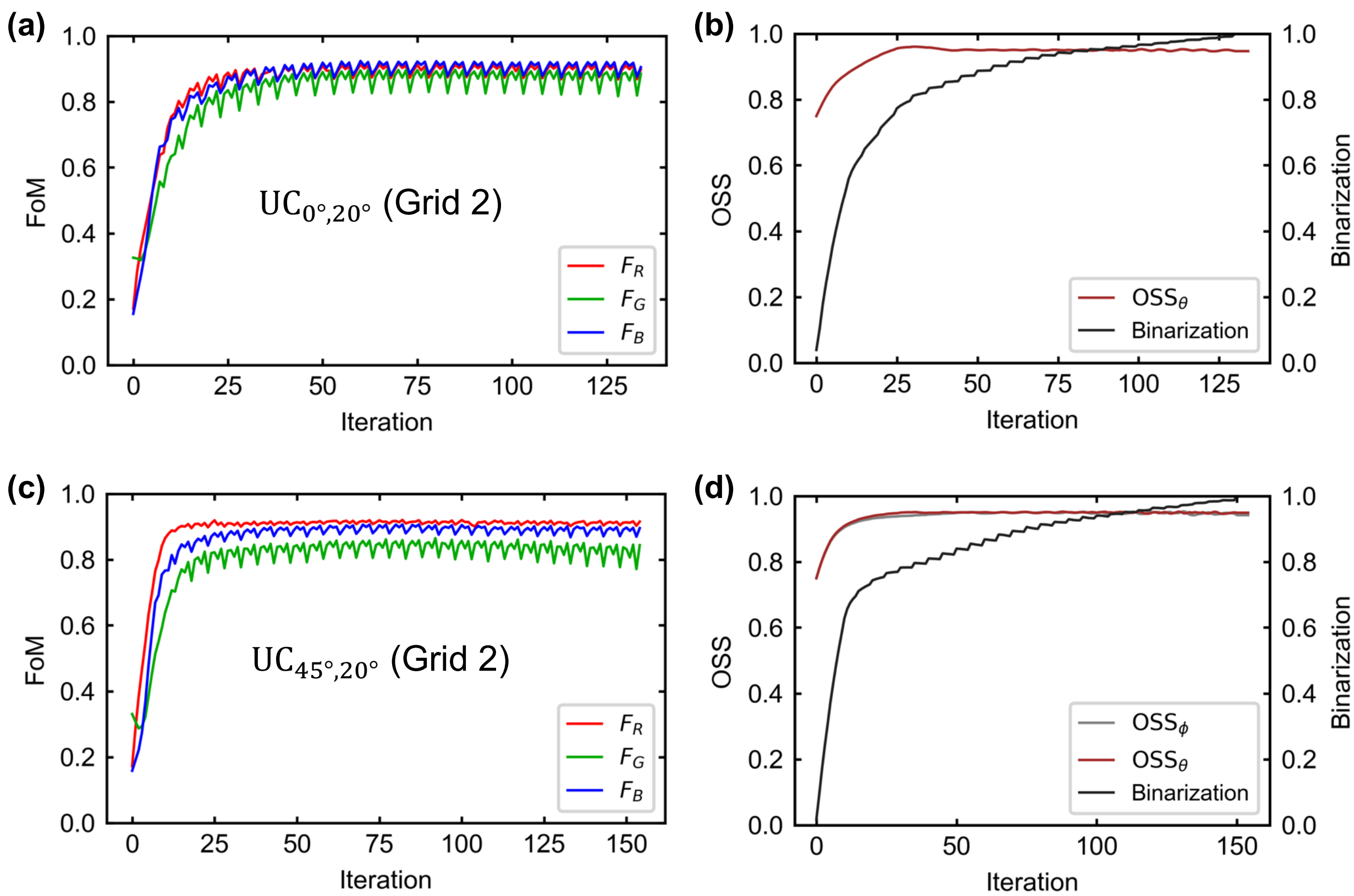

Fig S20: Representative optimization histories of OSS-constrained 3D freeform unit cells on Grid 2 with $\tau = 0.95$. Panels (a) and (c) show the iteration-wise evolution of the red-, green-, and blue-channel figures of merit for $\mathrm{UC}_{0°,20°}$ and $\mathrm{UC}_{45°,20°}$, respectively. Panels (b) and (d) show the corresponding OSS metric(s) and binarization metric. The results confirm stable convergence under the stricter OSS requirement used in Grid 2.

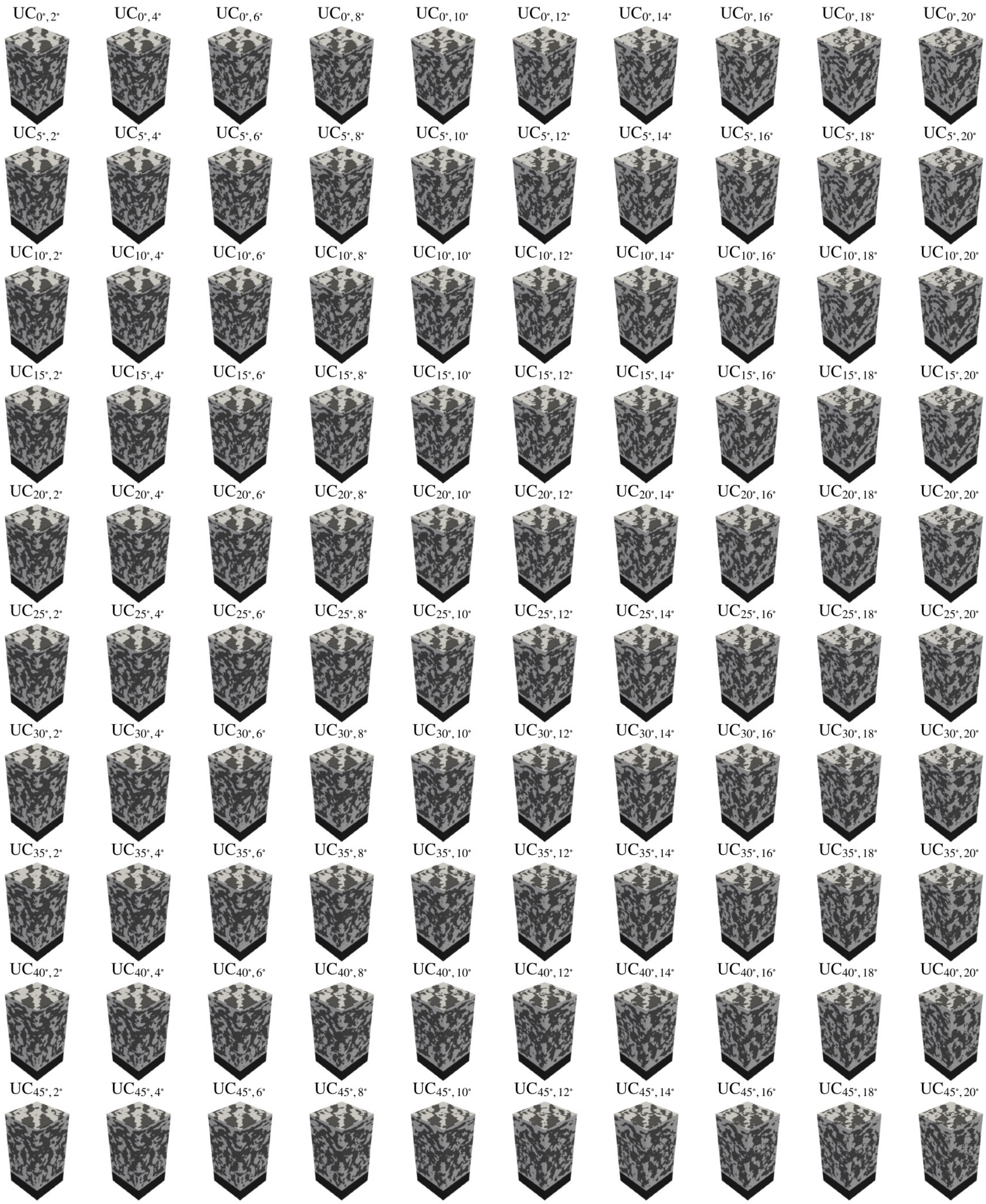

Fig S21: Full OSS-constrained 3D freeform unit-cell library on Grid 2 [$(\Delta\phi, \Delta\theta) = (5°, 2°)$, $\tau = 0.95$]. The 100 optimized unit cells correspond to $\phi_0 = 0°, 5°, \ldots, 45°$ and $\theta_0 = 2°, 4°, \ldots, 20°$. Relative to Grid 1, the finer angular discretization yields a denser and even more gradual structural evolution across neighboring angular zones.

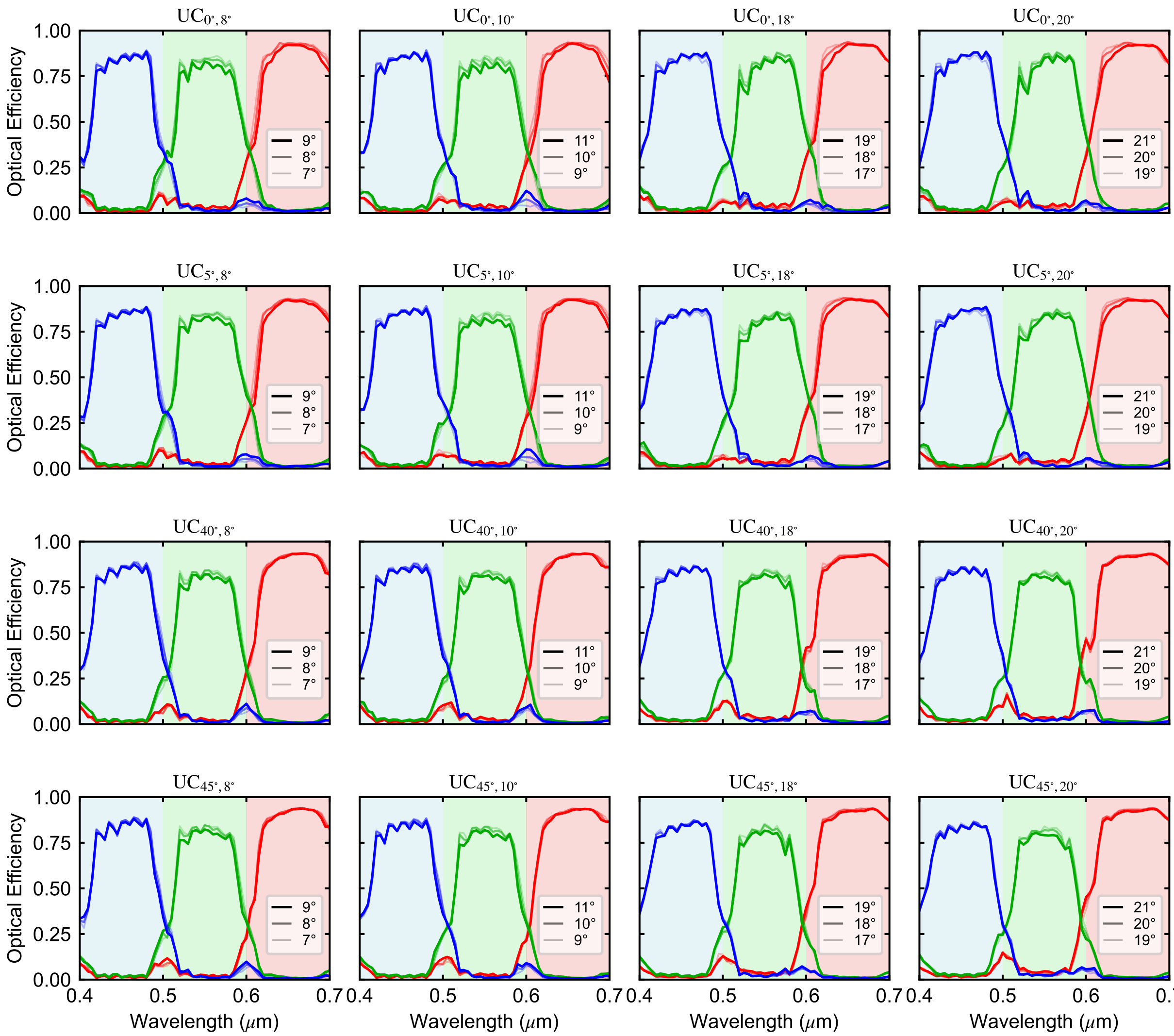

Fig S22: Isolated optical-efficiency spectra of representative OSS-constrained unit cells on Grid 2. The selected cells span low- and high-azimuth branches ($\phi_0 = 0°$, $5°$, $40°$, and $45°$) and representative polar angles ($\theta_0 = 8°$, $10°$, $18°$, and $20°$). The isolated spectra remain clearly separated over the sampled angular sets assigned to each unit cell.

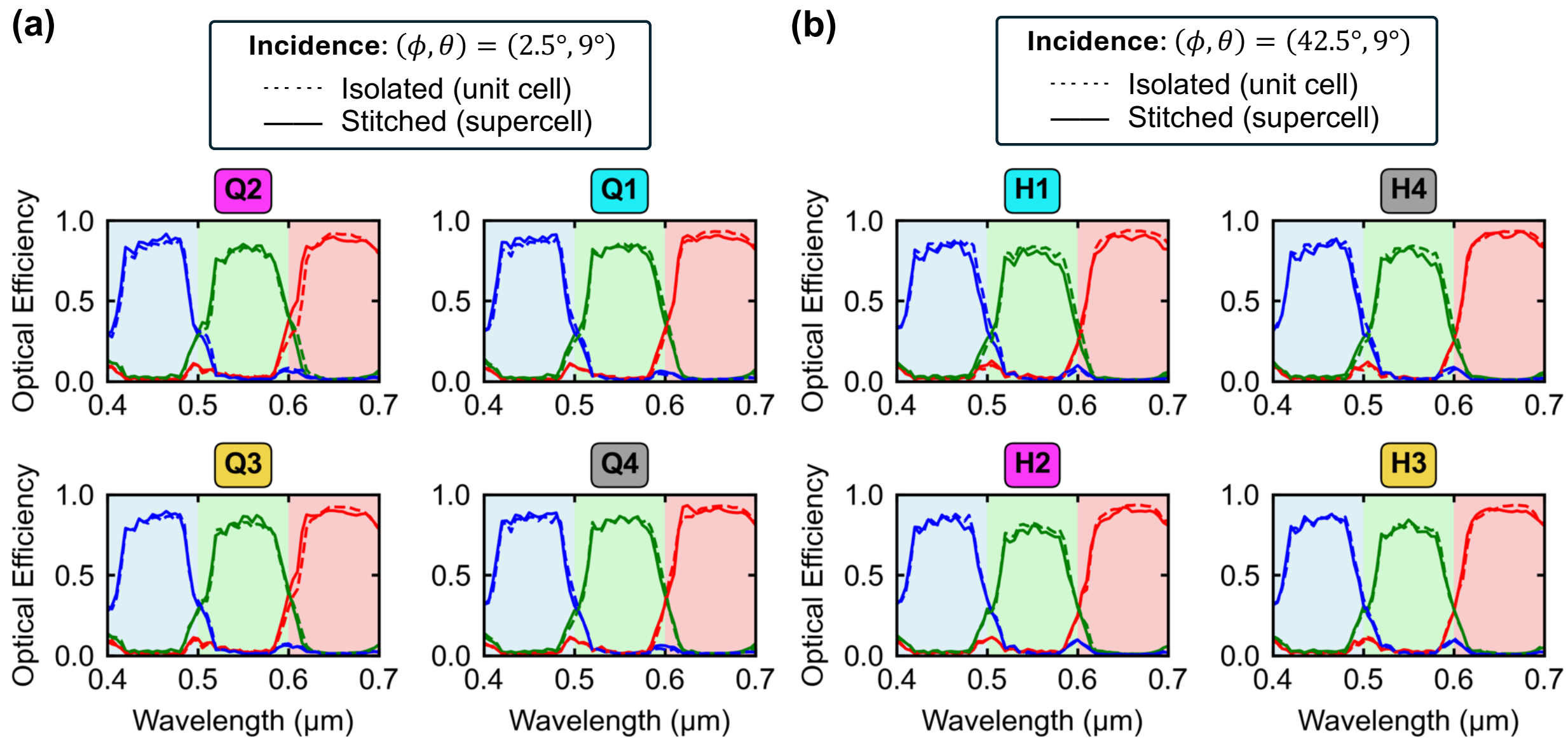

Fig S23: Additional stitched-supercell validation of the OSS-constrained 3D freeform library on Grid 2 [$(\Delta\phi, \Delta\theta) = (5^\circ, 2^\circ)$, $\tau = 0.95$]. The two boundaries shown here complement Figure 6 of the main text and complete the four-boundary Grid 2 validation: (a) Q-type at $(\phi, \theta) = (2.5^\circ, 9^\circ)$ and (b) H-type at $(\phi, \theta) = (42.5^\circ, 9^\circ)$. In both cases, the stitched spectra closely follow the isolated references, confirming that stitching-induced perturbations are strongly suppressed.

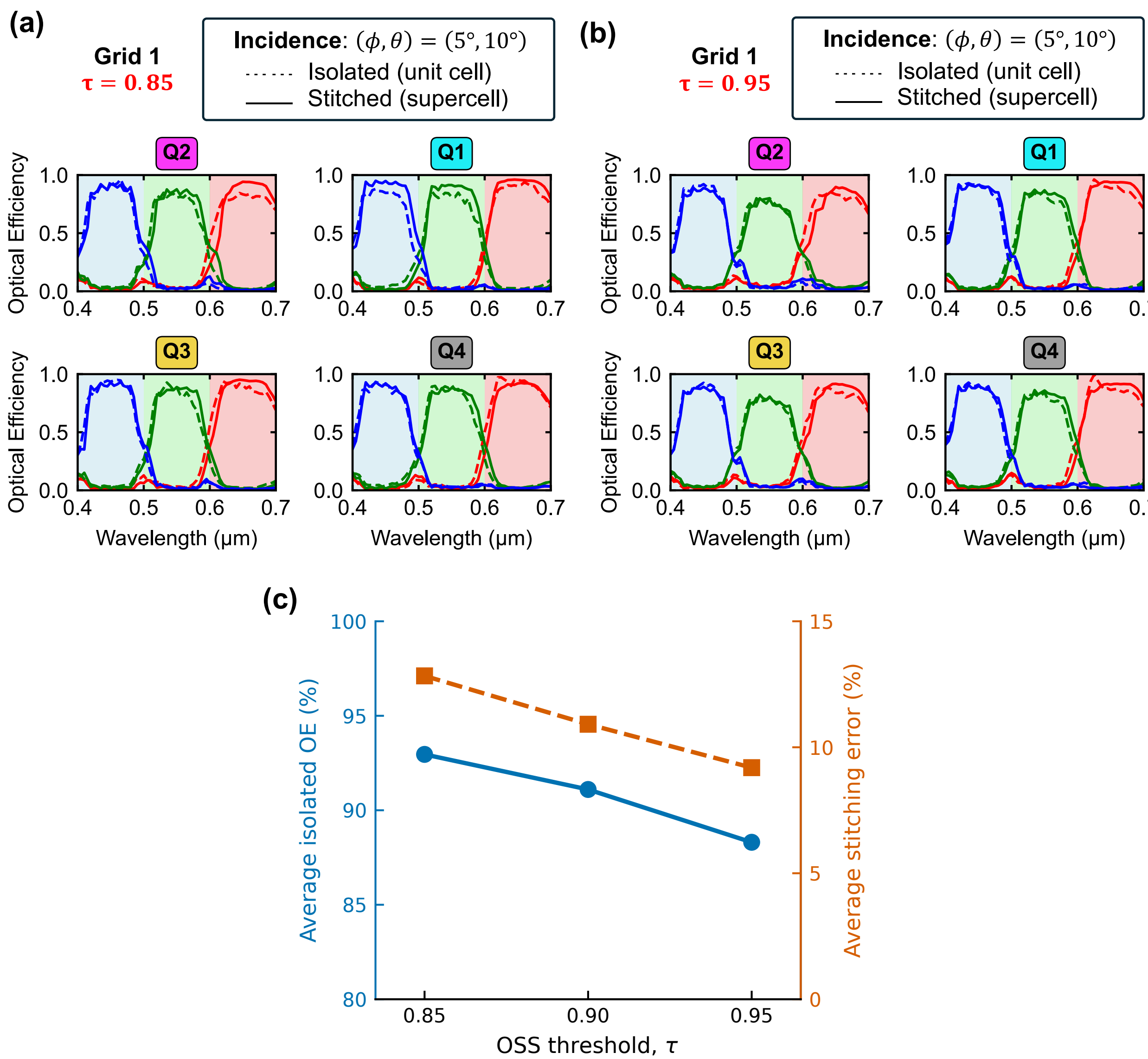

Fig S24: Sensitivity of the OSS-constrained design to the structural-similarity threshold τ, evaluated for the Grid 1 four-zone Q-type junction at $(\phi, \theta) = (5^\circ, 10^\circ)$, corresponding to the boundary configuration shown in Figure 5 of the main text. The four boundary-adjacent cells $\mathrm{UC}_{0^\circ,8^\circ}$, $\mathrm{UC}_{0^\circ,12^\circ}$, $\mathrm{UC}_{10^\circ,8^\circ}$, and $\mathrm{UC}_{10^\circ,12^\circ}$ (Q1–Q4) were re-optimized using $\tau = 0.85, 0.90$, and 0.95. (a,b) Isolated unit-cell spectra (dashed) and stitched-supercell spectra (solid) of Q1–Q4 for (a) $\tau = 0.85$ and (b) $\tau = 0.95$; the $\tau = 0.90$ case corresponds to the Grid 1 result reported in the main text. (c) Average isolated optical efficiency and average stitching error of the four boundary-adjacent cells as functions of τ. The results show a smooth trade-off between optical efficiency and stitching-error suppression, and all OSS-constrained cases reduce the stitching error to less than half of the unconstrained value (27.6%).

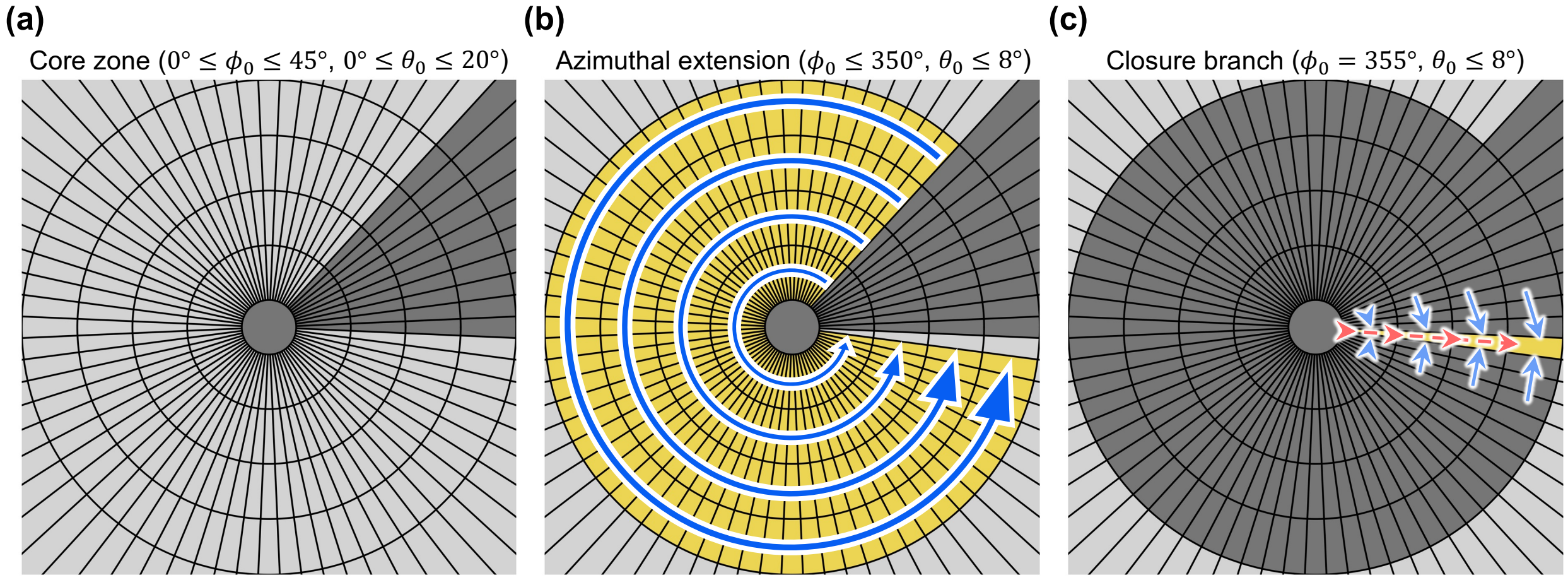

Fig S25: Angular library construction for full 360° azimuthal extension of the low-polar-angle Grid 2 branches. (a) Core zone: the previously reported Grid 2 library covering $\phi_0 = 0^\circ$–45° and $\theta_0 = 2^\circ$–20°, together with the normal-incidence seed cell at $\theta_0 = 0^\circ$ (dark gray). (b) Azimuthal extension: the circular branch explicitly optimized up to $\phi_0 = 350^\circ$ for the four smallest polar zones, $\theta_0 \in \{2^\circ, 4^\circ, 6^\circ, 8^\circ\}$ (yellow). (c) Closure branch: the final wrap-around step at $\phi_0 = 355^\circ$, where the terminal cells are optimized against three reference neighbors—the $\phi_0 = 350^\circ$ and $\phi_0 = 0^\circ$ azimuthal neighbors and the corresponding polar neighbor—to enforce seamless continuity at the azimuthal seam.

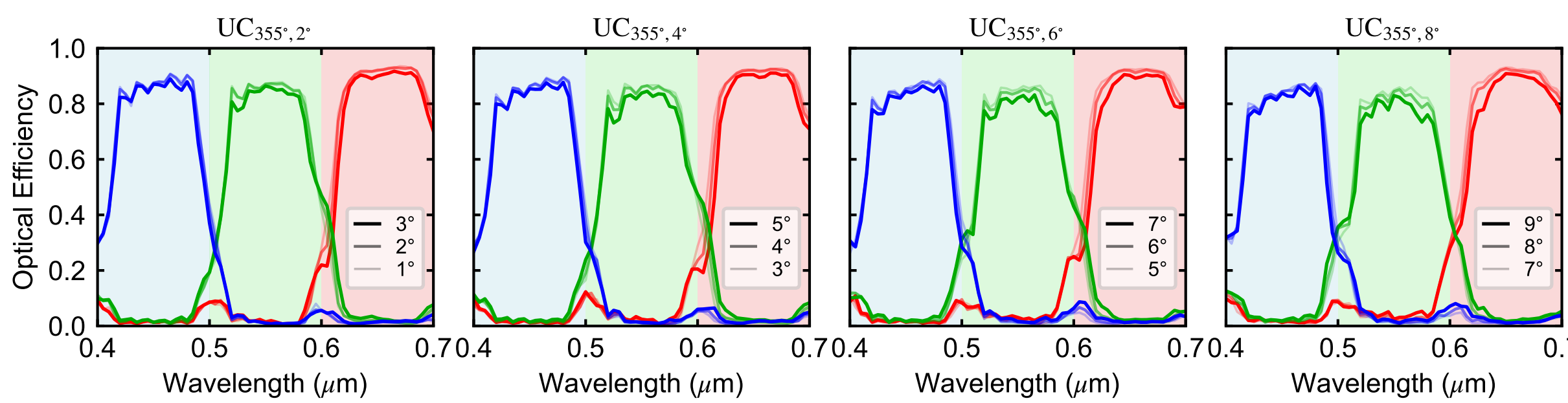

Fig S26: Isolated optical-efficiency spectra of the wrap-around closure cells $\mathrm{UC}_{355^\circ,2^\circ}$, $\mathrm{UC}_{355^\circ,4^\circ}$, $\mathrm{UC}_{355^\circ,6^\circ}$, and $\mathrm{UC}_{355^\circ,8^\circ}$ from the circular branch. Each panel reports the spectra over the sampled polar-angle set assigned to that unit cell: $\theta = 1^\circ$–3°, 3°–5°, 5°–7°, and 7°–9°, respectively. In all four cases, the red, green, and blue target-channel responses remain clearly separated, confirming that simultaneous three-reference OSS-constrained closure preserves strong angle-specific color routing at the wrap-around seam.

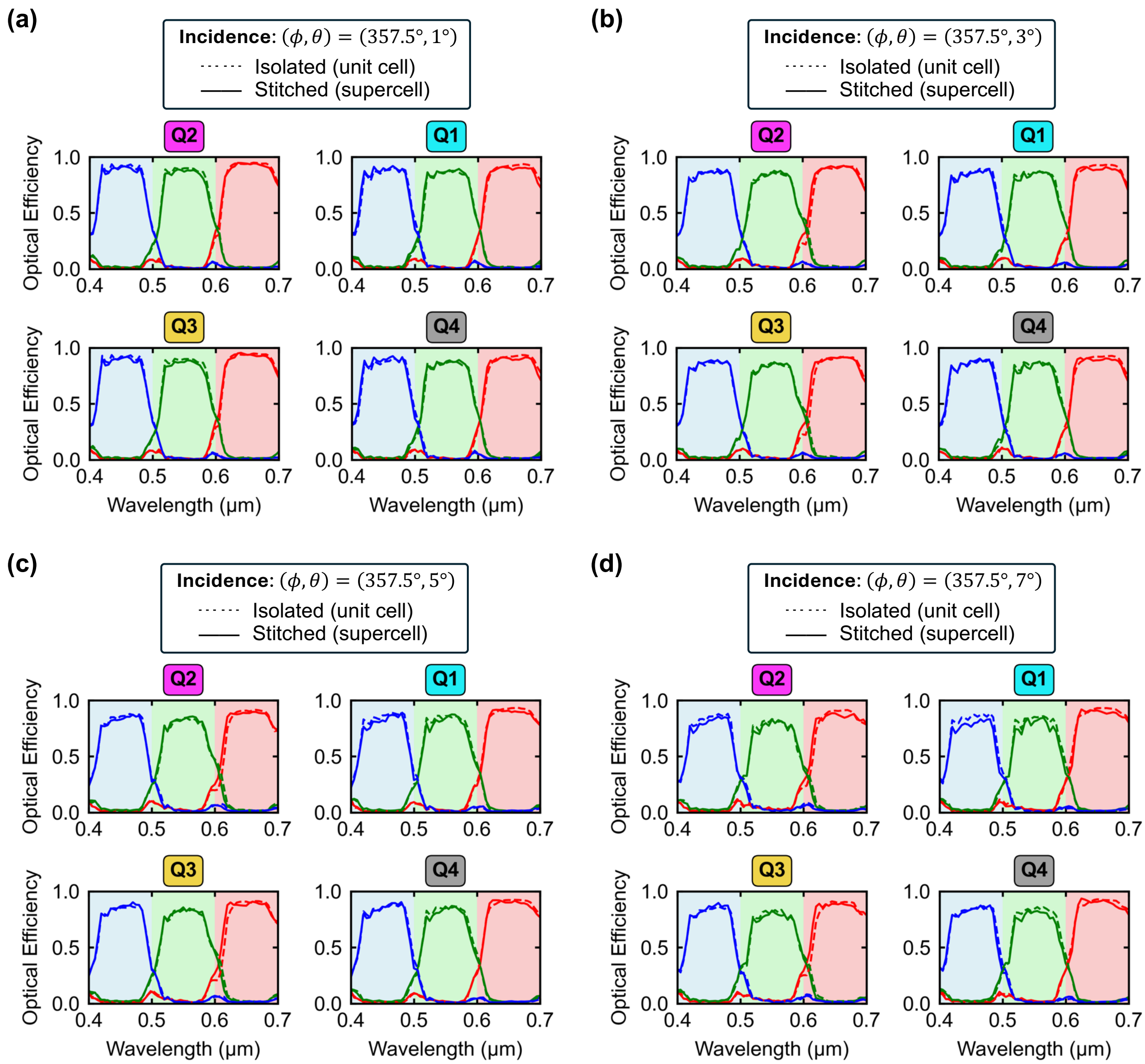

Fig S27: Stitched-supercell validation of the wrap-around closure boundary at $\phi = 357.5^\circ$ for the four low-polar-angle branches of the circular branch on Grid 2 $[(\Delta\phi, \Delta\theta) = (5^\circ, 2^\circ),\ \tau = 0.95]$. (a)–(d) Q-type boundaries at incidence angles $(\phi, \theta) = (357.5^\circ, 1^\circ)$, $(357.5^\circ, 3^\circ)$, $(357.5^\circ, 5^\circ)$, and $(357.5^\circ, 7^\circ)$, respectively. In each panel, dashed curves denote isolated unit-cell responses under Bloch boundary conditions and solid curves denote the stitched-supercell responses of the four boundary-adjacent cells Q1–Q4. Across all four polar zones, the stitched spectra closely track the isolated references in the red, green, and blue channels, confirming that the OSS framework extends naturally to the full azimuthal circle without introducing stitching-induced degradation at the wrap-around seam.

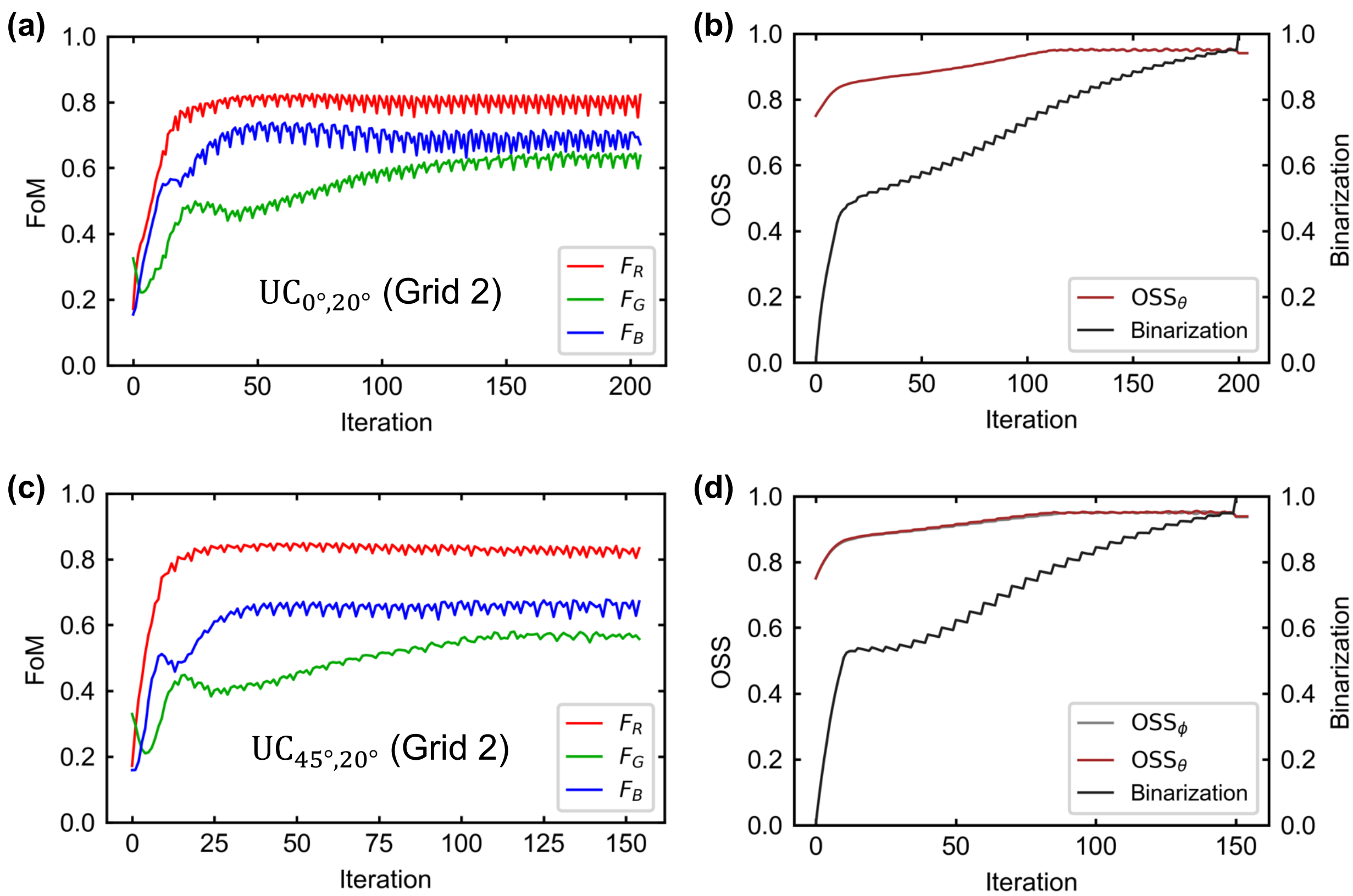

Fig S28: Representative optimization histories of five-layer OSS-constrained unit cells. Panels (a) and (c) show the iteration-wise evolution of the red-, green-, and blue-channel figures of merit for two representative cells, $\mathrm{UC}_{0^\circ,20^\circ}$ and $\mathrm{UC}_{45^\circ,20^\circ}$, respectively, and panels (b) and (d) show the corresponding OSS metric(s) and binarization metric. Despite the reduced geometric degrees of freedom of the layered parameterization, the optical objective converges stably while the structural-similarity constraint is maintained.

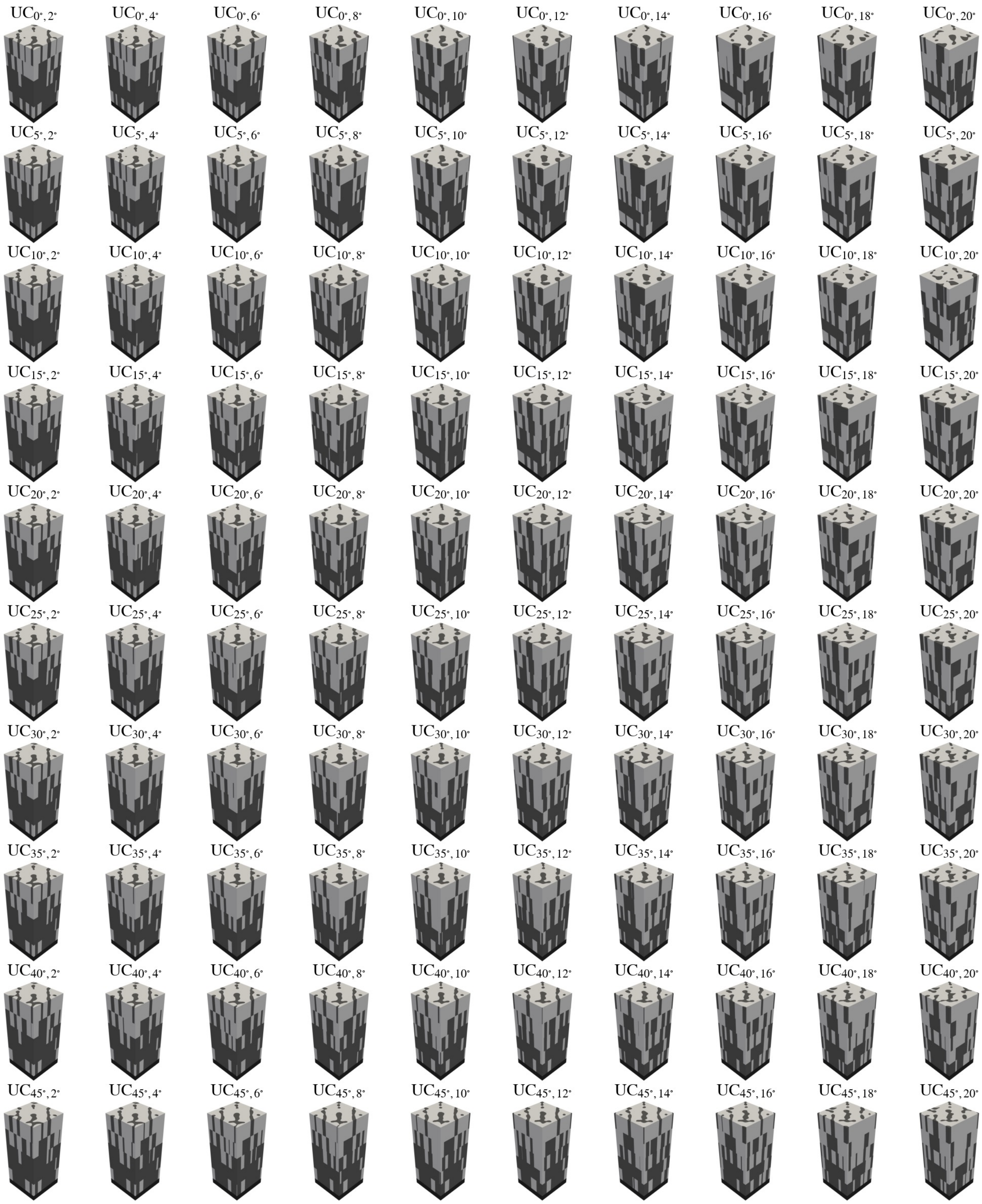

Fig S29: Five-layer OSS-constrained unit-cell library on Grid 2 [$(\Delta\phi, \Delta\theta) = (5^\circ, 2^\circ)$, $\tau = 0.95$]. All 100 optimized unit cells are shown. The design region retains the same overall footprint as the freeform case but is partitioned into five vertically stacked layers, yielding a reduced-complexity architecture with visually smooth structural evolution across angular space.

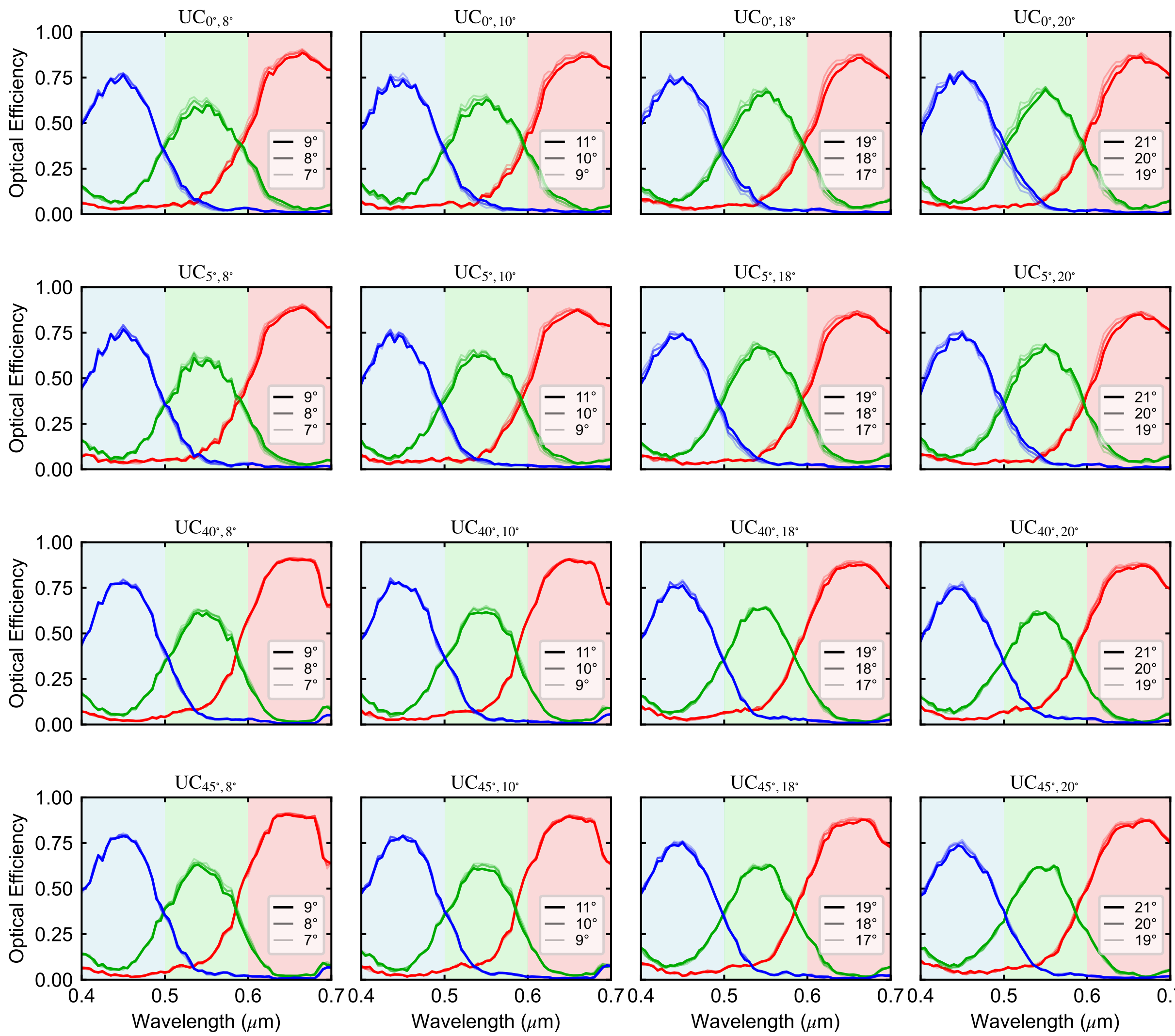

Fig S30: Isolated optical-efficiency spectra of representative unit cells from the five-layer library. The same low- and high-azimuth branches ($\phi_0 = 0°$, $5°$, $40°$, and $45°$) and representative polar angles ($\theta_0 = 8°$, $10°$, $18°$, and $20°$) used for the freeform Grid 2 validation are adopted here to enable direct comparison. Relative to the corresponding freeform cases in Figure S22, the five-layer spectra exhibit broader transitions and somewhat lower peak efficiencies, while still maintaining clear color-channel separation within the assigned angular ranges.

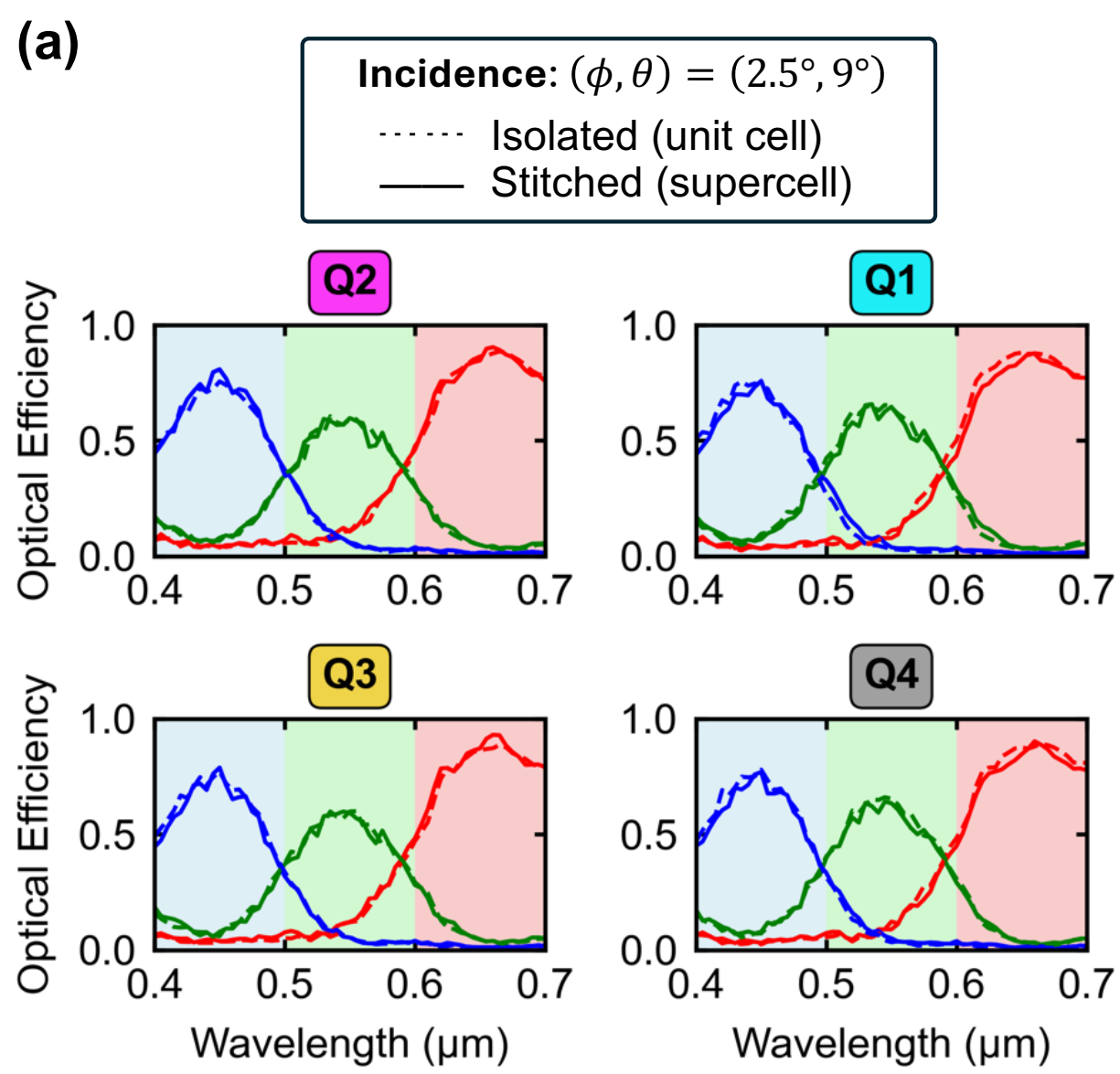

Fig S31: Additional stitched-supercell validation of the five-layer OSS-constrained library on Grid 2 [$(\Delta\phi, \Delta\theta) = (5°, 2°)$, $\tau = 0.95$]. The two boundaries shown here complement Figure 7 of the main text, which reports the $\theta = 19°$ cases: (a) Q-type at $(\phi, \theta) = (2.5°, 9°)$ and (b) H-type at $(\phi, \theta) = (42.5°, 9°)$. In both cases, the stitched spectra remain close to the isolated references, indicating that the OSS framework preserves strong stitching robustness even in the reduced-complexity layered architecture.

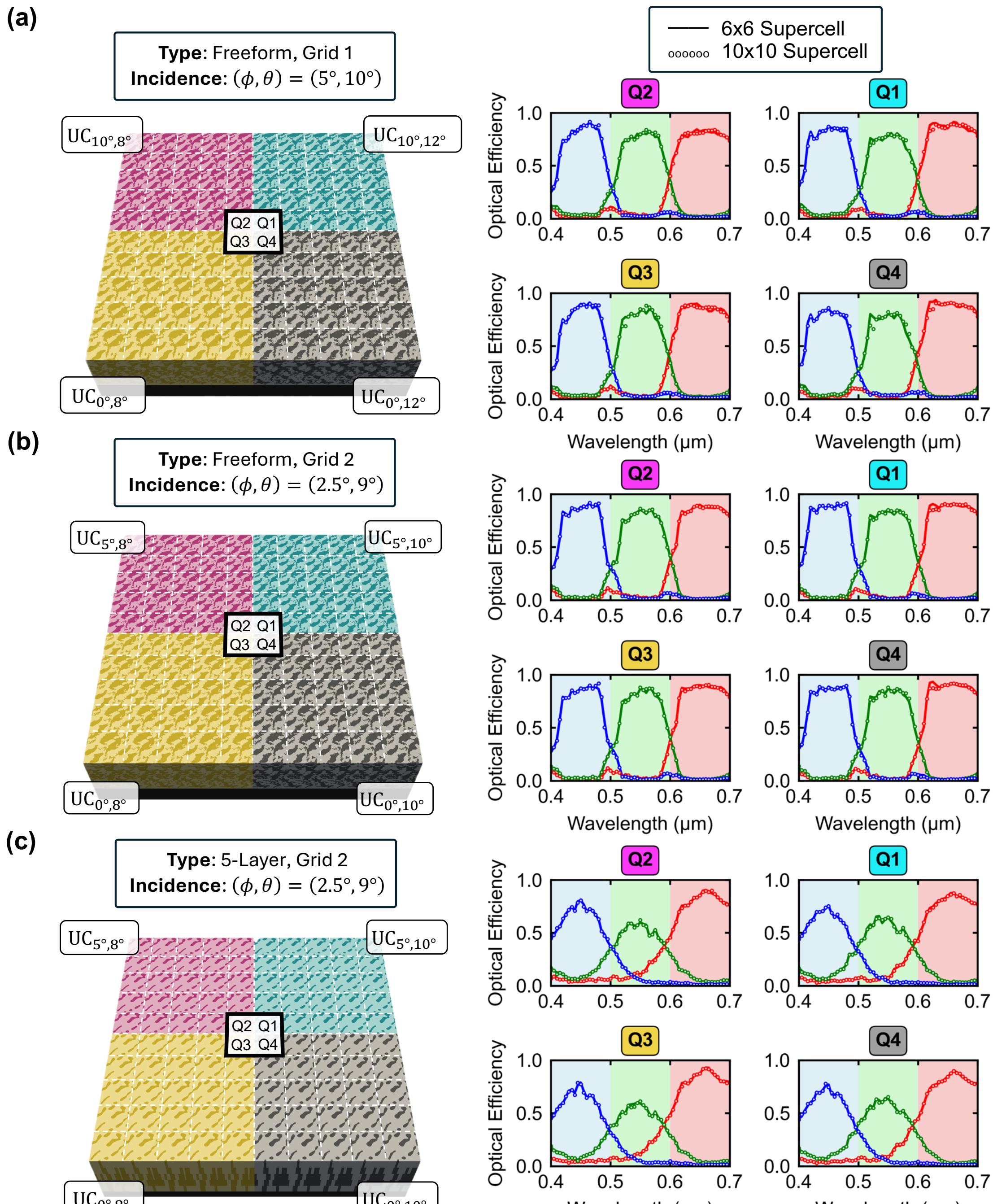

Fig S32: Supercell-size convergence of stitched-boundary simulations. Optical-efficiency spectra extracted from boundary-adjacent cells Q1–Q4 are compared between 6×6 stitched supercells, shown as solid lines, and 10×10 stitched supercells, shown as open circular symbols. The comparison is shown for representative cases: (a) Freeform Grid 1 at $(\phi, \theta) = (5^\circ, 10^\circ)$, (b) Freeform Grid 2 at $(\phi, \theta) = (2.5^\circ, 9^\circ)$, and (c) 5-layer Grid 2 at $(\phi, \theta) = (2.5^\circ, 9^\circ)$. In each case, the two supercell sizes are compared under the same local boundary geometry and incidence condition. The close spectral agreement confirms that the 6×6 supercell provides a converged estimate of the local stitched-boundary response.

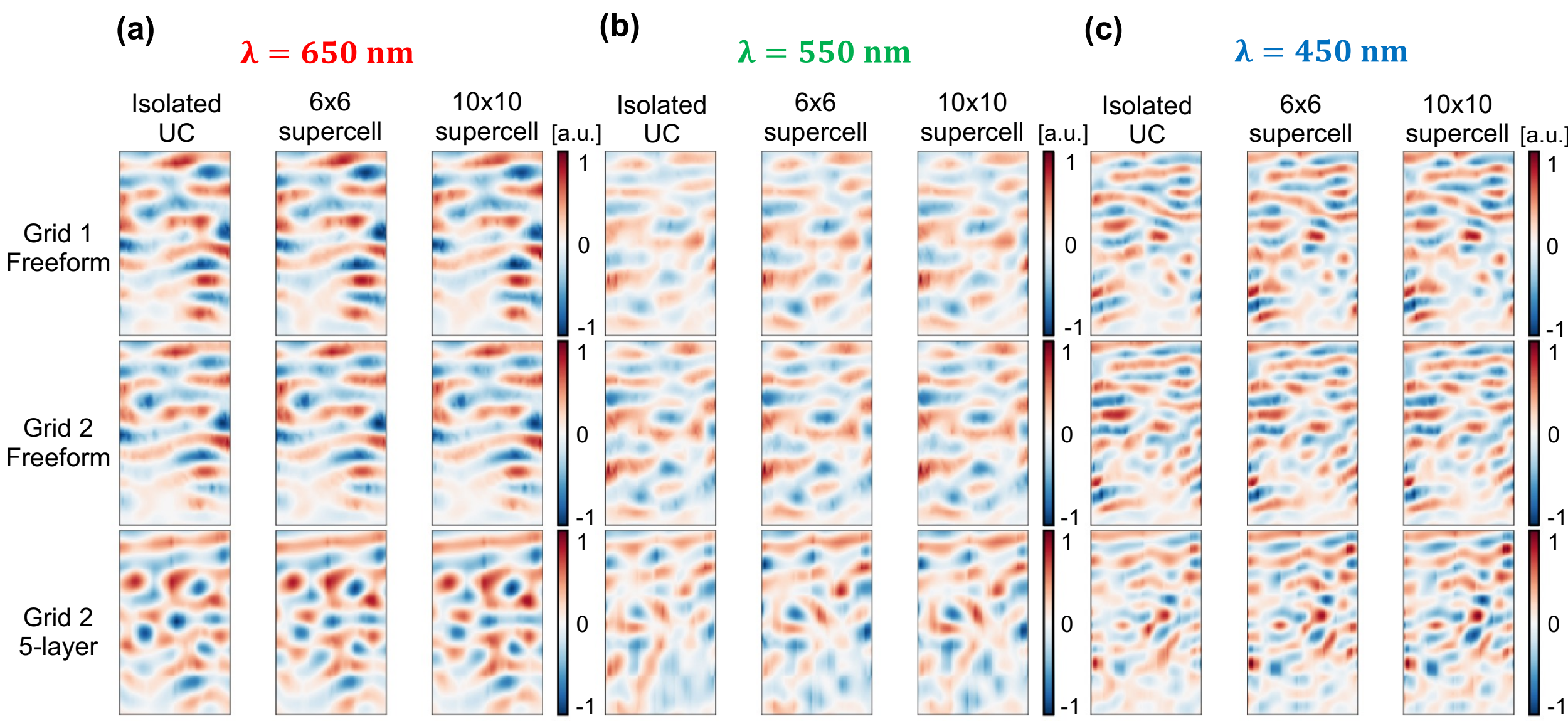

Fig S33: Field-profile convergence for representative Q1 boundary-adjacent cells. XZ cross-sections of $\mathrm{Re}(\mathbf{E})$ are shown at (a) $\lambda = 650$ nm, (b) $\lambda = 550$ nm, and (c) $\lambda = 450$ nm for the isolated Bloch-periodic unit cell, the corresponding cell embedded in a 6×6 stitched supercell, and the corresponding cell embedded in a 10×10 stitched supercell. Rows correspond to the Freeform Grid 1, Freeform Grid 2, and 5-layer Grid 2 cases. For each case and wavelength, the field maps are normalized by the maximum absolute $\mathrm{Re}(\mathbf{E})$ value among the isolated, 6×6, and 10×10 fields.

References

1 Alec M. Hammond, Ardavan Oskooi, Steven G. Johnson … Stephen E. Ralph, "Photonic topology optimization with semiconductor-foundry design-rule constraints," *Optics Express* **29**(15), 23916–23938 (2021).

2 Fengwen Wang, Boyan S. Lazarov, and Ole Sigmund, "On projection methods, convergence and robust formulations in topology optimization," *Structural and Multidisciplinary Optimization* **43**(6), 767–784 (2011).

3 Diederik P. Kingma and Jimmy Ba, "Adam: A method for stochastic optimization," in *Proceedings of the 3rd International Conference on Learning Representations (ICLR)*, (2015).
